# Modelling the impact of mosquito bed net utilization on malaria transmission and evolution of pyrethroid resistance

**DOI:** 10.1371/journal.pone.0353301

**Published:** 2026-07-15

**Authors:** Ivan Sseguya, Joseph Y. T. Mugisha, Juliet N. Nakakawa, Prashanth Selvaraj, Jonathan Kayondo

**Affiliations:** 1 Mathematics Department, Makerere University, Kampala, Uganda; 2 Institute for Disease Modeling, Gates Foundation, Seattle, Washington, United States of America; 3 Entomology Department, Uganda Virus Research Institute, Entebbe, Uganda; World Health Organization, Regional Office for South-East Asia, INDIA

## Abstract

Long-lasting Insecticide Nets (LLINs) are central to malaria prevention, but their effectiveness is threatened by the increasing pyrethroid resistance and low bed net utilization in malaria-endemic regions globally. This study formulates a genotype-specific compartmental model accounting for malaria transmission. It incorporates the blocking and insecticidal killing effects of LLINs, and pyrethroid-induced fitness costs, to evaluate the impact of LLIN utilization on malaria and resistance dynamics. Reproductive numbers with and without LLINs were derived, and sensitivity analysis performed. This analysis showed that the utilization of LLINs and their effectiveness against pyrethroid-resistant vectors have the strongest effect in reducing malaria transmission in both Sobol and partial rank correlation coefficient PRCC analyses. The baseline scenario and four LLIN utilization scenarios (standard, three-year, two-year and one-year) were simulated over nine years to track malaria transmission, mosquito population and evolution of resistance. These were assessed, with pyrethroid-only nets deployed in the first two campaigns and pyrethroid piperonyl butoxide (PBO) nets deployed in the third campaign. Results showed that, despite an initial LLIN coverage of 81%, protection against malaria infection by both pyrethroid-only and pyrethroid-PBO nets was not sustained over the campaign period and this worsened with lower utilization levels. Pyrethroid-PBO nets provided greater mosquito suppression and better protection under standard utilization, but their advantage over pyrethroid-only nets declined with lower utilization. The study further demonstrated that repeated pyrethroid-only campaigns accelerate the evolution of pyrethroid resistance, but PBO nets counteract this trend particularly when such resistance carries a higher fitness cost. Although lower utilization levels slowed resistance evolution, these scenarios do not represent an epidemiologically viable malaria control strategy.

## 1. Introduction

Malaria is a major global threat, accounting for an estimated 597 000 deaths in 2023 with a mortality rate of 13.7 per 1000 people, despite the substantial gains in the past two decades [1]. The World Health Organization (WHO) African region persistently bears the greatest burden, representing about 94% of the global malaria cases and 95% of malaria-related deaths worldwide by 2024 [2]. In 2023, Uganda registered the world’s third highest malaria burden, accounting for about 5.0% of all global cases and 15,945 deaths [1]. According to the 2025 World Malaria Report, this country retained its third position with about 4.7% of the global malaria cases [2]. The disease is endemic in 95% of the country, contributing between 30% to 50% of outpatient visits, 15% to 20% of all hospital admissions, and up to 20% of all hospital deaths [3].

Vector control forms the backbone for malaria prevention efforts [4], and it aims to eliminate malaria-transmitting vectors or inhibit their ability to blood feed [5]. Primary vector control tools include Long-Lasting Insecticidal Nets (LLINs) and Indoor Residual Spraying (IRS) [3], but other tools like larviciding, larval source management, and improved housing are used in public health malaria campaigns [6–8]. These are widely implemented and have demonstrated high effectiveness when used in malaria public health campaigns [9,10]. For instance, a meta-analysis found that IRS led to a 65% reduction in malaria risk overall, and a 73% reduction when coverage was at least 80% [10], and the use of LLINs across sub-Saharan Africa between 2000 and 2015 led to a 68% reduction in malaria cases of 663 million cases averted globally [11]. In Uganda, IRS usage led to a 2.2→9.0 percentage reduction in malaria morbidity, measured by slide positivity rate, during the first three months following its application [12,13]. However, this effect waned by the fourth to sixth month under field conditions [13].

Despite of the LLIN effectiveness [11], their continued usage or wide coverage exert high levels of selection pressure on malaria vectors, causing them to evolve resistance towards insecticides like pyrethroids, commonly used in malaria public health interventions [14]. This has compromised the long-term effectiveness of LLIN campaigns over years [15], with such resistance reported in many countries including Uganda [16], Ghana [17], Malawi [18], Kenya [19] among others. In an effort to address this challenge, next generational nets like Interceptor^®^ G2 which combines pyrethroids with Chlorfenapyr [20], PermaNet^®^ 3.0 and Olyset^®^ Plus which combine pyrethroids with piperonyl butoxide (PBO), Royal Guard^®^ Net which combines pyrethroids and pyriproxyfen, and DawaPlus^®^ 3.0 which combines pyrethroids with PBO, have been developed to improve LLIN effectiveness in high insecticide-resistance settings [21]. The success of these bed net campaigns depends on the level of effective coverage, vector behaviour patterns, timely replacement, and chemical and physical durability of the bed nets [22,23]. LLIN utilization remains low, especially within African countries [24,25], despite their potential in reducing malaria transmission. In Uganda, field studies showed that the adequate coverage of LLINs substantially decreased from 71% at baseline to less than half of this coverage after 25 months [26] due to LLIN attrition after distribution. Additionally, a modelling analysis found that 35 out of 40 African countries have a median bed net retention time of 1.64 years, with Uganda having a less than 2-year average bed net retention time [27].

Mathematical models provide a powerful framework to analyse disease dynamics and have been used to assess the impact of LLIN interventions on malaria transmission [28–30] and to study the development and spread of insecticide-resistance within the mosquito population [31,32]. While there are several studies that have addressed related questions on LLIN utilization [33–35], limited work has focused on the impact of bed net utilization patterns on malaria transmission dynamics and evolution of pyrethroid resistance. This study investigates how temporal decay in bed net utilization impacts malaria burden and the evolution of pyrethroid resistance within the mosquito population. Therefore, results from this study can help national malaria control programs to refine LLIN campaign strategies aimed at reducing malaria transmission and slowing emergence of pyrethroid resistance.

## 2. Model description and formulation

This section formulated a mathematical model which was investigated analytically and numerically through simulations. First, a deterministic model system with constant parameters was formulated to allow analytical investigation of epidemiological thresholds like malaria free equilibrium (MFE) and the basic reproductive number. This formulation excludes time-dependent intervention effects and seasonal forcing to maintain mathematical tractability. For numerical simulations, the model was extended to include seasonal mosquito recruitment, fitness costs, and to allow selected parameters to vary over time. LLIN coverage, killing effectiveness and blocking effects were expressed as time dependent functions to capture deployment, utilization decay, and waning insecticidal effectiveness, while mosquito recruitment was represented as a seasonal function. These modifications enabled the model to simulate a more realistic transmission dynamics under intervention settings.

Mohammed-Awel and Gumel [36] formulated a genotype-specific epidemiology model that combines malaria transmission dynamics with the evolution of insecticide resistance in mosquito populations. It incorporates human – vector interactions and insecticide effects, and tracks resistance evolution along mosquito genotypes. This formulation captures the dual dynamics of malaria and resistance evolution, providing a flexible structure to include ecological and intervention constraints. Therefore, we modify this model by consolidating the human population and incorporating waning immunity, malaria induced mortality and density-dependent mosquito recruitment. The modified model categorizes the human population into susceptible humans ($S_{h}$), infectious humans $I_{h}$ and those recovered from malaria infection upon treatment or natural recovery $R_{h}$. Therefore, the total human population $N_{h}$ is given by; $N_{h}=S_{h}+I_{h}+R_{h}$.The susceptible population $S_{h}$ grows at a constant natality rate of $\Lambda_{h}$ and rate ϑ as recovered individuals lose their temporary immunity. However, this reduces due to new malaria infections obtained through indoor or outdoor biting at rates $\rho_{in}$ and $\rho_{out}$ respectively, and natural mortality at the rate $\mu_{h}$.

An allele refers to one of two or more versions of DNA sequence at a given genomic location, while a genotype is a genetic constitution of an organism, particularly a combination of alleles at one or more loci [37,38]. In this formulation, the mosquito population is considered to have two alleles types in its genetic pool: pyrethroid sensitive allele *S* and pyrethroid resistant allele *R*, with corresponding allele frequencies *p*(*t*) and *q*(*t*). The vector population, in model system (2), is categorized according to the stage of infection (i.e., susceptible, latently infected, and infectious mosquitoes), and genotype (i.e., homozygous sensitive *SS*, heterozygous *SR*, and homozygous resistant *RR*). The total vector population is classified into nine epidemiological compartments which include: homozygous sensitive susceptible $S_{SS}$, latently infected $E_{SS}$, and infectious $I_{SS}$ mosquitoes; heterozygous susceptible $S_{SR}$, latently infected $E_{SR}$, and infectious $I_{SR}$ mosquitoes; and homozygous resistant susceptible $S_{RR}$, latently infected $E_{RR}$, and infectious $I_{RR}$ for pyrethroid resistant vectors. With this grouping, it follows that; $N_{v}=N_{SS}+N_{SR}+N_{RR}$, where $N_{SS}=S_{SS}+E_{SS}+I_{SS}$, $N_{SR}=S_{SR}+E_{SR}+I_{SR}$ and $N_{RR}=S_{RR}+E_{RR}+I_{RR}$. Recruitment of new mosquitoes is modelled using a logistic function of the form $\Lambda_{v}N_{v}(1-\frac{N_{v}}{K})$, where $\Lambda_{v}$ denotes the intrinsic mosquito recruitment rate, and *K* is the environmental carrying capacity for the mosquito population. At recruitment, the proportion of new homozygous sensitive, heterozygous and homozygous resistant genotypes at any time *t*, is given by *p*^2^(*t*), 2*p*(*t*)*q*(*t*) and *q*^2^(*t*) respectively, where allele frequencies *p*(*t*) and *q*(*t*) [39] are given by:


$$\begin{array}{c} p(t)=\frac{N_{SS}(t)+0.5N_{SR}(t)}{N_{v}(t)},\text{and}\;q(t)=\frac{N_{RR}(t)+0.5N_{SR}(t)}{N_{v}(t)}, \end{array}$$ (1)

with $N_{v}(t)$, $N_{SS}(t)$, $N_{SR}(t)$ and $N_{RR}(t)$ maintaining their original meaning.

**Fig 1:**
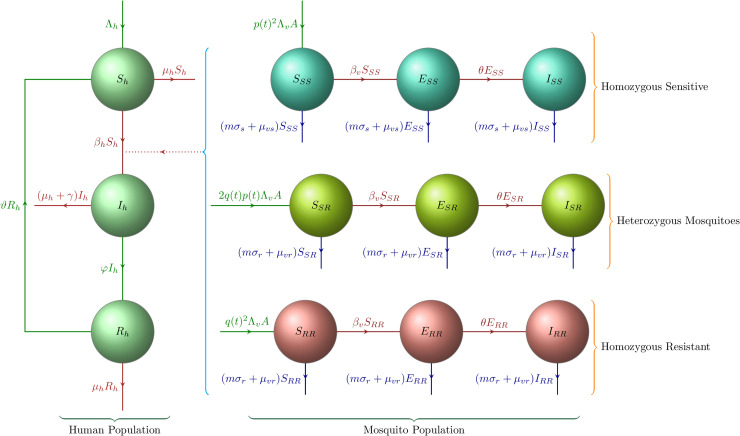
Compartmental diagram for the mosquito genetic classification and malaria transmission between human and vector populations.

The deployment of bed nets interrupt malaria transmission through blocking mosquitoes from blood feeding at a rate ${\text{LLIN}}_{bloc}$, and killing malaria vectors upon making physical contact with bed nets at rates ${\text{LLIN}}_{eff}$ and ${\text{LLIN}}_{effr}$ for sensitive and resistant mosquitoes respectively. This formulation is supported by the assumptions: humans have the same risk of exposure to malaria infection regardless of age, humans acquire no adaptive immunity over years, female vectors have enough males to fertilize them [40], mosquitoes are uniformly distributed with equal access to human hosts, and there is no change in mosquito behaviour as a response to LLIN campaigns. The human and mosquito interaction, and malaria transmission are represented in the compartmental diagram in Fig 1, where the logistic term $A=N_{v}(1-\frac{N_{v}}{K})$, human force of infection $\beta_{h}=\frac{\tau_{h}(I_{SS}+I_{SR}+I_{RR})}{N_{h}}(m\beta_{bloc}\rho_{in}+(1-m)\rho_{out})$ and mosquito force of infection $\beta_{v}=\frac{\tau_{v}I_{h}}{N_{h}}(m\beta_{bloc}\rho_{in}+(1-m)\rho_{out})$ respectively. This compartmental diagram in Fig 1 leads to the system of model equations:


$$\begin{array}{c} \begin{array}{cc} \frac{dS_{h}}{dt}= & \Lambda_{h}+\vartheta R_{h}-\frac{\tau_{h}(I_{SS}+I_{SR}+I_{RR})S_{h}}{N_{h}}(m\beta_{bloc}\rho_{in}+(1-m)\rho_{out})-\mu_{h}S_{h},\\ \frac{dI_{h}}{dt}= & \frac{\tau_{h}(I_{SS}+I_{SR}+I_{RR})S_{h}}{N_{h}}(m\beta_{bloc}\rho_{in}+(1-m)\rho_{out})-\varphi I_{h}-(\mu_{h}+\gamma )I_{h},\\ \frac{dR_{h}}{dt}= & \varphi I_{h}-\vartheta R_{h}-\mu_{h}R_{h},\\ \frac{dS_{SS}}{dt}= & p^{2}\Lambda_{v}N_{v}(1-\frac{N_{v}}{K})-\frac{\tau_{v}I_{h}S_{SS}}{N_{h}}(m\beta_{bloc}\rho_{in}+(1-m)\rho_{out})-(m\sigma_{s}+\mu_{vs})S_{SS},\\ \frac{dE_{SS}}{dt}= & \frac{\tau_{v}I_{h}S_{SS}}{N_{h}}(m\beta_{bloc}\rho_{in}+(1-m)\rho_{out})-\theta E_{SS}-(m\sigma_{s}+\mu_{vs})E_{SS},\\ \frac{dI_{SS}}{dt}= & \theta E_{SS}-(m\sigma_{s}+\mu_{vs})I_{SS},\\ \frac{dS_{SR}}{dt}= & 2pq\Lambda_{v}N_{v}(1-\frac{N_{v}}{K})-\frac{\tau_{v}I_{h}S_{SR}}{N_{h}}(m\beta_{bloc}\rho_{in}+(1-m)\rho_{out})-(m\sigma_{r}+\mu_{vr})S_{SR},\\ \frac{dE_{SR}}{dt}= & \frac{\tau_{v}I_{h}S_{SR}}{N_{h}}(m\beta_{bloc}\rho_{in}+(1-m)\rho_{out})-\theta E_{SR}-(m\sigma_{r}+\mu_{vr})E_{SR},\\ \frac{dI_{SR}}{dt}= & \theta E_{SR}-(m\sigma_{r}+\mu_{vr})I_{SR}.\\ \frac{dS_{RR}}{dt}= & q^{2}\Lambda_{v}N_{v}(1-\frac{N_{v}}{K})-\frac{\tau_{v}I_{h}S_{RR}}{N_{h}}(m\beta_{bloc}\rho_{in}+(1-m)\rho_{out})-(m\sigma_{r}+\mu_{vr})S_{RR}.\\ \frac{dE_{RR}}{dt}= & \frac{\tau_{v}I_{h}S_{RR}}{N_{h}}(m\beta_{bloc}\rho_{in}+(1-m)\rho_{out})-\theta E_{RR}-(m\sigma_{r}+\mu_{vr})E_{RR},\\ \frac{dI_{RR}}{dt}= & \theta E_{RR}-(m\sigma_{r}+\mu_{vr})I_{RR}, \end{array} \end{array}$$ (2)

where $\sigma_{s}={\text{LLIN}}_{cov}\times f\times {\text{LLIN}}_{eff}$, $\sigma_{r}={\text{LLIN}}_{cov}\times f\times {\text{LLIN}}_{effr}$, $\beta_{bloc}=1-({\text{LLIN}}_{cov}\times f\times {\text{LLIN}}_{bloc})$, ${\text{LLIN}}_{cov}$ is the LLIN coverage at population level, ${\text{LLIN}}_{eff}$ is the pyrethroid killing effectiveness, ${\text{LLIN}}_{bloc}$ is the LLIN blocking effect, *m* is the proportion of mosquito vectors that bite indoors and *f* is the proportion of indoor biting mosquitoes that make contact with bed nets.

## 3. Basic properties of the model

For analytical tractability, all model parameters in model system (2) – including the LLIN blocking effectiveness $\beta_{bloc}$, and pyrethroid induced death rates in sensitive and resistant mosquitoes $\sigma_{s}$ and $\sigma_{r}$ respectively are constant for all time. The solutions to this system remain positive and bounded within the epidemiological feasible region ξ for all t≥0, where population values remain non-negative and biologically realistic, ensuring that the system is mathematically well-posed. The mosquito population is regulated by the carrying capacity *K* while the human population dynamics are determined by $\Lambda_{h}-\mu_{h}$ (see subsection 6 in Appendix section). Therefore, these properties guarantee that long-term malaria predictions remain within biologically realistic limits.

### 3.1. Equilibrium points and malaria transmission reproductive number

This section presents the malaria free equilibrium point (MFE), existence of endemic steady states, and discusses the malaria transmission reproduction number with and without the use of LLIN bed net intervention. When no bed nets are deployed, the total mosquito biting rate $\omega =m\rho_{in}+(1-m)\rho_{out}$, and mosquito mortality rate $\mu_{v}=\mu_{vr}=\mu_{vs}$. Therefore, for $\Lambda_{v}>\mu_{v}$, the malaria free equilibrium point is given by; $E_{0}=(S_{h}^{*},I_{h}^{*},R_{h}^{*},S_{SS}^{*},E_{SS}^{*},I_{SS}^{*},S_{SR}^{*},E_{SR}^{*},I_{SR}^{*},S_{RR}^{*},$ $E_{RR}^{*},I_{RR}^{*})$, such that;


$$\begin{array}{c} E_{0}=(\frac{\Lambda_{h}}{\mu_{h}},0,0,\frac{p^{2}K(\Lambda_{v}-\mu_{v})}{\mu_{v}\Lambda_{v}},0,0,\frac{2pqK(\Lambda_{v}-\mu_{v})}{\mu_{v}\Lambda_{v}},0,0,\frac{q^{2}K(\Lambda_{v}-\mu_{v})}{\mu_{v}\Lambda_{v}},0,0). \end{array}$$

The Malaria Transmission reproduction number $R_{0}$ is obtained using the next generation matrix method [41], and given by:


$$\begin{array}{c} R_{0}=\sqrt{\frac{\omega \tau_{h}\Lambda_{h}}{\mu_{h}(\varphi +\gamma +\mu_{h})}}\times \sqrt{\frac{\omega \tau_{v}\theta K(\Lambda_{v}-\mu_{v})}{\mu_{v}\Lambda_{v}(\theta +\mu_{v})}}. \end{array}$$ (3)

The threshold $R_{0}$ in equation 3, represents the expected number of secondary malaria infections produced by a single infectious individual over the duration of infection provided that everyone else in the population is susceptible, in absence of bed net interventions. The square root on $R_{0}$ represents a two-phase infection process that occurs in malaria transmission across the human and vector population. This is because it requires two generations of infections, human to mosquito and vice versa, to obtain a secondary human infection from an infectious human [42]. The first square root term (infectious mosquitoes to new human infections) accounts for the expected number of infected humans an infectious mosquito will produce during its lifetime, depending on demographic factors like $\Lambda_{h}$ and $\Lambda_{v}$. Conversely, the second square root term (infectious humans to new mosquito infections) accounts for the expected number of infectious mosquitoes arising from one infectious human during his infectious period, scaled by the chance that a latently infected vector survives the exposure period and mosquito demographic parameters.

When a bed net campaign is deployed, the effective mosquito biting rate $\omega_{net}=m\beta_{bloc}\rho_{in}+(1-m)\rho_{out}$, $\mu_{vr}\text{≠}\mu_{vs}$ and the new malaria transmission reproduction number $R_{0}^{L}$ is given by:


$$\begin{array}{c} \begin{array}{cc} R_{0}^{L} & =R_{0}^{h}\times R_{0}^{v},\text{\textasciitilde{}\textasciitilde{}where},\\ R_{0}^{h} & =\sqrt{\frac{\tau_{h}\Lambda_{h}\omega_{net}}{\mu_{h}(\varphi +\gamma_{h}+\mu_{h})}},\\ R_{0}^{v} & =\sqrt{\frac{\theta \tau_{v}p^{2}\omega_{net}N_{v}^{*}(K-N_{v}^{*})}{K(m\sigma_{s}+\mu_{vs}{)}^{2}(\theta +m\sigma_{s}+\mu_{vs})}+\frac{q(2p+q)\tau_{v}\Lambda_{v}\omega_{net}N_{v}^{*}(K-N_{v}^{*})}{K(m\sigma_{r}+\mu_{vr}{)}^{2}(\theta +m\sigma_{r}+\mu_{vr})}} \end{array} \end{array}$$

and $N_{v}^{*}$ is the total mosquito population at MFE. Therefore, $R_{0}^{L}$ represents the expected number of secondary malaria infections produced by a single infectious individual over the duration of infection, provided that everyone else in the population is susceptible, when a bed net campaign is implemented. This depends on, but not limited to, impact of LLINs in blocking mosquitoes from blood feeding, mosquito biting rate, the human recovery and death rate etc. $R_{0}^{v}$ is modified with terms $\frac{\theta \tau_{v}p^{2}\omega_{net}N_{v}^{*}(K-N_{v}^{*})}{K(m\sigma_{s}+\mu_{vs}{)}^{2}(\theta +m\sigma_{s}+\mu_{vs})}$ and $\frac{q(2p+q)\tau_{v}\Lambda_{v}\omega_{net}N_{v}^{*}(K-N_{v}^{*})}{K(m\sigma_{r}+\mu_{vr}{)}^{2}(\theta +m\sigma_{r}+\mu_{vr})}$ representing the expected number of infectious pyrethroid sensitive and resistant vectors respectively, arising from an infectious human during his infectious period, adjusted by the rate of LLIN killing, progression into infectious state and natural mosquito mortality. Since the system (2) satisfies axioms (A1) to (A5) in [41] and $R_{0}^{L}$ is biologically meaningful, using Theorem 2 of [41] the result below holds;

**Result 1.** *The MFE state for system (2) is locally asymptotically stable if* $R_{0}<1$ *and unstable if* $R_{0}>1$*.*

Result (1) means that malaria infection can be eliminated from human and mosquito population when $R_{0}<1$ if the initial sizes of the sub-populations of system (2) are in the basin of attraction of the malaria – free steady state.

Generally, the equilibrium points of the model system 2 are given by:


$$\begin{array}{c} \begin{array}{cc} S_{h}^{*}= & \frac{\Lambda_{h}(\vartheta +\mu_{h})(\vartheta +\gamma +\mu_{h})}{\left(\vartheta +\mu_{h})(\mu_{h}+\gamma )(\mu_{h}+F_{hv}^{*})+\mu_{h}\varphi (F_{hv}^{*}+\vartheta +\mu_{h}\right)},\;S_{SS}^{*}=\frac{p^{2}K(\Lambda_{v}-\mu_{v})}{\Lambda_{v}(F_{vh}^{*}+m\sigma_{s}+\mu_{v})},\\ I_{h}^{*}= & \frac{F_{hv}^{*}F_{hv}^{*}(\vartheta +\mu_{h})}{\mu_{h}\varphi F_{hv}^{*}+(\vartheta +\mu_{h})[(\mu_{h}+\gamma )F_{hv}^{*}+(\vartheta +\gamma +\mu_{h})\mu_{h}]},\;S_{RR}^{*}=\frac{q^{2}K(\Lambda_{v}-\mu_{v})}{\Lambda_{v}(F_{vh}^{*}+m\sigma_{r}+\mu_{v})},\\ R_{h}^{*}= & \frac{\varphi \Lambda F_{hv}^{*}}{\mu_{h}\varphi F_{hv}^{*}+(\vartheta +\mu_{h})[(\mu_{h}+\gamma )F_{hv}^{*}+(\vartheta +\gamma +\mu_{h})\mu_{h}]},\;S_{SR}^{*}=\frac{2pqK(\Lambda_{v}-\mu_{v})}{\Lambda_{v}(F_{vh}^{*}+m\sigma_{r}+\mu_{v})},\\ E_{SS}= & \frac{p^{2}K(\Lambda_{v}-\mu_{v})F_{vh}^{*}}{\Lambda_{v}(\theta +m\sigma_{s}+\mu_{v})(F_{vh}^{*}+m\sigma_{s}+\mu_{v})},\;E_{SR}^{*}=\frac{2pqK(\Lambda_{v}-\mu_{v})F_{vh}^{*}}{\Lambda_{v}(\theta +m\sigma_{r}+\mu_{v})(F_{vh}+m\sigma_{r}+\mu_{v})},\\ E_{RR}^{*}= & \frac{q^{2}K(\Lambda_{v}-\mu_{v})F_{vh}^{*}}{\Lambda_{v}(\theta +m\sigma_{r}+\mu_{v})(F_{vh}^{*}n+m\sigma_{r}+\mu_{v})},\;I_{SS}^{*}=\frac{p^{2}\theta K(\Lambda_{v}-\mu_{v})F_{vh}^{*}}{\Lambda_{v}A_{s}},\\ I_{SR}^{*}= & \frac{2pq\theta K(\Lambda_{v}-\mu_{v})F_{vh}^{*}}{\Lambda_{v}A_{r}},\;I_{RR}^{*}=\frac{q^{2}\theta K(\Lambda_{v}-\mu_{v})F_{vh}^{*}}{\Lambda_{v}A_{r}}, \end{array} \end{array}$$ (4)

where $\mu_{v}=\mu_{vs}=\mu_{vr}$, $A_{s}=(m\sigma_{s}+\mu_{v})(\theta +m\sigma_{s}+\mu_{v})(F_{vh}^{*}+m\sigma_{s}+\mu_{v})$, $A_{r}=(m\sigma_{r}+\mu_{v})(\theta +m\sigma_{r}+\mu_{v})(F_{vh}^{*}+m\sigma_{r}+\mu_{v})$, and:


$$\begin{array}{c} {F^{*}}_{hv}=\frac{\theta \tau_{h}K(\Lambda_{v}-\mu_{v}){F^{*}}_{vh}}{\Lambda_{v}}\left[\frac{p^{2}}{A_{s}}+\frac{q(2p+q)}{A_{r}}\right](m\beta_{bloc}\rho_{in}+(1-m)\rho_{out}), \end{array}$$ (5)

$$\begin{array}{c} F_{vh}^{*}=\frac{\tau_{v}\Lambda_{h}F_{hv}^{*}(\vartheta +\mu_{h})(m\beta_{bloc}\rho_{in}+(1-m)\rho_{out})}{\mu_{h}\varphi F_{hv}^{*}+(\vartheta +\mu_{h})[(\mu_{h}+\gamma )F_{hv}^{*}+(\vartheta +\gamma +\mu_{h})\mu_{h}]}. \end{array}$$ (6)

Substituting equation 6 into the human force of infection equation 5, yields the following:


$$\begin{array}{c} {F^{*}}_{hv}[aH_{s}H_{r}{F^{*}}_{hv}^{2}+D_{1}{F^{*}}_{hv}+D_{2}]=0, \end{array}$$ (7)

where $D_{1}=aB[f_{r}H_{s}+f_{s}H_{r}]-[C_{s}BH_{r}+C_{r}H_{s}]$, $D_{2}=Bf_{s}f_{r}(aB-\frac{C_{s}}{f_{s}}-\frac{C_{r}}{f_{r}})$, $a=\frac{\Lambda_{v}}{\theta \tau_{h}\tau_{v}\Lambda_{h}K(\Lambda_{v}-\mu_{v})(\vartheta +\mu_{h})(m\beta_{bloc}\rho_{in}+(1-m)\rho_{out}{)}^{2}}$, $H_{s}=\tau_{v}\Lambda_{h}(\vartheta +\mu_{h})(m\beta_{bloc}\rho_{in}+(1-m)\rho_{out})+[\varphi \mu_{h}+(\vartheta +\mu_{h})(\gamma +\mu_{h})](m\sigma_{s}+\mu_{v})$, $H_{r}=\tau_{v}\Lambda_{h}(\vartheta +\mu_{h})(m\beta_{bloc}\rho_{in}+(1-m)\rho_{out})+[\varphi \mu_{h}+(\vartheta +\mu_{h})(\gamma +\mu_{h})](m\sigma_{r}+\mu_{v})$, $C_{s}=\frac{p^{2}}{\left(m\sigma_{s}+\mu_{v})(\theta +m\sigma_{s}+\mu_{v}\right)}$, $C_{r}=\frac{q(2p+q)}{\left(m\sigma_{r}+\mu_{v})(\theta +m\sigma_{r}+\mu_{v}\right)}$ and $B=(\vartheta +\mu_{h})(\vartheta +\gamma +\mu_{h})\mu_{h}$. From equation 4, if ${F^{*}}_{hv},{F^{*}}_{vh},\sigma_{s},\sigma_{r},\theta ,\vartheta ,\varphi =0$ the equilibrium point obtained represents the MFE steady state. For malaria endemic equilibrium state, ${F^{*}}_{hv},{F^{*}}_{vh},\sigma_{s},\sigma_{r},\theta ,\vartheta ,\varphi >0$. Therefore, the endemic equilibrium point exists if;


$$\begin{array}{c} {F^{*}}_{hv}=\frac{-D_{1}\pm \sqrt{D_{1}^{2}-4aH_{s}H_{r}D_{2}}}{2aH_{s}H_{r}}, \end{array}$$ (8)

for $D_{1}^{2}\geq 4aH_{s}H_{r}D_{2}$. This implies that ${F^{*}}_{hv}$ can take on two values which demonstrates a possibility of existence of two endemic equilibrium points.

## 4. Results of the study

This section investigated four LLIN utilization scenarios and their effect on malaria transmission and resistance evolution over three successive LLIN distribution campaigns. The baseline scenario considered no bed net deployment. In all three intervention scenarios, each campaign starts with an initial bed net utilization level of 81% which represent bed net ownership and correct usage. Following LLIN deployment, this utilization was considered to decay exponentially over time due to physical bed net attrition and reduced user adherence, with a minimum threshold of 1%. The decline in LLIN utilization was modelled using an exponential decay function such that:


$$\begin{array}{c} {\text{LLIN}}_{cov}(t)={\text{LLIN}}_{cov}(0)\exp(-\delta_{cov}t), \end{array}$$ (9)

where ${\text{LLIN}}_{cov}(0)=0.81$ is the initial bed net utilization and $\delta_{cov}$ is the rate of decay in effective bed net coverage. For each utilization scenario, the decay rate $\delta_{cov}$ is given as:


$$\begin{array}{c} \delta_{cov}=\frac{\ln({\text{LLIN}}_{cov}(0)/{\text{LLIN}}_{\min})}{T}, \end{array}$$ (10)

where ${\text{LLIN}}_{\min}=0.01$ represents the minimum threshold imposed to reflect a residual level of bed net presence within the population. In a similar way, the LLIN blocking effect LLIN_bloc_ and pyrethroid killing effect LLIN_eff_ were considered to decay exponentially at rates $\delta_{bloc}$ and $\delta_{eff}$ respectively. Additionally, the PBO synergist with an initial effectiveness of $PBO_{eff}=0.68$ [43,44], decayed exponentially at the rate $\delta_{pbo}$ such that $\delta_{pbo}=0.0002324$ and $\delta_{pbo}=0.001156$ for year one and two of the bed net campaign respectively [26]. All parameter values used in numerical simulations (using Python 3.10.11 software package) are included in Table 1.

**Table 1:** Model parameters, definitions, units, and sources for the genotype-specific malaria transmission model 2 with LLIN interventions.

Parameter	Description	Unit	Value	Reference
$\tau_{h}$	Probability of an infectious mosquito transmitting		0.092	[56]
	malaria to a susceptible human			
$\tau_{v}$	Probability of an infectious human transmitting		0.167	[57,58]
	malaria parasites to a susceptible mosquito			
ϑ	Rate at which individuals that have recovered from	Per day	1/(0.5x365)	[59]
	malaria infection become susceptible			
$\rho_{in}$	Indoor biting rate	Bites per person	17.3	[60]
		per day		
$\rho_{out}$	Outdoor biting rate	Bites per person	2.3	[60]
		per day		
$\Lambda_{h}$	Human Population growth rate	per day	0.00009	[61]
$\Lambda_{v}$	Pyrethroid sensitive mosquito recruitment rate	per day	0.25	[62,63]
$\Lambda_{vr}$	Pyrethroid resistant mosquito recruitment rate	per day	0.25×(1−c)	[62,63]
$\mu_{h}$	Human natural death rate	Per day	1/(68.2 x 365)	[61]
φ	Recovery rate of infectious humans from malaria infection	Per day	1/ 14	[64]
θ	Rate at which latently infected mosquito vectors	Per day	1/10	[65]
	become infectious			
γ	Rate at which humans die from malaria	Per day	0.00138	[66]
$\mu_{vs}$	Pyrethroid sensitive mosquito natural death rate	Per day	1 / 14	[67]
$\mu_{vr}$	Pyrethroid resistant mosquito natural death rate	Per day	w× 1 / 14	[52,53]
${\text{LLIN}}_{cov}$	LLIN initial bed net coverage		0.81	[26]
${\text{LLIN}}_{eff}$	LLIN initial killing effectiveness in sensitive mosquitoes		0.98	[68,69]
${\text{LLIN}}_{effr}$	LLIN initial killing effectiveness in resistant vectors		0.55	[68]
${\text{LLIN}}_{bloc}$	LLIN initial blocking effect		0.85	[26,70]
${\text{PBO}}_{eff}$	PBO initial synergistic effect		0.68	[43,44]
$\delta_{eff}$	Rate of decay of pyrethroid killing effectiveness in	Per day	0.0031404	Estimated
	sensitive vectors			
$\delta_{effr}$	Rate of decay of pyrethroid killing effectiveness in	Per day	0.0027447	Estimated
	resistant vectors			
$\delta_{bloc}$	Rate at which LLIN blocking effect decays	Per day	0.00164	[26]
$\delta_{cov}$	Standard Rate at which LLIN coverage decays	Per day	0.0030099	[27]
$\delta_{pbo}$	Rate at which PBO effect decays	Per day	0.0002324 and	[26]
			0.001156	
*m*	Initial proportion of mosquitoes that feed indoors		0.6	[71]
*f*	Proportion of indoor bitters that make physical		0.70	Assumed
	contact with bed nets			
c	Modification parameter for fitness cost in mosquito		0.1	[72]
	recruitment			
w	Modification parameter for fitness cost in mosquito		1.15	[72]
	adult lifespan			

The standard utilization scenario assumed *T* = 4 years, a midpoint between the expected bed net utilization and durability range of three to five years [45]. Other utilization scenarios explored accelerated decay in bed net effective usage corresponding to *T* = 3, 2 and 1 year(s), representing an increasing reduction in bed net utilization. Across all scenarios, LLIN campaigns were implemented at a three year intervals, which is in line with WHO recommendations [46], with each new LLIN deployment restoring bed net utilization to 81%. Across the three successive campaigns, pyrethroid-only nets were deployed in the first two rounds, while pyrethroid-PBO nets were introduced in the third campaign. These utilization scenarios were compared in terms of malaria transmission, vector suppression and selection pressure for insecticide resistance.

At the start of the simulation, 60% of the mosquito population blood feed indoors and of this, 70% makes actual contact with LLINs. The initial susceptible $S_{h}(0)$, infectious $I_{h}(0)$ and recovered $R_{h}(0)$ human population was set at 1.5 million, 100 and zero people respectively. A carrying capacity of 6 million vectors was set with initial mosquito numbers of $S_{SS}(0)=7500$, $E_{SS}(0)=500$, $I_{SS}(0)=100$, $S_{sr}(0)=1150$, $E_{sr}(0)=600$, $I_{sr}(0)=50$, $S_{rr}(0)=75$, $E_{rr}(0)=20$ and $I_{rr}(0)=5$. Additionally, to track mosquito dynamics across years, the rate of new mosquito recruitment $\Lambda_{v}(t)$ was modified to follow a seasonal pattern such that;


$$\begin{array}{c} \Lambda_{v}(t)=\Lambda_{v}(0)[1+A_{1}\sin(\frac{2\pi t}{T}+\phi_{1})+A_{2}\sin(\frac{4\pi t}{T}+\phi_{2})], \end{array}$$ (11)

where $\Lambda_{v}(0)$ is the baseline mosquito recruitment rate, *A*_1_ and *A*_2_ are amplitudes of seasonal cycles [47,48]. Parameters *A*_1_ and *A*_2_ correspond to the primary annual cycle associated with the first seasonal peak and the secondary semi-annual cycle responsible for the second seasonal peak, respectively. Phase shift parameters $\phi_{1}$ and $\phi_{2}$ control the timing at which rainfall peaks occur within the year, such that $\phi_{1}=\frac{2\times 120\pi }{T}$ and $\phi_{2}=\frac{4\times 300\pi }{T}$ for *T* = 365 days. Each campaign deployed LLINs 30 days into the year, at the start of the first seasonal peak for maximum impact [34]. However, the bed net distribution is not an instantaneous activity, often spanning between weeks to months. Therefore, a sigmoid function was used to model the increase in the level of bed net utilization from zero to a maximum target of 81% [26], as follows:


$$\begin{array}{c} f(x)=\frac{L}{1+e^{-k(x-x_{0})}}, \end{array}$$

where *L* is the target LLIN utilization (*L* = 0.81), *x* is time in years, *x*_0_ is the time taken for coverage to reach 50% of the target effective coverage, and *k* represents the rate of increase in utilization level.

Fitness costs in pyrethroid-resistant malaria vectors impact their survival, gonotrophic cycle duration, fecundity, and their adult lifespan. The effect of pyrethroid resistance on mosquito adult longevity remains inconclusive. While some studies have reported that resistance, particularly in *Anopheles gambiae*, increases adult life span among resistant mosquitoes [49–51] others have demonstrated a reduction in adult longevity [52–54]. In principle, resistance mechanisms such as over-expression of detoxification enzymes or target site mutation require substantial resources, and these can be diverted from other life-history traits like fecundity and longevity [55]. Therefore, for numerical simulations, the model system 2 was modified to have fitness costs (reduced mosquito fecundity and adult longevity for resistant vectors), time dependent LLIN coverage, killing effectiveness and blocking effects, while incorporating seasonal mosquito recruitment.

### 4.1. Sensitivity analysis of the malaria transmission reproduction number under LLIN intervention

**Fig 2:**
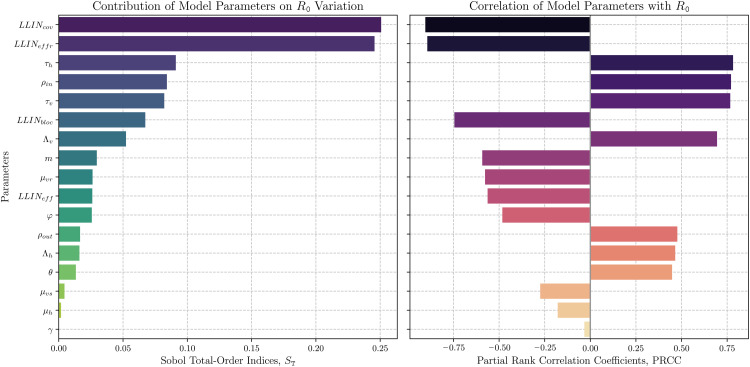
Sensitivity analysis of malaria transmission reproduction number during bed net usage $R_{0}^{L}$ to changes in model parameters.

To identify which model parameters have the greatest influence on malaria transmission under bed net usage, a global sensitivity analysis of the reproduction number $R_{0}^{L}$ – represented by equation 3– was conducted using Sobol and Partial Rank Correlation Coefficient (PRCC) techniques. Sobol indices quantify the proportion of variance in $R_{0}^{L}$ resulting from each parameter, while PRCC analysis measures monotonic relationships between input parameters and $R_{0}^{L}$. The PRCC values were computed by rank-transforming inputs and outputs, removing effects of other parameters via regression, and correlating the resulting residuals using Spearman’s method on the Sobol-generated sample set. Together, these methods identify the parameters most strongly influencing malaria transmission under LLIN deployment. Using the Sobol sampling method (via the SALib Python package), a total of 131072 different combinations of parameter values was generated in computing $R_{0}^{L}$ and the results were summarized in Fig 2.

Fig 2 shows that the level of bed net utilization (proportion of people sleeping under bed nets) ${\text{LLIN}}_{cov}$ and the effectiveness of these bed nets in killing pyrethroid resistant mosquitoes ${\text{LLIN}}_{effr}$, have the strongest influence on malaria transmission. This result demonstrates that ${\text{LLIN}}_{cov}$ and ${\text{LLIN}}_{effr}$ act directly on the human–vector contact and mosquito survival components of the transmission process, which are the primary pathways driving malaria spread in the model. This is consistent with evidence from field trials and durability studies showing that sustained LLIN utilization [73] and maintained bioefficacy against resistant mosquitoes [74] are critical for reducing malaria transmission. The increase in either LLIN utilization ${\text{LLIN}}_{cov}$ or bed net effectiveness against resistant vectors ${\text{LLIN}}_{effr}$ was associated with a reduction in $R_{0}^{L}$ showing a strong suppressive effect on malaria transmission. This transmission appears to rise with increase in mosquito recruitment, indoor and out door biting rates, rate at which latently infected vectors become infectious (θ), or probability of parasite transfer between human and malaria vectors. However, the simulation shows that increase in θ has the least effect on malaria transmission among all model parameters in $R_{0}^{L}$.

In contrast, the model shows that reductions in bed net effectiveness against both susceptible and resistant vectors, as well as lower effective coverage, lead to increases in $R_{0}^{L}$, thereby weakening malaria control. An increase in the rate of malaria induced death rate γ offers the least effect, among all model parameters, in reducing $R_{0}^{L}$. These results demonstrate that LLIN coverage (${\text{LLIN}}_{cov}$) and effectiveness against resistant mosquitoes (${\text{LLIN}}_{effr}$) are the dominant drivers of malaria transmission under LLIN campaigns. Therefore, for model system 2, it is noted that malaria control is most effective when bed net utilization is high and these nets remain potent against malaria vectors especially resistant mosquitoes throughout the campaign period.

### 4.2. Effect of decreasing LLIN utilization on malaria transmission

To investigate the effect of decreasing bed net usage on malaria transmission, three bed net campaigns were simulated under decreasing LLIN utilization durations. The effectiveness of each intervention was quantified as the proportion of susceptible humans protected from malaria infection under a given campaign period.

**Fig 3:**
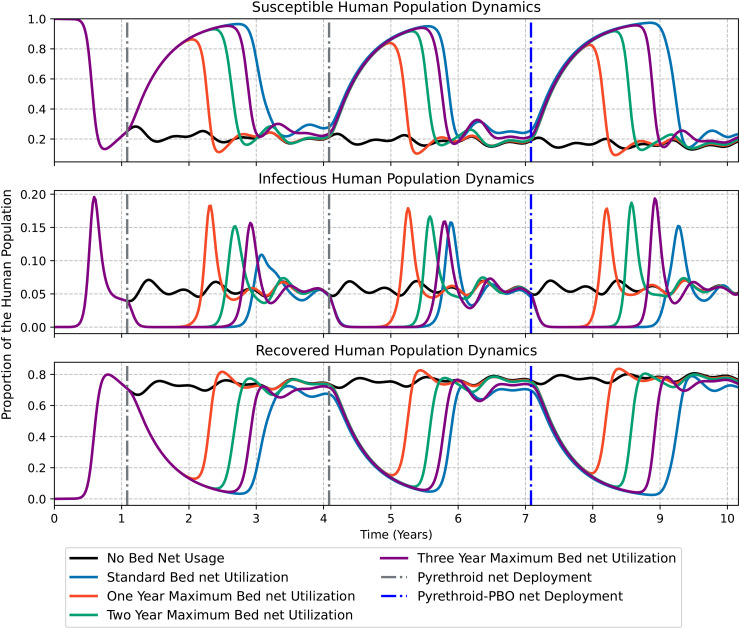
Human malaria dynamics, classified into susceptible (top panel), infectious (middle panel), and recovered (bottom panel), under varying LLIN utilization scenarios across three bed net campaigns, with differing rates of decay in bed net utilization from an initial 81% to 1%.

Without LLIN usage, the proportion of susceptible humans fluctuated between 0.18 and 0.30, while following the seasonal annual dynamics, Fig 3. The proportion of infectious humans remained at an average of 5% of the total population over the nine-year simulation period. Following the deployment of pyrethroid-only nets, this proportion declined to below 1%, before gradually recovering towards baseline levels as the intervention effectiveness waned off. Correspondingly, the recovered human population decreased during periods of reduced malaria transmission but increased as this infection resurged.

**Fig 4:**
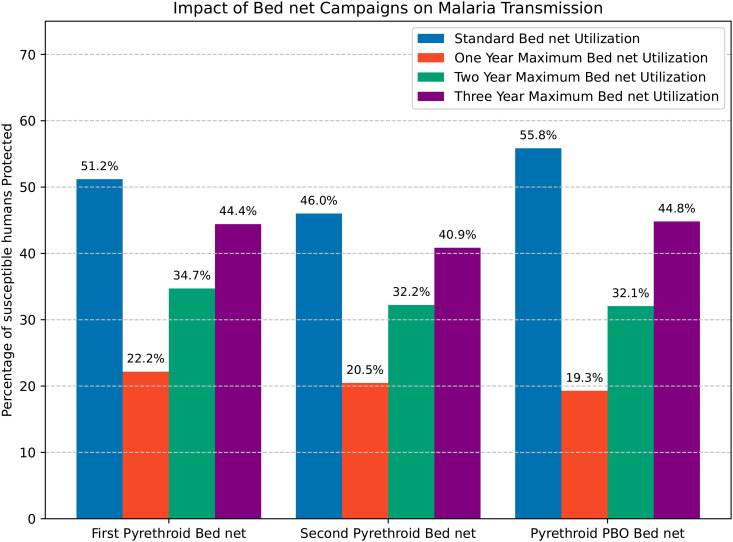
Model estimated percentages of susceptible humans protected from malaria infection over two successive pyrethroid-only and one pyrethroid-PBO bed net campaigns, under varying LLIN utilization scenarios with initial bed net utilization set at 81% for each deployment.

Under standard utilization, bed nets maintained the suppression of infectious humans for about 2.5 years following each of the three deployments. This scenario showed the highest level of human protection from malaria infection, preventing up to 51.2%, 46.0%, and 55.8% of the potential infections during the first, second and third campaigns respectively. To quantify the impact of LLIN campaigns across the four utilization scenarios, the percentage of susceptible humans protected from malaria infection by bed nets is plotted in Fig 4. Across all three bed net campaigns, the LLIN effectiveness declined with decreasing durations of utilization (Fig 4). During the first campaign using pyrethroid-only nets, the proportion susceptible humans protected from malaria infection decreased from 51.2% to 44.4%, 34.7% and 22.2% under standard usage, three-, two-, and one-year utilization scenarios respectively. The relative advantage of pyrethroid-PBO nets over pyrethroid-only nets decreased under lower LLIN utilization, with no observable difference in protection registered under the one- and two-year utilization scenarios.

### 4.3. Effect of decreasing LLIN utilization on mosquito population Dynamics

This examined the effect of sequential LLIN campaigns on the epidemiological and total mosquito dynamics within seasonal settings.

#### 4.3.1. LLIN utilization and total mosquito population dynamics.

In the absence of interventions, the mosquito population exhibited a bimodal seasonal pattern, with a primary mosquito population peak occurring in April and the a secondary, smaller peak in October [75,76]. LLIN deployment resulted in substantial reductions in the total mosquito population across all utilization scenarios.

**Fig 5:**
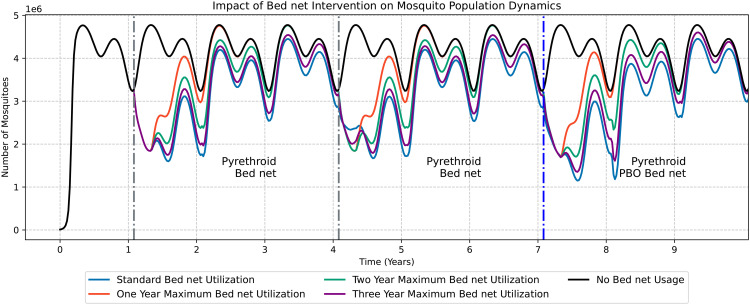
Total mosquito population dynamics across three successive bed net campaigns, under varying LLIN utilization scenarios. The grey dashed lines indicate pyrethroid-only bed nets deployment while the dashed blue line indicates pyrethroid–PBO nets deployment.

Under standard utilization (Fig 5), mosquito populations declined to below two million shortly after deployment, but these gradually recover towards baseline as intervention effectiveness decreased. Subsequent campaigns produced similar patterns, although the magnitude of suppression declined under shorter LLIN utilization scenarios. The third campaign achieved the greatest reduction in mosquito population, with vector abundance decreasing to approximately one million within the first year before gradually rebounding during the course of this campaign period. Particularly, PBO nets deployed under standard utilization settings, was observed to suppress malaria vectors by about 72%, but pyrethroid-only nets had less effect on the mosquito population.

The declining bed net utilization levels substantially compromised the effectiveness of LLINs in suppressing malaria vectors, with the one-year utilization settings loosing mosquito killing effect in about 13 months after deployment. However, all four utilizations scenarios demonstrated the initial suppression effect on the mosquito population.

#### 4.3.2. LLIN utilization and mosquito epidemiological dynamics.

**Fig 6:**
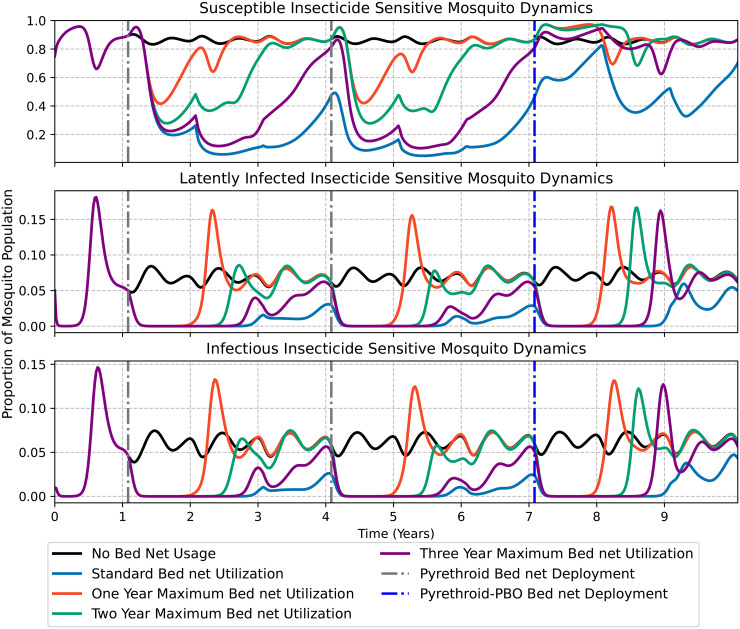
Temporal dynamics of pyrethroid-sensitive mosquito populations, classified in terms of susceptible (top panel), latently infected (middle panel), and infectious (bottom panel), under varying LLIN utilization periods and successive bed net deployment campaigns.

Fig 6 illustrates the impact of LLIN campaigns on pyrethroid-sensitive malaria vectors. Generally, LLIN deployments cause sudden reductions in the mosquito population, especially pyrethroid-sensitive vectors, but their numbers recover as the bed net effectiveness decays. Susceptible sensitive vectors maintained maximum levels when no bed net campaigns were deployed, stabilizing above 80% of the total mosquito population, while latently infected and infectious mosquitoes stabilized between 5% to 7.5%.

Pyrethroid-only bed net campaigns suppressed sensitive vectors to nearly zero for almost two years upon deployment under standard utilization settings, but the PBO nets showed less impact on susceptible pyrethroid-sensitive vectors. All campaigns demonstrated high effectiveness in suppressing both latently infected and infectious malaria vectors, and PBO nets reduced these mosquitoes to near zero for the greater part of the campaign period. The one-year utilization scenario performed worst in reducing sensitive vectors, with the susceptible mosquitoes recovering towards baseline just after five months of deployment, and infected vectors recovering after one year into the campaign. Therefore, shorter utilization durations resulted in faster recovery of both susceptible and infected mosquito classes towards baseline.

**Fig 7:**
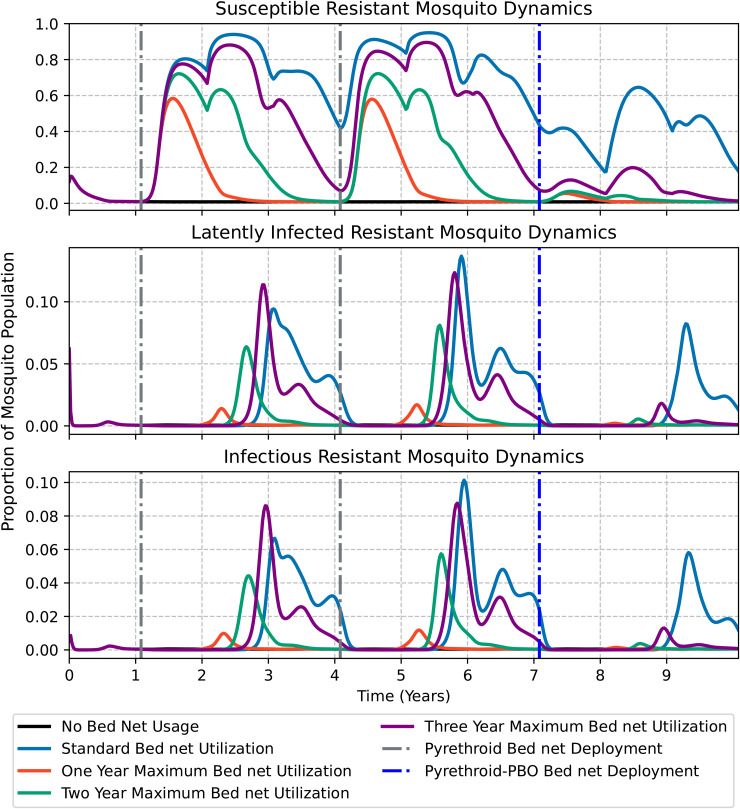
Temporal dynamics of pyrethroid-resistant mosquito populations, classified in terms of susceptible (top panel), latently infected (middle panel), and infectious (bottom panel), under varying LLIN utilization periods and successive bed net deployment campaigns.

Conversely, the proportion of pyrethroid-resistant mosquitoes increased following the deployment of pyrethroid-only nets (Fig 7)). Susceptible pyrethroid-resistant vectors increased at a faster rate than infected mosquitoes, reaching approximately 90% of the population within two years under standard utilization, before declining towards the end of the campaign period. Shorter utilization scenarios produced lower peaks and more rapid declines in resistant vector proportions. Therefore, across all four utilization settings, standard usage sustained resistant vectors for longer periods due to its higher selection for resistance. However, PBO nets displayed better suppression of resistant vectors across the four utilization scenarios, although this effort reduced as the overall bed net killing effectiveness waned.

For pyrethroid-only campaigns, the proportion of infected resistant mosquitoes remained minimal during the initial phase of each campaign period, across the four utilization scenarios. This is because mosquitoes first survive a blood feeding attempt before becoming infected with malaria parasites. Immediately after LLIN deployment, strong blocking and killing effects substantially limited human-mosquito contact [77], while the number of infectious humans available for malaria transmission was low at this time [78]. However, as LLIN effectiveness exponentially declined over time, more mosquitoes successfully blood fed. This effectiveness continued to decay further, and more sensitive mosquitoes survived the bed net intervention, causing an increase in their proportion, consequently reducing the fraction of resistant vectors.

### 4.4. Effect of bed net utilization and fitness cost on the evolution of pyrethroid resistance

**Fig 8:**
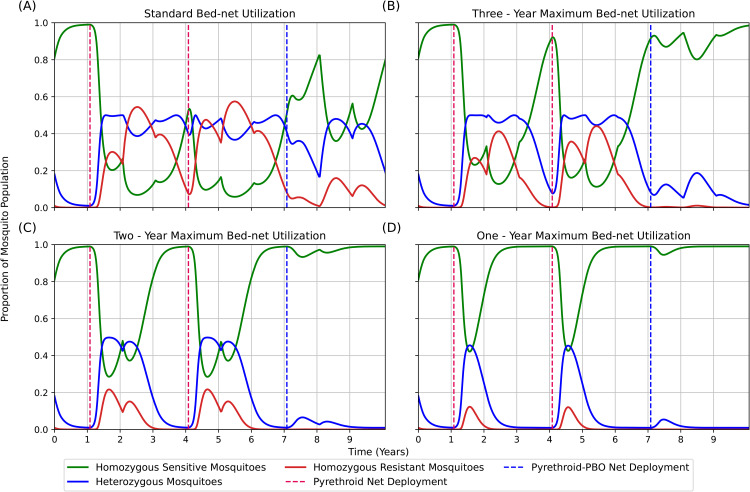
Panels A – D illustrate the changing proportions of three mosquito genotypes – homozygous sensitive (green), heterozygous (blue) and homozygous resistant (red) – over time in response to successive LLIN deployment campaigns. The red vertical dashed lines indicate the deployment of pyrethroid-only nets, while the blue vertical dashed lines indicate deployment of pyrethroid-PBO nets.

**Fig 9:**
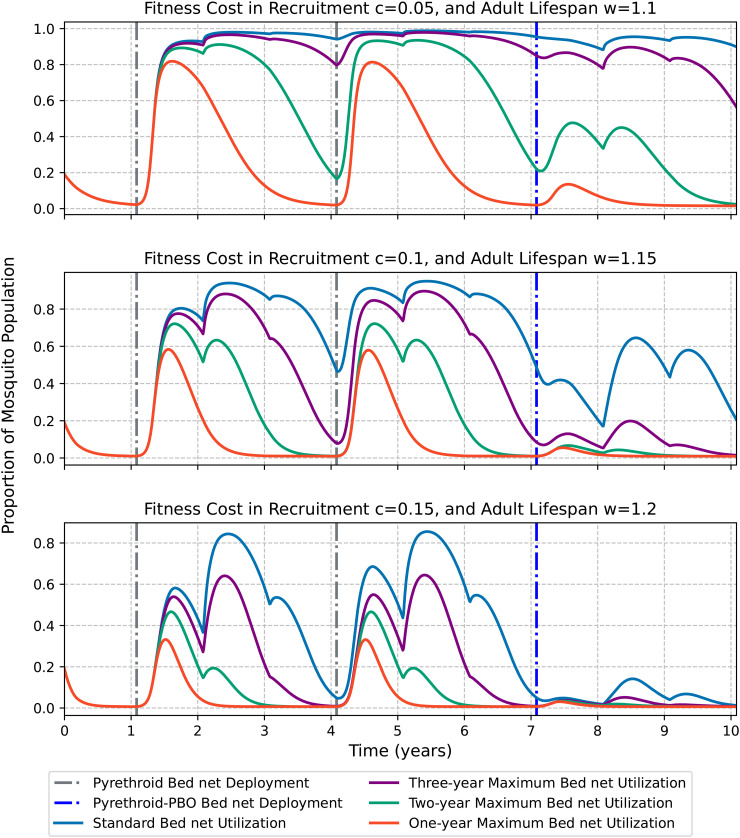
Evolution of pyrethroid resistance within the mosquito population across three successive bed net campaigns, under increasing fitness costs and varying LLIN utilization periods.

The effect of bed net utilization and variations in fitness cost on the dynamics of pyrethroid resistance within the mosquito vectors, across the four bed net usage scenarios, was investigated in Fig 8 and Fig 9 respectively.

#### 4.4.1. Effect of bed net utilization on the evolution of pyrethroid resistance.

Fig 8 shows the variations in sensitive, heterozygous and homozygous resistant genotype frequencies under three bed net campaigns, across different bed net utilization settings.

Under standard utilization (panel A of Fig 8), the homozygous sensitive genotypes rapidly declined during the pyrethroid-only campaigns, reaching about 10% within 16 months, demonstrating a strong selection pressure exerted by the sustained insecticide exposure. Heterozygous and homozygous resistant genotype frequency increased correspondingly, with heterozygotes rising to approximately 50% and homozygous resistant vectors to 55% in sixteen months. As the bed net utilization and killing effectiveness declined towards the end of the campaign period, selection pressure reduced leading to the reduction in the percentage of homozygous resistant mosquitoes to about 10%, but heterozygous vectors persisted due to their comparative fitness cost advantage under reduced selection pressure. The deployment of pyrethroid-PBO nets in the third campaign reduced resistant genotype frequencies, with heterozygous and homozygous resistant proportions declining to approximately 20% and 2% respectively.

Under shorter utilization scenarios (for example, in panels C and D of Fig 8), increases in resistant genotype frequencies upon deployment of pyrethroid-only nets were smaller and less sustained, with resistant genotypes declining faster to near zero by the end of each campaign period. The introduction of pyrethroid-PBO nets in the third campaign further suppressed resistant genotypes, giving advantage to the pyrethroid sensitive vectors to dominate the population.

#### 4.4.2. Effect of fitness cost on the evolution of pyrethroid resistance.

In this subsection, different fitness cost values were used to understand their effect on the evolution of pyrethroid resistance among mosquito populations. Two kinds of fitness costs were considered, with one affecting mosquito recruitment (c) and the other reducing adult lifespan (w) of heterozygous and homozygous resistant vectors.

Fig 9 demonstrated that evolution of pyrethroid resistance among the mosquito population reduces with increase fitness costs. With a fitness cost of 5% reduction in resistant vector recruitment, and 10% decrease in adult lifespan of resistant mosquitoes, a higher selection force was observed. Under these conditions, the standard utilization scenario led to near fixation of resistant genotypes by the end of the first and second pyrethroid-only campaigns. The PBO net deployment during the third campaign substantially reduced the percentage of resistant vectors for one and two year(s) utilization scenarios to 2%. However, longer usage periods showed less effect, reducing the percentage of resistant vectors to 95% and 58% for the standard and three-year utilization scenarios, respectively.

When these fitness costs in recruitment and adult lifespan increased to 10% and 15% respectively, resistance emerged at a reduced rate reaching a peak of 94% in sixteen months, but reducing to 48% by the end of the first campaign. During the second campaign, a similar trend was observed. However, the deployment of pyrethroid-PBO nets led to a substantial reduction in resistance level, peaking at 62% before gradually declining to 20% at the end of the campaign period. The selection pressure for resistance from LLINs reduced as utilization periods decreased, with the one and two-year usage scenarios showing no total increase in resistance across pyrethroid-only campaigns.

Higher fitness costs of 15% and 20% in mosquito recruitment and adult lifespan, respectively, substantially reduced the selection for resistance within and across pyrethroid-only campaigns. Under standard utilization, the percentage of resistant vectors increased to about 83% of the mosquito population after sixteen months from deployment, but this reduced to less than 10% by the end of this campaign. Shorter utilization periods showed lower resistance peaks and overall selection force, thus registering negligible resistant percentages across pyrethroid-only campaigns. At these low levels of resistance, application of PBO nets suppressed the within-campaign resistance peaks and generally maintained the minimum proportions of resistant vectors. Therefore, fixation of resistance genotype was less likely to occur when fitness costs were at least 15% and 20% for vector recruitment and adult lifespan, respectively. Under such conditions, a single pyrethroid-PBO net campaign sufficiently suppressed resistant vectors to negligible proportions.

## 5. Discussion

This study formulated a deterministic mathematical model incorporating genotype-structured mosquito dynamics and human-vector interactions to evaluate the impact of LLIN utilization on malaria transmission and resistance evolution. The expressions for malaria transmission reproduction numbers $R_{0}$ and $R_{0}^{L}$ with and without bed net usage respectively were computed. Sensitivity analysis on $R_{0}^{L}$ identified LLIN utilization and effectiveness of against resistant vectors as the dominant drivers of reduction in malaria transmission, indicating that sustained coverage and insecticidal performance are critical for intervention success.

The results showed that, despite an initial LLIN coverage of 81%, protection against malaria infection by both pyrethroid-only and pyrethroid-PBO nets was not sustained over the three year campaign period and this worsened with lower bed net utilization levels. This is because, as utilization decreased, both the frequency and duration of human–mosquito contact increase, leading to a rapid erosion of epidemiological impact. Pyrethroid-PBO nets demonstrated higher effectiveness, particularly under standard utilization. These nets are designed to kill both pyrethroid sensitive and resistant malaria vectors [79], hence displaying higher effectiveness as a vector control tool. However, the advantage of pyrethroid-PBO nets over pyrethroid-only nets diminished with lower bed net utilization scenarios. This highlights the importance of bed net utilization on the overall effectiveness of LLIN campaigns, and how low bed net usage could compromise the effectiveness of PBO nets (see also [22,23,80]). Consequently, effectiveness of pyrethroid-only and pyrethroid-PBO nets, measured in terms of vector suppression, was undermined by the decline in effective coverage. This is because the entomological impact of these nets was substantially negated along the LLIN campaign period as more humans reduced or stopped using their bed net usage. This is consistent with the study by Okiring and others [80], which showed that LLIN usage decline within 12–18 months of the campaign, undermining their long-term impact.

The study also demonstrates that under standard utilization, successive pyrethroid-only campaigns amplify resistant mosquito genotypes, especially heterozygotes, but the deployment of PBO nets reverses this trend. Consistent with prior results [81–83], pyrethroid-PBO nets offer a critical benefit in reversing insecticide resistance within the malaria vector population, although their effectiveness depends on the level of bed net utilization. Further analysis showed that pyrethroid-PBO nets are more effective in reducing resistant vector proportions, when resistance to pyrethroid insecticides carries a higher fitness cost, for all four utilization scenarios. This is because higher fitness costs slow the evolution of pyrethroid resistance due to lower vector recruitment rates and survival advantage, a result in line with [83]. However, reduction in LLIN utilization led to a decrease in selection pressure against mosquitoes consequently slowing the evolution of pyrethroid resistance. This reduced utilization may lead to increased human exposure to mosquito bites and a higher malaria risk [84], making it epidemiologically undesirable for malaria control.

Heterogeneity and localized transmission hotspots play an important role in malaria spread [85], hence the assumption that there is homogeneous mixing between human and mosquito populations limits the findings of this study. Public health bed net campaigns face delays, causing irregular deployment of nets [34], which impacts malaria transmission. Therefore the assumption of regular replacement of bed nets after three years may not practically hold, especially in African settings. Pyrethroid resistance under field conditions is polygenic in nature [86] and fitness costs incurred by resistant vectors vary with seasonal conditions, genetic background, and differences in mosquito adaptive responses [87]. This study used a genetic structure represented by two alleles with fixed fitness costs, a limitation for its findings. Additionally, the study assumes that LLIN interventions do not affect mosquito behaviour, all humans have equal risk of malaria infection and there is no adaptive immunity among the human population. However, evidence shows that LLIN campaigns affects mosquito behaviour [88], age affects the risk of malaria infection [89] and adaptive immunity contributes to the overall mosquito dynamics [90].

## 6. Conclusion

The study demonstrates that bed net utilization is a key determinant of effectiveness of LLIN campaigns and evolution of pyrethroid resistance among malaria vectors. Despite of a high initial bed net coverage, declining utilization over the three-year campaign period substantially reduces the epidemiological and entomological effectiveness of LLIN campaigns, thus threatening sustainable protection against malaria.

It further shows that pyrethroid-PBO nets provided greater vector suppression and protection against malaria infection compared to pyrethroid-only nets under standard utilization, but this advantage diminishes with as bed net utilization declines. Successive deployment of pyrethroid-only campaigns accelerate resistance evolution, while pyrethroid-PBO nets counteract this trend especially when pyrethroid resistance attracts higher fitness costs. Although lower bed net utilization scenarios led to reduced evolution of pyrethroid-resistance, they are not epidemiologically viable for malaria control.

Therefore, maintaining high bed net utilization throughout the campaign period is essential for maximizing the epidemiological effectiveness of LLIN campaigns and sustaining malaria control. However, to limit the selection pressure associated with prolonged use of pyrethroid-only nets, rotation strategies incorporating pyrethroid-PBO LLINs should be adopted. Particularly, prioritizing pyrethroid-PBO nets at high utilization levels can enhance vector control while slowing the evolution of pyrethroid resistance.

## Supporting information

S1 Appendix Positivity, well-posedness and Boundedness. (PDF)

S2 AppendixMalaria free equilibrium (MFE) state and Basic Reproduction number $R_{0}$.(PDF)
