# Supplementary material for: Modelling the impact of mosquito bed net utilization on malaria transmission and evolution of pyrethroid resistance
Source: PLoS One. 2026 Jul 15;21(7):e0353301. doi: 10.1371/journal.pone.0353301 (PMC13372178; doi:10.1371/journal.pone.0353301)
Supplement: S1 Appendix — (PDF) [file pone.0353301.s001.pdf]

S1 Appendix. Positivity, well-posedness and Boundedness.

To fully represent the model system (2), the initial conditions of the form $\mathcal{D}(0) = (S_h(0), I_h(0), R_h(0), S_{SS}(0), E_{SS}(0), I_{SS}(0), S_{SR}(0), E_{SR}(0), I_{SR}(0), S_{RR}(0), E_{RR}(0), I_{RR}(0))$, where $N_h(0) = S_h(0) + I_h(0) + R_h(0)$ and $N_v = S_{SS}(0) + E_{SS}(0) + I_{SS} + S_{SR}(0) + E_{SR}(0) + I_{SR} + S_{RR}(0) + E_{RR}(0) + I_{RR}$ need to be within an epidemiologically feasible region. This is a region where state variable and parameter values make biological sense. For example, the state variables $S_h, I_h, R_h, S_{SS}, E_{SS}, I_{SS}, S_{SR}, E_{SR}, I_{SR}, S_{RR}, E_{RR}$ and I_{RR} and their corresponding initial conditions in $\mathcal{D}(0)$ are positive since they represent human and mosquito populations. Therefore, for initial conditions of the form $\mathcal{D}(0)$ in the epidemiologically feasible region,

$$\xi = \Big\{(S_h, I_h, R_h, S_{SS}, E_{SS}, I_{SS}, S_{SR}, E_{SR}, I_{SR}, S_{RR}, E_{RR}, I_{RR}) \in \mathbb{R}^{12}_+ : 0 < S_h + I_h + R_h < N_h(0)\exp^{(\Lambda_h - \mu_h)t}, 0 < S_{SS} + E_{SS} + I_{SS} + S_{SR} + E_{SR} + I_{SR} + S_{RR} + E_{RR} + I_{RR} < \frac{KN_v(0)}{N_v(0) + (K - N_v(0))\exp^{-\Lambda_v t}}\Big\}$$

The human population, from system (2), is such that $\frac{dN_h}{dt} = \Lambda_h - \mu_h N_h - \gamma I_h \leq \Lambda_h - \mu_h N_h$. This follows that $N_h(t) \leq \frac{\Lambda_h}{\mu_h} + \left(N_h(0) - \frac{\Lambda_h}{\mu_h}\right)\exp^{-\mu_h t}$ where $N_h(0)$ is the total number of people at time $t = 0$. Similarly, the total population of mosquitoes is given by the equation $\frac{dN_v}{dt} = \Lambda_v N_v\left(1 - \frac{N_v}{K}\right) - (m\sigma_s + \mu_{vs})N_{SS} - (m\sigma_r + \mu_{vr})(N_{SR} + N_{RR}) \leq \Lambda_v N_v\left(1 - \frac{N_v}{K}\right)$. It follows that $N_v(t) \leq \frac{KN_v(0)}{N_v(0) + (K - N_v(0))\exp^{-\lambda_v t}}$ for all $t \geq 0$, where $N_v(0)$ is the initial vector population. Therefore, as time $t \to \infty$;

1. The $\lim_{t\to\infty} N_h(t) \leq \frac{\Lambda_h}{\mu_h}$, thus the total human population converges to the steady state value $\frac{\Lambda_h}{\mu_h}$.
2. Similarly, $\lim_{t\to\infty} N_v(t) \leq \frac{KN_v(0)}{N_v(0)} = K$. Thus, the total mosquito population approaches the carrying capacity from below implying that this vector population will saturate around K in the long-run.

The epidemiologically feasible region $\xi = \Big\{(S_h, I_h, R_h, S_{SS}, E_{SS}, I_{SS}, S_{SR}, E_{SR}, I_{SR}, S_{RR}, E_{RR}, I_{RR}) \in \mathbb{R}^{12}_+ : 0 < S_h + I_h + R_h < \frac{\Lambda_h}{\mu_h} + \left(N_h(0) - \frac{\Lambda_h}{\mu_h}\right)\exp^{-\mu_h t}, 0 < S_{SS} + E_{SS} + I_{SS} + S_{SR} + E_{SR} + I_{SR} + S_{RR} + E_{RR} + I_{RR} < \frac{KN_v(0)}{N_v(0) + (K - N_v(0))\exp^{-\Lambda_v t}}\Big\}$ is positively invariant and attracting for all $t \geq 0$ with respect to the model system (2).

Lemma 1. *The solutions to system (2) are positive, indicating that region $\mathbb{R}^{12}_+$ is positively invariant.*

Proof. Suppose there exists t_1 such that $S_h(t_1) = 0$, $S_h(t_1)' \leq 0$ and $S_h(t) > 0$, $I_h(t) > 0$, $R_h(t) > 0$, $S_{SS}(t) > 0$, $E_{SS}(t) > 0$, $I_{SS}(t) > 0$, $S_{SR}(t) > 0$, $E_{SR}(t) > 0$, $I_{SR}(t) > 0$, $S_{RR}(t) > 0$, $E_{RR}(t) > 0$, $I_{RR}(t) > 0$, for $0 < t < t_1$. Thus,

$$S'_h(t_1) = \Lambda_h + \vartheta R_h - \frac{\tau_h(I_{SS}(t_1) + I_{SR}(t_1) + I_{RR}(t_1))}{N_h}\big(m\beta_{bloc}\rho_{in} + (1-m)\rho_{out}\big)S_h(t_1) - \mu_h S_h(t_1) > \tag{1}$$

which contradicts with $S_h(t_1)' \leq 0$. Therefore, t_1 does not exist. Further more, suppose t_2 exists such that $I_h(t_2) = 0$, $I_h(t_2)' \leq 0$ and $S_h(t) > 0$, $I_h(t) > 0$, $R_h(t) > 0$, $S_{SS}(t) > 0$, $E_{SS}(t) > 0$, $I_{SS}(t) > 0$, $S_{SR}(t) > 0$, $E_{SR}(t) > 0$, $I_{SR}(t) > 0$, $S_{RR}(t) > 0$, $E_{RR}(t) > 0$, $I_{RR}(t) > 0$, for $0 < t < t_2$. It follows that:

$$I_h'(t_2) = \frac{\tau_h(I_{SS}(t_2) + I_{SR}(t_2) + I_{RR}(t_2))S_h}{N_h}\big(m\beta_{bloc}\rho_{in}+(1-m)\rho_{out}\big)-\varphi I_h(t_2)-(\mu_h+\gamma)I_h(t_2) > \text{[illegible]} \quad (2)$$

which is in contradiction with $I_h(t_2)' \leq 0$. The same argument can be made for $R_h(t)$, $S_{SS}(t)$, $E_{SS}(t)$, $I_{SS}(t)$, $S_{SR}(t)$, $E_{SR}(t)$, $I_{SR}(t)$, $S_{RR}(t)$, $E_{RR}(t)$ and $I_{RR}(t)$. Therefore, starting with model system (2) with non-negative initial data in $\mathbb{R}_+^{12}$, the solution obtained is non-negative for all $t > 0$. □
