# Supplementary material for: Modelling the impact of mosquito bed net utilization on malaria transmission and evolution of pyrethroid resistance
Source: PLoS One. 2026 Jul 15;21(7):e0353301. doi: 10.1371/journal.pone.0353301 (PMC13372178; doi:10.1371/journal.pone.0353301)
Supplement: S2 Appendix — (PDF) [file pone.0353301.s002.pdf]

S2 Appendix. Malaria free equilibrium (MFE) state and Basic Reproduction number $\mathcal{R}_0$.

The MFE is obtained by setting the left hand side of model system (2) to zero and considering the infectious classes in both human and mosquito populations to be zero. At MFE, let $S_h(t) = S_h^*$, $I_h(t) = I_h^*$, $R_h(t) = R_h(t)^*$, $S_{SS}(t) = S_{SS}^*$, $E_{SS}(t) = E_{SS}^*$, $I_{SS}(t) = I_{SS}^*$, $S_{SR}(t) = S_{SR}^*$, $E_{SR}(t) = E_{SR}^*$, $I_{SR}(t) = I_{SR}^*$, $S_{RR}(t) = S_{RR}^*$, $E_{RR}(t) = E_{RR}^*$ and $I_{RR}(t) = I_{RR}^*$. Thus, the malaria free equilibrium during a bed net campaign ($\mu_{vr} \neq \mu_{vs}$), MFE state $E_0 = (S_h^*, I_h^*, R_h^*, S_{SS}^*, E_{SS}^*, I_{SS}^*, S_{SR}^*, E_{SR}^*, I_{SR}^*, S_{RR}^*, E_{RR}^*, I_{RR}^*)$ for model system (2), is given by $E_0 = \left(\frac{\Lambda_h}{\mu_h}, 0, 0, \frac{p^2\Lambda_v N_v^*(K - N_v^*)}{K(\mu_{vs} + m\sigma_s)}, 0, 0, \frac{2pq\Lambda_v N_v^*(K - N_v^*)}{K(\mu_{vr} + m\sigma_r)}, 0, 0, \frac{q^2\Lambda_v N_v^*(K - N_v^*)}{K(\mu_{vr} + m\sigma_s)}, 0, 0\right)$. Using the next generation matrix method, the basic reproduction number for system (2) is obtained by letting the rate of appearance of new malaria infection be given by $\mathcal{F}$ and the rate of transfer of infections through different classes $\mathcal{V}$ as follows;

$$F = \begin{bmatrix} \frac{\tau_h(I_{SS}+I_{SR}+I_{RR})S_h}{N_h}\left(m\beta_{bloc}\rho_{in} + (1-m)\rho_{out}\right) \\ \frac{\tau_v I_h S_{SS}}{N_h}\left(m\beta_{bloc}\rho_{in} + (1-m)\rho_{out}\right) \\ 0 \\ \frac{\tau_v I_h S_{SR}}{N_h}\left(m\beta_{bloc}\rho_{in} + (1-m)\rho_{out}\right) \\ 0 \\ \frac{\tau_v I_h S_{RR}}{N_h}\left(m\beta_{bloc}\rho_{in} + (1-m)\rho_{out}\right) \\ 0 \end{bmatrix}, \mathcal{V} = \begin{bmatrix} \varphi I_h + (\mu_h + \gamma)I_h \\ \theta E_{SS} + (m\sigma_s + \mu_{vs})E_{SS} \\ (m\sigma_s + \mu_{vr})I_{SS} - \theta E_{SS} \\ \theta E_{SR} + (m\sigma_r + \mu_{vr})E_{SR} \\ (m\sigma_r + \mu_{vr})I_{SR} - \theta E_{SR} \\ \theta E_{RR} + (m\sigma_r + \mu_{vr})E_{RR} \\ (m\sigma_r + \mu_{vr})I_{RR} - \theta E_{RR} \end{bmatrix} \tag{1}$$

Thus, the Jacobian matrices F and V is evaluated at MFE, where $I_h^* = 0$, $R_h^* = 0$, $E_{SS}^* = 0$, $I_{SS}^* = 0$, $E_{SR}^* = 0$, $I_{SR}^* = 0$, $E_{RR}^* = 0$ and $I_{RR}^* = 0$ to obtain;

$$F = \begin{bmatrix} 0 & 0 & \frac{\tau_h S_h^*\omega_{net}}{N_h^*} & 0 & \frac{\tau_h S_h^*\omega_{net}}{N_h^*} & 0 & \frac{\tau_h S_h^*\omega_{net}}{N_h} \\ \frac{\tau_v S_{SS}^*\omega_{net}}{N_h^*} & 0 & 0 & 0 & 0 & 0 & 0 \\ 0 & 0 & 0 & 0 & 0 & 0 & 0 \\ \frac{\tau_v S_{SR}^*\omega_{net}}{N_h^*} & 0 & 0 & 0 & 0 & 0 & 0 \\ 0 & 0 & 0 & 0 & 0 & 0 & 0 \\ \frac{\tau_v S_{RR}^*\omega_{net}}{N_h^*} & 0 & 0 & 0 & 0 & 0 & 0 \\ 0 & 0 & 0 & 0 & 0 & 0 & 0 \end{bmatrix}, V = \begin{bmatrix} \varphi + \mu_h + \gamma & 0 & 0 & 0 & 0 & 0 & 0 \\ 0 & \theta + m\sigma_s + \mu_{vs} & 0 & 0 & 0 & 0 & 0 \\ 0 & -\theta & m\sigma_s + \mu_{vs} & 0 & 0 & 0 & 0 \\ 0 & 0 & 0 & \theta + m\sigma_r + \mu_{vr} & 0 & 0 & 0 \\ 0 & 0 & 0 & -\theta & m\sigma_r + \mu_{vr} & 0 & 0 \\ 0 & 0 & 0 & 0 & 0 & \theta + m\sigma_r + \mu_v & 0 \\ 0 & 0 & 0 & 0 & 0 & -\theta & m\sigma_r + \mu_v \end{bmatrix}$$

where $\omega_{net} = m\beta_{bloc}\rho_{in} + (1-m)\rho_{out}$. The basic reproduction number $\mathcal{R}_0$ is the spectral radius of the next generation matrix FV^{-1} given by;

$$\mathcal{R}_0^L = \sqrt{\frac{\theta\tau_v p^2\omega_{net}N_v^*(K - N_v^*)}{K(m\sigma_s + \mu_{vs})^2(\theta + m\sigma_s + \mu_{vs})} + \frac{q(2p+q)\tau_v\Lambda_v\omega_{net}N_v^*(K - N_v^*)}{K(m\sigma_r + \mu_{vr})^2(\theta + m\sigma_r + \mu_{vr})}} \times \sqrt{\frac{\tau_h\Lambda_h\omega_{net}}{\mu_h(\varphi + \gamma_h + \mu_h)}},$$

and N_v^* is the total mosquito population at MFE.
