# Supplementary material for: Modelling the impact of mosquito bed net utilization on malaria transmission and evolution of pyrethroid resistance
Source: PLoS One. 2026 Jul 15;21(7):e0353301. doi: 10.1371/journal.pone.0353301 (PMC13372178; doi:10.1371/journal.pone.0353301)

PLOS ONE

Modelling the Impact of Mosquito Bed Net Utilization on Malaria Dynamics and Evolution of Pyrethroid Resistance

--Manuscript Draft--

**Manuscript Number:**	PONE-D-25-51344
**Article Type:**	Research Article
**Full Title:**	Modelling the Impact of Mosquito Bed Net Utilization on Malaria Dynamics and Evolution of Pyrethroid Resistance
**Short Title:**	Modelling the Impact of Mosquito Bed Net Utilization on Malaria Dynamics and Evolution of Resistance
**Corresponding Author:**	Sseguya Ivan, Msc.<br>Makerere University<br>Kampala, UGANDA
**Keywords:**	Modelling; Impact of Mosquito Bed Net Utilization; Malaria Dynamics; Evolution of Pyrethroid Resistance
**Abstract:**	Long-lasting Insecticide Nets (LLINs) are central in malaria prevention efforts, although their effectiveness is threatened by the increasing pyrethroid resistance. Despite their wide distribution, many malaria-endemic regions have low LLIN utilization levels. This study formulates a genotype-specific compartmental model accounting for malaria transmission between the human and vector populations. It incorporates the blocking and insecticidal killing effects of LLINs and pyrethroid-induced fitness costs, to evaluate the impact of LLIN utilization on malaria and resistance dynamics. Reproductive numbers, with bed net usage ${\cal{R}}_{0}^{L}$ and without bed net usage ${\cal{R}}_{0}$, were calculated and sensitivity analysis conducted. Results showed that LLIN coverage and effectiveness against pyrethroid-resistant vectors have the highest effect in reducing malaria transmission. Despite an initial LLIN coverage of 81%, without consistent utilization, protection against malaria infection from pyrethroid-only and pyrethroid piperonyl butoxide (PBO) nets, was not sustained over time. PBO nets averted more infections and suppressed malaria vectors by 72% under standard utilization, although this effectiveness declined with shorter retention periods. Also, the study showed that resistance evolves faster with repeated pyrethroid-only campaigns, but PBO nets negate this trend, especially when such resistance carries a higher fitness cost. While shorter LLIN retention slows the evolution of pyrethroid resistance, it is not a viable option for malaria control.
**Order of Authors:**	Sseguya Ivan<br>Joseph Y.T Mugisha<br>Juliet N. Nakakawa
**Additional Information:**	
**Question**	**Response**
**Financial Disclosure**<br><br>Enter a financial disclosure statement that describes the sources of funding for the work included in this submission. Review the submission guidelines for detailed requirements. View published research articles from *PLOS ONE* for specific examples.<br><br>This statement is required for submission	The author(s) received no specific funding for this work.

Powered by Editorial Manager® and ProduXion Manager® from Aries Systems Corporation

and **will appear in the published article** if the submission is accepted. Please make sure it is accurate.<br><br>**Funded studies**<br>Enter a statement with the following details:<br>• Initials of the authors who received each award<br>• Grant numbers awarded to each author<br>• The full name of each funder<br>• URL of each funder website<br>• Did the sponsors or funders play any role in the study design, data collection and analysis, decision to publish, or preparation of the manuscript?<br><br>Did you receive funding for this work?	
**Competing Interests**<br><br>Use the instructions below to enter a competing interest statement for this submission. On behalf of all authors, disclose any competing interests that could be perceived to bias this work—acknowledging all financial support and any other relevant financial or non-financial competing interests.<br><br>This statement is required for submission and **will appear in the published article** if the submission is accepted. Please make sure it is accurate and that any funding sources listed in your Funding Information later in the submission form are also declared in your Financial Disclosure statement.<br><br>View published research articles from *PLOS ONE* for specific examples.	The authors have declared that no competing interests exist.

**NO authors have competing interests**<br><br>Enter: *The authors have declared that no competing interests exist.*<br><br>**Authors with competing interests**<br><br>Enter competing interest details beginning with this statement:<br><br>*I have read the journal's policy and the authors of this manuscript have the following competing interests: [insert competing interests here]*<br><br>* typeset	
**Ethics Statement**<br><br>Enter an ethics statement for this submission. This statement is required if the study involved:<br><br>• Human participants<br>• Human specimens or tissue<br>• Vertebrate animals or cephalopods<br>• Vertebrate embryos or tissues<br>• Field research<br><br>Write "N/A" if the submission does not require an ethics statement.<br><br>General guidance is provided below. Consult the submission guidelines for detailed instructions. **Make sure that all information entered here is included in the Methods section of the manuscript.**	N/A

**Format for specific study types**<br><br>**Human Subject Research (involving human participants and/or tissue)**<br>• Give the name of the institutional review board or ethics committee that approved the study<br>• Include the approval number and/or a statement indicating approval of this research<br>• Indicate the form of consent obtained (written/oral) or the reason that consent was not obtained (e.g. the data were analyzed anonymously)<br><br>**Animal Research (involving vertebrate animals, embryos or tissues)**<br>• Provide the name of the Institutional Animal Care and Use Committee (IACUC) or other relevant ethics board that reviewed the study protocol, and indicate whether they approved this research or granted a formal waiver of ethical approval<br>• Include an approval number if one was obtained<br>• If the study involved *non-human primates*, add *additional details* about animal welfare and steps taken to ameliorate suffering<br>• If anesthesia, euthanasia, or any kind of animal sacrifice is part of the study, include briefly which substances and/or methods were applied<br><br>**Field Research**<br><br>Include the following details if this study involves the collection of plant, animal, or other materials from a natural setting:<br>• Field permit number<br>• Name of the institution or relevant body that granted permission	
**Data Availability**<br><br>Authors are required to make all data underlying the findings described fully available, without restriction, and from the time of publication. PLOS allows rare exceptions to address legal and ethical concerns. See the PLOS Data Policy and FAQ for detailed information.	Yes - all data are fully available without restriction

A Data Availability Statement describing where the data can be found is required at submission. Your answers to this question constitute the Data Availability Statement and **will be published in the article**, if accepted.<br><br>**Important:** Stating 'data available on request from the author' is not sufficient. If your data are only available upon request, select 'No' for the first question and explain your exceptional situation in the text box.<br><br>Do the authors confirm that all data underlying the findings described in their manuscript are fully available without restriction?	
**Describe where the data may be found in full sentences. If you are copying our sample text, replace any instances of XXX with the appropriate details.**<br><br>• If the data are **held or will be held in a public repository**, include URLs, accession numbers or DOIs. If this information will only be available after acceptance, indicate this by ticking the box below. For example: *All XXX files are available from the XXX database (accession number(s) XXX, XXX.).*<br>• If the data are all contained **within the manuscript and/or Supporting Information files**, enter the following: *All relevant data are within the manuscript and its Supporting Information files.*<br>• If neither of these applies but you are able to provide **details of access elsewhere**, with or without limitations, please do so. For example:<br><br>*Data cannot be shared publicly because of [XXX]. Data are available from the XXX Institutional Data Access / Ethics Committee (contact via XXX) for researchers who meet the criteria for access to confidential data.*<br><br>*The data underlying the results presented in the study are available from (include the name of the third party*	All relevant data are within the manuscript and its Supporting Information files.

and contact information or URL).<br>• This text is appropriate if the data are owned by a third party and authors do not have permission to share the data.<br><br>* typeset	
Additional data availability information:	

Powered by Editorial Manager® and ProduXion Manager® from Aries Systems Corporation

Manuscript
Click here to access/download;Manuscript;Sseguya_ivan_manuscript.pdf

Modelling the Impact of Mosquito Bed Net Utilization on Malaria Dynamics and Evolution of Pyrethroid Resistance

Ivan Sseguya[a,*], Joseph Y.T Mugisha[a], Juliet N. Nakakawa[a], Prashanth Selvaraj[b], Jonathan Kayondo[c]

[a]*Department of Mathematics, Makerere University, Kampala, Uganda*
[b]*Institute for Disease Modeling, Bellevue, Washington, United States of America*
[c]*Department of Entomology, Uganda Virus Research Institute, Entebbe, Uganda*

Abstract

Long Lasting Insecticide Nets (LLINs) are central in malaria prevention efforts, although their effectiveness is threatened by the increasing pyrethroid resistance. Despite their wide distribution, many malaria-endemic regions have low LLIN utilization levels. This study formulates a genotype-specific compartmental model accounting for malaria transmission between the human and vector populations. It incorporates the blocking and insecticidal killing effects of LLINs, and pyrethroid-induced fitness costs, to evaluate the impact of LLIN utilization on malaria and resistance dynamics. Reproductive numbers, with bed net usage $\mathcal{R}_0^L$ and without bed net usage $\mathcal{R}_0$, were calculated and sensitivity analysis conducted. Results showed that LLIN coverage and effectiveness against pyrethroid-resistant vectors have the highest effect in reducing malaria transmission. Despite of an initial LLIN coverage of 81%, without consistent utilization, protection against malaria infection from pyrethroid-only and pyrethroid piperonyl butoxide (PBO) nets, was not sustained over time. PBO nets averted more infections and suppressed malaria vectors by 72% under standard utilization, although this effectiveness declined with shorter retention periods. Also, the study showed that resistance evolves faster with repeated pyrethroid-only campaigns, but PBO nets negate this trend especially when such resistance carries a higher fitness cost. While shorter LLIN retention slows the evolution of pyrethroid resistance, it is not a viable option for malaria

*Corresponding author
Email addresses: isseguya@gmail.com (Ivan Sseguya), joseph.mugisha@mak.ac.ug (Joseph Y.T Mugisha), (Juliet N. Nakakawa), (Prashanth Selvaraj), (Jonathan Kayondo)

control.

Keywords: Pyrethroid resistance, Long Lasting Insecticide Nets (LLINs), Bed net Utilization, Malaria Prevalence, Piperonyl Butoxide (PBO)

1. Introduction

Malaria is a major global threat, accounting for an estimated 597 000 deaths in 2023 with a mortality rate of 13.7 per 1000 people, despite the substantial gains in the past two decades [1]. The World Health Organization (WHO) African region persistently bears the greatest burden, representing about 94% of the global malaria cases and 95% of malaria-related deaths worldwide by 2023 [1]. Uganda, one of the top five highly burdened countries, ranks third with 5% of the world's malaria cases and 2793 deaths in 2023 [2]. The disease is endemic in 95% of the country, contributing between 30% to 50% of outpatient visits, 15% to 20% of all hospital admissions, and up to 20% of all hospital deaths [3].

Vector control is fundamental in malaria prevention efforts, especially in sub-Saharan Africa [4], aiming to eliminate malaria-transmitting vectors or inhibit their ability to blood feed [5]. Primary vector control tools mainly include Long-Lasting Insecticidal Nets (LLINs) and Indoor Residual Spraying (IRS) [3], but other tools like larviciding, larval source management, and improved housing are used in public health malaria campaigns [6, 7, 8]. LLINs and or IRS are the most effective vector control tools used in malaria public health campaigns. For instance, a systematic review found that IRS deployment in malaria control programs, particularly with at least 80% coverage, is highly effective against malaria infections [9], and the use of LLINs across sub-Saharan Africa between 2000 and 2015 averted approximately 68% of 663 million malaria cases registered globally [10]. In Uganda, IRS usage was associated with a reduction in malaria morbidity post its application, although its effectiveness was short-lived, lasting about six months under field conditions [11].

Despite ~~of~~ the LLIN effectiveness, their continued usage or wide coverage exert high levels of selection pressure on malaria vectors, causing them to evolve resistance towards insecticides like pyrethroids, commonly used in malaria public health interventions [12]. This

has compromised the effectiveness of LLIN campaigns over years [13], with such resistance reported in a many countries including Uganda [14], Ghana [15], Malawi [16], Kenya [17] among others. In an effort to address this challenge, next generational nets like Interceptor® G2 which combines pyrethroids with Chlorfenapyr [18], PermaNet® 3.0 and Olyset® Plus which combine pyrethroids with piperonyl butoxide (PBO), Royal Guard® Net which combines pyrethroids and pyriproxyfen, and DawaPlus® 3.0 which combines pyrethroids with PBO, have been developed to improve LLIN effectiveness in high insecticide-resistance settings [19]. The success of these campaigns depends on bed net coverage, consistent bed net utilization, vector behaviour patterns, timely replacement, and chemical and physical durability of the bed nets [20, 21]. LLIN utilisation remains low, especially within African countries [22, 23], despite the substantial impact of LLINs on malaria transmission over the years. A modelling analysis found that 35 out of 40 African countries have a median bed net retention time of 1.64 years, with Uganda having a less than 2-year average bed net retention time [24].

Malaria modelling, models have been used to assess the impact of LLIN interventions on malaria dynamics [25, 26, 27] and to study the development and spread of insecticide-resistance within the mosquito population [28, 29]. While there are several studies that have addressed related questions on LLIN utilization [30, 31, 32], limited work has focused on the impact of bed net utilization patterns on malaria transmission dynamics and evolution of pyrethroid resistance. This study investigates how temporal decay in bed net utilization impacts malaria burden and the evolution of pyrethroid resistance within the mosquito population.

2. Model Formulation

In this study, we modify an existing deterministic model [33] by consolidating the human population and emphasizing mosquito populations in malaria dynamics. This model categorizes the human population into susceptible humans (S_h), infectious humans I_h and those recovered from malaria infection upon treatment or natural recovery R_h. Therefore, the total human population N_h is given by; $N_h = S_h + I_h + R_h$.The susceptible population S_h grows

at a constant natality rate of Λ_h and rate ϑ as recovered individuals lose their temporary immunity. However, this reduces due to new malaria infections obtained through indoor or outdoor biting at rates ρ_{in} and ρ_{out} respectively, and natural mortality at the rate μ_h.

An allele is a variant form of the same gene that occurs at a specific position on a chromosome, while a genotype is a genetic constitution of an organism, particularly a combination of alleles at one or more loci [34, 35]. Alleles come from mutations, and they contribute to genetic diversity, influencing traits like insecticide resistance. Therefore, the mosquito population consists of two alleles in its genetic pool: pyrethroid sensitive allele S and pyrethroid resistant allele R, with corresponding allele frequencies $p(t)$ and $q(t)$. The vector population, in model system (2.2), is categorized according to the stage of infection (i.e susceptible, latently infected, and infectious mosquitoes), and genotype (i.e, homozygous sensitive SS, heterozygous SR, and homozygous resistant RR). The total vector population is classified into nine epidemiological compartments which include: homozygous sensitive susceptible S_{SS}, latently infected E_{SS}, and infectious I_{SS} mosquitoes; heterozygous susceptible S_{SR}, latently infected E_{SR}, and infectious I_{SR} mosquitoes; and homozygous resistant susceptible S_{RR}, latently infected E_{RR}, and infectious I_{RR} for pyrethroid resistant vectors. With this grouping, It follows that; $N_v = N_{SS} + N_{SR} + N_{RR}$, where $N_{SS} = S_{SS} + E_{SS} + I_{SS}$, $N_{SR} = S_{SR} + E_{SR} + I_{SR}$ and $N_{RR} = S_{RR} + E_{RR} + I_{RR}$. Recruitment of new mosquitoes is modelled using a logistic function of the form $\Lambda_v N_v \left(1 - \frac{N_v}{K}\right)$, where Λ_v denotes the intrinsic mosquito recruitment rate, and K is the environmental carrying capacity for the mosquito population. At recruitment, the proportion of new homozygous sensitive, heterozygous and homozygous resistant genotypes at any time t, is given by $p^2(t)$, $2p(t)q(t)$ and $q^2(t)$ respectively, where allele frequencies $p(t)$ and $q(t)$ [36] are given by:

$$p(t) = \frac{N_{SS}(t) + 0.5N_{SR}(t)}{N_v(t)}, \text{and } q(t) = \frac{N_{RR}(t) + 0.5N_{SR}(t)}{N_v(t)}, \tag{2.1}$$

with $N_v(t)$, N_{SS}, N_{SR} and N_{RR} maintaining their original meaning.

The deployment of bed nets interrupt malaria transmission through blocking mosquitoes from blood feeding at a rate LLIN_{bloc}, and killing malaria vectors upon making physical

contact with bed nets at rates LLIN_{eff} and LLIN_{effr} for sensitive and resistant mosquitoes respectively. This formulation is supported by the assumptions: humans have the same risk of exposure to malaria infection regardless of age, humans acquire no adaptive immunity over years, female vectors have enough males to fertilize them [37], mosquitoes are uniformly distributed with equal access to human hosts, and there is no change in mosquito behaviour as a response to LLIN campaigns. The human and mosquito interaction and malaria dynamics are represented in the compartmental diagram in Figure (1), where the logistic term

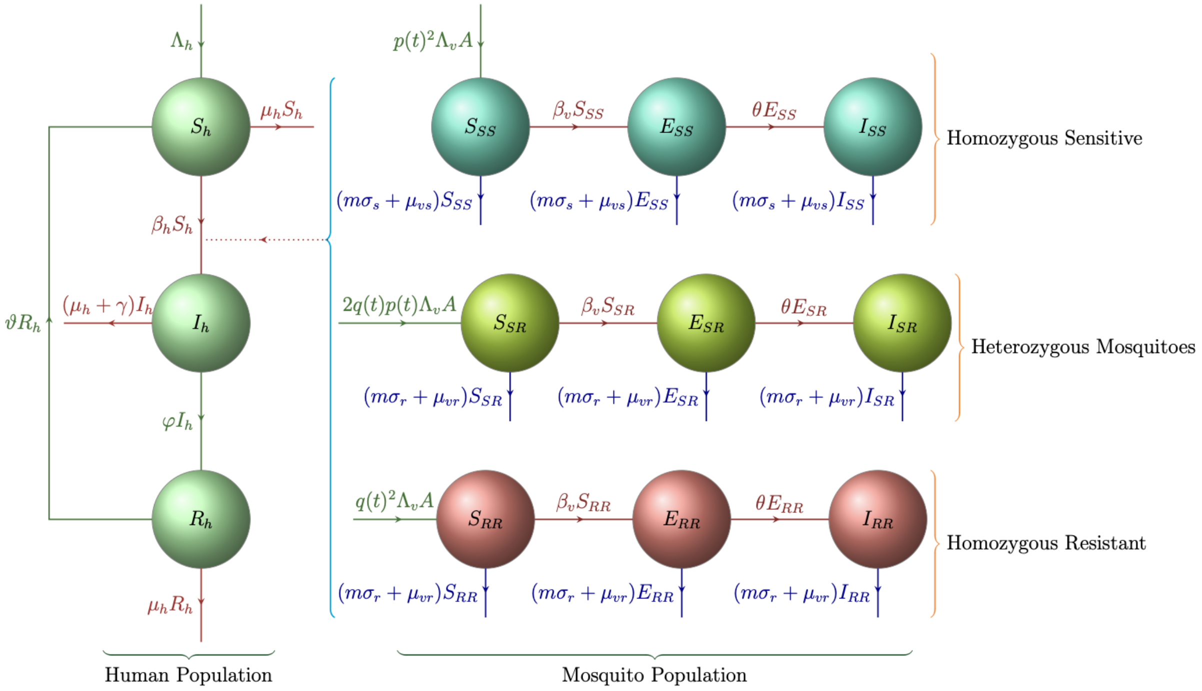

Figure 1: Compartmental diagram for the Mosquito Genetic Classification and Malaria dynamics between Human and Vector populations

$A = N_v\left(1-\dfrac{N_v}{K}\right)$, human force of infection $\beta_h = \dfrac{\tau_h(I_{SS}+I_{SR}+I_{RR})}{N_h}\big(m\beta_{bloc}\rho_{in}+(1-m)\rho_{out}\big)$ and mosquito force of infection $\beta_v = \dfrac{\tau_v I_h}{N_h}\big(m\beta_{bloc}\rho_{in}+(1-m)\rho_{out}\big)$ respectively. This com-

partmental diagram in Figure (1) leads to the system of model equations:

$$
\begin{aligned}
\frac{dS_h}{dt} =& \Lambda_h + \vartheta R_h - \frac{\tau_h(I_{SS}+I_{SR}+I_{RR})S_h}{N_h}\big(m\beta_{bloc}\rho_{in} + (1-m)\rho_{out}\big) - \mu_h S_h,\\
\frac{dI_h}{dt} =& \frac{\tau_h(I_{SS}+I_{SR}+I_{RR})S_h}{N_h}\big(m\beta_{bloc}\rho_{in} + (1-m)\rho_{out}\big) - \varphi I_h - (\mu_h+\gamma)I_h,\\
\frac{dR_h}{dt} =& \varphi I_h - \vartheta R_h - \mu_h R_h,\\
\frac{dS_{SS}}{dt} =& p^2\Lambda_v N_v\left(1-\frac{N_v}{K}\right) - \frac{\tau_v I_h S_{SS}}{N_h}\big(m\beta_{bloc}\rho_{in} + (1-m)\rho_{out}\big) - (m\sigma_s+\mu_{vs})S_{SS},\\
\frac{dE_{SS}}{dt} =& \frac{\tau_v I_h S_{SS}}{N_h}\big(m\beta_{bloc}\rho_{in} + (1-m)\rho_{out}\big) - \theta E_{SS} - (m\sigma_s+\mu_{vs})E_{SS},\\
\frac{dI_{SS}}{dt} =& \theta E_{SS} - (m\sigma_s+\mu_{vs})I_{SS},\\
\frac{dS_{SR}}{dt} =& 2pq\Lambda_v N_v\left(1-\frac{N_v}{K}\right) - \frac{\tau_v I_h S_{SR}}{N_h}\big(m\beta_{bloc}\rho_{in} + (1-m)\rho_{out}\big) - (m\sigma_r+\mu_{vr})S_{SR},\\
\frac{dE_{SR}}{dt} =& \frac{\tau_v I_h S_{SR}}{N_h}\big(m\beta_{bloc}\rho_{in} + (1-m)\rho_{out}\big) - \theta E_{SR} - (m\sigma_r+\mu_{vr})E_{SR},\\
\frac{dI_{SR}}{dt} =& \theta E_{SR} - (m\sigma_r+\mu_{vr})I_{SR}.\\
\frac{dS_{RR}}{dt} =& q^2\Lambda_v N_v\left(1-\frac{N_v}{K}\right) - \frac{\tau_v I_h S_{RR}}{N_h}\big(m\beta_{bloc}\rho_{in} + (1-m)\rho_{out}\big) - (m\sigma_r+\mu_{vr})S_{RR}.\\
\frac{dE_{RR}}{dt} =& \frac{\tau_v I_h S_{RR}}{N_h}\big(m\beta_{bloc}\rho_{in} + (1-m)\rho_{out}\big) - \theta E_{RR} - (m\sigma_r+\mu_{vr})E_{RR},\\
\frac{dI_{RR}}{dt} =& \theta E_{RR} - (m\sigma_r+\mu_{vr})I_{RR},
\end{aligned}
\tag{2.2}
$$

where $\sigma_s = \text{LLIN}_{cov} \times f \times \text{LLIN}_{eff}$, $\sigma_r = \text{LLIN}_{cov} \times f \times \text{LLIN}_{effr}$, $\beta_{bloc} = 1 - (\text{LLIN}_{cov} \times f \times \text{LLIN}_{bloc})$, LLIN_{cov} is the LLIN coverage at population level, LLIN_{eff} is the pyrethroid killing effectiveness, LLIN_{bloc} is the LLIN blocking effect, m is the proportion of mosquito vectors that bite indoors and f is the proportion of indoor biting mosquitoes that make contact with bed nets.

3. Basic Properties of the Model

All model parameters to model system (2.2) - including the LLIN blocking effectiveness β_{bloc}, and pyrethroid induced death rates in sensitive and resistant mosquitoes σ_s and σ_r respectively are constant for all time. The solutions to this system remain positive and bounded within the epidemiological feasible region ξ for all $t \geq 0$, which ensures that the

system is mathematically well-posed. The Mosquito population is regulated by the carrying capacity K while the human population dynamics are determined by $\Lambda_h - \mu_h$ (see subsection 6.0.1 in Appendix section). Therefore, these properties guarantee that long-term malaria predictions remain within biologically realistic limits.

3.1. Equilibrium points and Malaria Transmission Reproductive Number

This section presents the malaria free equilibrium point (MFE), existence of endemic steady states, and discusses the malaria transmission reproduction number with and without the use of LLIN bed net intervention. When no bed nets are deployed, the total mosquito biting rate $\omega = m\rho_{in} + (1-m)\rho_{out}$, and mosquito mortality rate $\mu_v = \mu_{vr} = \mu_{vs}$ (assuming no fitness costs are incurred by malaria vectors). Therefore, for $\Lambda_v > \mu_v$, the malaria free equilibrium point is given by; $E_0 = (S_h^*, I_h^*, R_h^*, S_{SS}^*, E_{SS}^*, I_{SS}^*, S_{SR}^*, E_{SR}^*, I_{SR}^*, S_{RR}^*, E_{RR}^*, I_{RR}^*)$, such that;

$$E_0 = \left(\frac{\Lambda_h}{\mu_h}, 0, 0, \frac{p^2K(\Lambda_v - \mu_v)}{\mu_v\Lambda_v}, 0, 0, \frac{2pqK(\Lambda_v - \mu_v)}{\mu_v\Lambda_v}, 0, 0, \frac{q^2K(\Lambda_v - \mu_v)}{\mu_v\Lambda_v}, 0, 0\right).$$

The Malaria Transmission reproduction number $\mathcal{R}_0$ is obtained using the next generation matrix method [38], and given by:

$$\mathcal{R}_0 = \sqrt{\frac{\omega\tau_h\Lambda_h}{\mu_h(\varphi + \gamma + \mu_h)}} \times \sqrt{\frac{\omega\tau_v\theta K(\Lambda_v - \mu_v)}{\mu_v\Lambda_v(\theta + \mu_v)}}. \tag{3.1}$$

The threshold $\mathcal{R}_0$ in equation 3.1, represents the expected number of secondary malaria infections produced by a single infectious individual over the duration of infection provided that everyone else in the population is susceptible, in absence of bed net interventions. The square root on $\mathcal{R}_0$ represents a two-phase infection process that occurs in malaria transmission across the human and vector population. This is because it requires two generations of infections, human to mosquito and mosquito to human, to obtain a secondary human infection from an infectious human [39]. The first square root term (infectious mosquitoes to new human infections) accounts for the expected number of infected humans an infectious mosquito will produce during its lifetime, depending on demographic factors like Λ_h and Λ_v. On the other hand, the second square root term (infectious humans to new mosquito infections) accounts for the expected number of infectious mosquitoes arising from one in-

fectious human during his infectious period, scaled by the chance that a latently infected vector survives the exposure period and mosquito demographic parameters.

When a bed net campaign is deployed, the effective mosquito biting rate $\omega_{net} = m\beta_{bloc}\rho_{in} + (1-m)\rho_{out}$, $\mu_{vr} \neq \mu_{vs}$ and the new malaria transmission reproduction number $\mathcal{R}_0^L$ is given by:

$$\begin{aligned}
\mathcal{R}_0^L &= \mathcal{R}_0^h \times \mathcal{R}_0^v, \quad \text{where,}\\
R_0^h &= \sqrt{\frac{\tau_h \Lambda_h \omega_{net}}{\mu_h(\varphi + \gamma_h + \mu_h)}},\\
\mathcal{R}_0^v &= \sqrt{\frac{\theta \tau_v p^2 \omega_{net} N_v^*(K - N_v^*)}{K(m\sigma_s + \mu_{vs})^2(\theta + m\sigma_s + \mu_{vs})} + \frac{q(2p+q)\tau_v \Lambda_v \omega_{net} N_v^*(K - N_v^*)}{K(m\sigma_r + \mu_{vr})^2(\theta + m\sigma_r + \mu_{vr})}}
\end{aligned}$$

and N_v^* is the total mosquito population at MFE. Therefore, R_0^L represents the expected number of secondary malaria infections produced by a single infectious individual over the duration of infection, provided that everyone else in the population is susceptible, when a bed net campaign is implemented. This depends on, but not limited to, impact of LLINs in blocking mosquitoes from blood feeding, mosquito biting rate, the human recovery and death rate etc. $\mathcal{R}_0^v$ is modified with terms $\dfrac{\theta \tau_v p^2 \omega_{net} N_v^*(K - N_v^*)}{K(m\sigma_s + \mu_{vs})^2(\theta + m\sigma_s + \mu_{vs})}$ and $\dfrac{q(2p+q)\tau_v \Lambda_v \omega_{net} N_v^*(K - N_v^*)}{K(m\sigma_r + \mu_{vr})^2(\theta + m\sigma_r + \mu_{vr})}$ representing the expected number of infectious pyrethroid sensitive and resistant vectors respectively, arising from an infectious human during his infectious period, adjusted by the rate of LLIN killing, progression into infectious state and natural mosquito mortality. Since the system (2.2) satisfies axioms (A1) to (A5) in [38] and $\mathcal{R}_0^L$ is biologically meaningful, using Theorem 2 of [38] the result below holds;

Result 1. *The MFE state for system* (2.2) *is locally asymptotically stable if* $\mathcal{R}_0 < 1$ *and unstable if* $\mathcal{R}_0 > 1$.

Result (1) means that malaria infection can be eliminated from human and mosquito population when $\mathcal{R}_0 < 1$ if the initial sizes of the sub-populations of system (2.2) are in the basin of attraction of the malaria - free steady state.

Generally, the equilibrium points of the model system 2.2 are given by:

$$
\begin{aligned}
S_h^* =& \frac{\Lambda_h(\vartheta+\mu_h)(\vartheta+\gamma+\mu_h)}{(\vartheta+\mu_h)(\mu_h+\gamma)(\mu_h+\mathcal{F}_{hv}^*)+\mu_h\varphi(\mathcal{F}_{hv}^*+\vartheta+\mu_h)}, \quad S_{SS}^* = \frac{p^2K(\Lambda_v-\mu_v)}{\Lambda_v(\mathcal{F}_{vh}^*+m\sigma_s+\mu_v)},\\
I_h^* =& \frac{\mathcal{F}_{hv}^*\mathcal{F}_{hv}^*(\vartheta+\mu_h)}{\mu_h\varphi\mathcal{F}_{hv}^*+(\vartheta+\mu_h)[(\mu_h+\gamma)\mathcal{F}_{hv}^*+(\vartheta+\gamma+\mu_h)\mu_h]}, \quad S_{RR}^* = \frac{q^2K(\Lambda_v-\mu_v)}{\Lambda_v(\mathcal{F}_{vh}^*+m\sigma_r+\mu_v)},\\
R_h^* =& \frac{\varphi\Lambda\mathcal{F}_{hv}^*}{\mu_h\varphi\mathcal{F}_{hv}^*+(\vartheta+\mu_h)[(\mu_h+\gamma)\mathcal{F}_{hv}^*+(\vartheta+\gamma+\mu_h)\mu_h]}, \quad S_{SR}^* = \frac{2pqK(\Lambda_v-\mu_v)}{\Lambda_v(\mathcal{F}_{vh}^*+m\sigma_r+\mu_v)},\\
E_{SS} =& \frac{p^2K(\Lambda_v-\mu_v)\mathcal{F}_{vh}^*}{\Lambda_v(\theta+m\sigma_s+\mu_v)(\mathcal{F}_{vh}^*+m\sigma_s+\mu_v)}, \quad E_{SR}^* = \frac{2pqK(\Lambda_v-\mu_v)\mathcal{F}_{vh}^*}{\Lambda_v(\theta+m\sigma_r+\mu_v)(\mathcal{F}_{vh}+m\sigma_r+\mu_v)},\\
E_{RR}^* =& \frac{q^2K(\Lambda_v-\mu_v)\mathcal{F}_{vh}^*}{\Lambda_v(\theta+m\sigma_r+\mu_v)(\mathcal{F}_{vh}^*n+m\sigma_r+\mu_v)}, \quad I_{SS}^* = \frac{p^2\theta K(\Lambda_v-\mu_v)\mathcal{F}_{vh}^*}{\Lambda_v A_s},\\
I_{SR}^* =& \frac{2pq\theta K(\Lambda_v-\mu_v)\mathcal{F}_{vh}^*}{\Lambda_v A_r}, \quad I_{RR}^* = \frac{q^2\theta K(\Lambda_v-\mu_v)\mathcal{F}_{vh}^*}{\Lambda_v A_r},
\end{aligned} \tag{3.2}
$$

where $\mu_v = \mu_{vs} = \mu_{vr}$, $A_s = (m\sigma_s+\mu_v)(\theta+m\sigma_s+\mu_v)(\mathcal{F}_{vh}^*+m\sigma_s+\mu_v)$, $A_r = (m\sigma_r+\mu_v)(\theta+m\sigma_r+\mu_v)(\mathcal{F}_{vh}^*+m\sigma_r+\mu_v)$, and:

$$
\mathcal{F}^*{}_{hv} = \frac{\theta\tau_h K(\Lambda_v-\mu_v)\mathcal{F}^*{}_{vh}}{\Lambda_v}\left[\frac{p^2}{A_s}+\frac{q(2p+q)}{A_r}\right](m\beta_{bloc}\rho_{in}+(1-m)\rho_{out}), \tag{3.3}
$$

$$
\mathcal{F}_{vh}^* = \frac{\tau_v\Lambda_h\mathcal{F}_{hv}^*(\vartheta+\mu_h)(m\beta_{bloc}\rho_{in}+(1-m)\rho_{out})}{\mu_h\varphi\mathcal{F}_{hv}^*+(\vartheta+\mu_h)[(\mu_h+\gamma)\mathcal{F}_{hv}^*+(\vartheta+\gamma+\mu_h)\mu_h]}. \tag{3.4}
$$

Substituting equation 3.4 into the human force of infection equation 3.3, yields the following:

$$
\mathcal{F}^*{}_{hv}\left[a\mathcal{H}_s\mathcal{H}_r\mathcal{F}^{*2}_{hv}+\mathcal{D}_1\mathcal{F}^*{}_{hv}+\mathcal{D}_2\right]=0, \tag{3.5}
$$

where $\mathcal{D}_1 = aB[f_r\mathcal{H}_s+f_s\mathcal{H}_r]-[C_sB\mathcal{H}_r+C_r\mathcal{H}_s]$, $\mathcal{D}_2 = Bf_sf_r\left(aB-\frac{C_s}{f_s}-\frac{C_r}{f_r}\right)$, $a = \dfrac{\Lambda_v}{\theta\tau_h\tau_v\Lambda_hK(\Lambda_v-\mu_v)(\vartheta+\mu_h)(m\beta_{bloc}\rho_{in}+(1-m)\rho_{out})^2}$, $\mathcal{H}_s = \tau_v\Lambda_h(\vartheta+\mu_h)(m\beta_{bloc}\rho_{in}+(1-m)\rho_{out})+[\varphi\mu_h+(\vartheta+\mu_h)(\gamma+\mu_h)](m\sigma_s+\mu_v)$, $\mathcal{H}_r = \tau_v\Lambda_h(\vartheta+\mu_h)(m\beta_{bloc}\rho_{in}+(1-m)\rho_{out})+[\varphi\mu_h+(\vartheta+\mu_h)(\gamma+\mu_h)](m\sigma_r+\mu_v)$, $C_s = \dfrac{p^2}{(m\sigma_s+\mu_v)(\theta+m\sigma_s+\mu_v)}$, $C_r = \dfrac{q(2p+q)}{(m\sigma_r+\mu_v)(\theta+m\sigma_r+\mu_v)}$ and $B = (\vartheta+\mu_h)(\vartheta+\gamma+\mu_h)\mu_h$. From equation 3.2, if $\mathcal{F}^*{}_{hv}, \mathcal{F}^*{}_{vh}, \sigma_s, \sigma_r, \theta, \vartheta, \varphi = 0$ the equilibrium point obtained represents the MFE steady state. For malaria endemic equilib-

rium state, $\mathcal{F}^*{}_{hv}, \mathcal{F}^*{}_{vh}, \sigma_s, \sigma_r, \theta, \vartheta, \varphi > 0$. Therefore, the endemic equilibrium point exists if;

$$\mathcal{F}^*{}_{hv} = \frac{-\mathcal{D}_1 \pm \sqrt{\mathcal{D}_1^2 - 4a\mathcal{H}_s\mathcal{H}_r\mathcal{D}_2}}{2a\mathcal{H}_s\mathcal{H}_r}, \tag{3.6}$$

for $\mathcal{D}_1^2 \geq 4a\mathcal{H}_s\mathcal{H}_r\mathcal{D}_2$. This implies that $\mathcal{F}^*{}_{hv}$ can take on two values which demonstrates a possibility of existence of two endemic equilibrium points.

4. Results of the Study

This section investigates five LLIN utilization scenarios on malaria and resistance dynamics across three successive bed net campaigns. The baseline scenario involves no bed net usage, while the standard utilization scenario considers effective coverage to gradually decline to 1% over four years. In the three-year, two-year, and one-year utilization scenarios, effective bed net coverage drops to 1% after three, two and one year(s) respectively. All scenarios, save for baseline, deploy three successive bed net campaigns with the first two deployments using pyrethroid-only nets and the third deployment using nets treated with pyrethroid insecticides and a PBO synergist. These scenarios are compared in terms of selecting mosquitoes for resistance, suppressing vector populations and impacting malaria dynamics.

The study considers that 60% of the mosquito population blood feeds indoors and of this, 70% makes actual contact with LLINs. The initial susceptible $S_h(0)$, infectious $I_h(0)$ and recovered $R_h(0)$ human population was set at 1.5 million, 100 and zero people respectively. A carrying capacity of 6 million vectors was set with initial mosquito numbers of $S_{SS}(0) = 7500$, $E_{SS}(0) = 500$, $I_{SS}(0) = 100$, $S_{sr}(0) = 1150$, $E_{sr}(0) = 600$, $I_{sr}(0) = 50$, $S_{rr}(0) = 75$, $E_{rr}(0) = 20$ and $I_{rr}(0) = 5$. Biologically relevant life history parameter values obtained from published scientific articles (see Table 1) and assumed initial variables were used to numerically simulate the model system (2.2) using Python 3.10.11 software package.

For all simulations, model parameters like bed net coverage LLIN_{cov}, blocking effect $\text{LLIN}_{\text{bloc}}$ and pyrethroid killing effect LLIN_{eff}, in both sensitive and resistant mosquitoes were considered to decay exponentially over time at rates δ_{cov}, δ_{bloc} and δ_{eff} respectively. The PBO

synergist with an initial effectiveness of $PBO_{eff} = 0.68$ [40, 41], decayed exponentially at the rate δ_{pbo} such that $\delta_{pbo} = 0.0002324$ and $\delta_{pbo} = 0.001156$ for year one and two of the bed net campaign respectively [42]. Additionally, since the investigations run across multiple LLIN campaigns, the rate of new mosquito recruitment $\Lambda_v(t)$ follows a seasonal pattern such that;

$$\Lambda_v(t) = \Lambda_v(0)\Bigg(1 + A_1 \sin\big(\frac{2\pi t}{T} + \phi_1\big) + A_2 \sin\left(\frac{4\pi t}{T} + \phi_2\right)\Bigg), \tag{4.1}$$

where $\Lambda_v(0)$ is the baseline mosquito recruitment rate, A_1 and A_2 are amplitudes of seasonal cycles [43, 44]. Parameters A_1 and A_2 correspond to the primary annual cycle associated with the first seasonal peak and the secondary semi-annual cycle responsible for the second seasonal peak, respectively. Phase shift parameters ϕ_1 and ϕ_2 control the timing at which rainfall peaks occur within the year, such that $\phi_1 = \dfrac{2 \times 120\pi}{T}$ and $\phi_2 = \dfrac{4 \times 300\pi}{T}$ for $T = 365$ days. Each campaign deployed LLINs 30 days into the year, towards the start of the rainy season for maximum impact [31]. However, the bed net distribution is not an instantaneous activity, often spanning between weeks to months. Therefore, a sigmoid function was used to model the growth of bed net effective coverage from zero to a maximum target of 81%, as follows:

$$f(x) = \frac{L}{1 + e^{-k(x-x_0)}},$$

where L is the target effective coverage ($L = 0.81$), x is time in years, x_0 is the time taken for coverage to reach 50% of the target effective coverage, and k represents the rate of increase in utilization level.

Fitness costs in pyrethroid-resistant malaria vectors impact their survival, gonotrophic cycle duration, fecundity, and their adult lifespan. The effect of pyrethroid resistance on mosquito adult longevity remains inconclusive. While some studies have reported that resistance, particularly in Anopheles gambiae, reduces adult longevity [45, 46, 47], others have demonstrated an increase in adult life span among resistant mosquitoes [48, 49, 50]. However, resistance mechanisms such as overexpression of detoxification enzymes or target site mutation require substantial resources, and these can be diverted from other life-history traits like fecundity and longevity [51].

Table 1: Parameters for the Mosquito Resistance Genetic classification model with malaria dynamics

Parameter	Description	Unit	Value	Reference
τ_h	Probability of an infectious mosquito transmitting malaria to a susceptible human		0.092	[52]
τ_v	Probability of an infectious human transmitting malaria parasites to a susceptible mosquito		0.167	[53, 54]
ϑ	Rate at which individuals that have recovered from malaria infection become susceptible	Per day	1/(0.5x365)	[55]
ρ_{in}	Indoor biting rate	Bites per person per day	17.3	[56]
ρ_{out}	Outdoor biting rate	Bites per person per day	2.3	[56]
Λ_h	Human Population growth rate	per day	0.00009	[57]
Λ_v	Pyrethroid sensitive mosquito recruitment rate	per day	0.25	[58, 59]
Λ_{vr}	Pyrethroid resistant mosquito recruitment rate	per day	$0.25 \times (1 - c)$	[58, 59]
μ_h	Human natural death rate	Per day	1/(68.2 x 365)	[57]
φ	Recovery rate of infectious humans from malaria infection	Per day	1/ 14	[60]
θ	Rate at which exposed mosquito vectors become infectious	Per day	1/10	[61]
γ	Rate at which humans die from malaria	Per day	0.00138	[62]
μ_{vs}	Pyrethroid sensitive Mosquito natural death rate	Per day	1 / 14	[63]
μ_{vr}	Pyrethroid resistant Mosquito natural death rate	Per day	w× 1 / 14	[45, 46]
LLIN_{cov}	LLIN initial bed net coverage		0.81	[42]
LLIN_{eff}	LLIN initial killing effectiveness in sensitive mosquitoes		0.98	[64, 65]
LLIN_{effr}	LLIN initial killing effectiveness in resistant vectors		0.55	[64]
LLIN_{bloc}	LLIN initial blocking effect		0.85	[66, 42]
PBO_{eff}	PBO initial synergistic effect		0.68	[40, 41]
δ_{eff}	Rate of decay of pyrethroid killing effectiveness in sensitive vectors	Per day	0.0031404	Estimated
δ_{effr}	Rate of decay of pyrethroid killing effectiveness in resistant vectors	Per day	0.0027447	Estimated
δ_{bloc}	Rate at which LLIN blocking effect decays	Per day	0.00164	[42]
δ_{cov}	Standard Rate at which LLIN coverage decays	Per day	0.0030099	[24]
δ_{pbo}	Rate at which PBO effect decays	Per day	0.0002324 and 0.001156	[42]
m	Proportion of mosquitoes that feed indoors		0.6	[67]
f	Proportion of indoor bitters that make physical contact with bed nets		0.70	Assumed
c	Modification parameter for fitness cost in mosquito recruitment		0.1	[68]
w	Modification parameter for fitness cost in mosquito adult lifespan		1.15	[68]

4.1. Sensitivity Analysis

To investigate the effect of parameter variations on malaria transmission in the model system (2.2), Sobol and Partial Rank Correlation Coefficient (PRCC) sensitivity analysis on $\mathcal{R}_0^L$ was conducted. A total of 131072 points in the parameter space for Sobol sampling using `SALib.sample.sobol` Python package was used to compute $\mathcal{R}_0^L$ value and generate both Sobol and PRCC sensitivity analyses in Figure 2.

Figure 2 shows that bed net coverage (LLIN_{cov}) and pyrethroid killing effectiveness for resistant mosquitoes (LLIN_{effr}) have the greatest impact on $\mathcal{R}_0^L$, negatively affecting this threshold. Higher bed net coverage and killing effectiveness against resistant vectors leads to a substantial reduction in malaria transmission rate. Therefore, the number of human infections caused by a single infectious individual during an LLIN campaign increases with higher values of model parameters like mosquito recruitment rate (Λ), biting rates (ρ_{in}) and (ρ_{out}), malaria parasite transmission probabilities (τ_h) and (τ_v), and disease progression rate within vectors (θ), although θ has the least effect on $\mathcal{R}_0^L$.

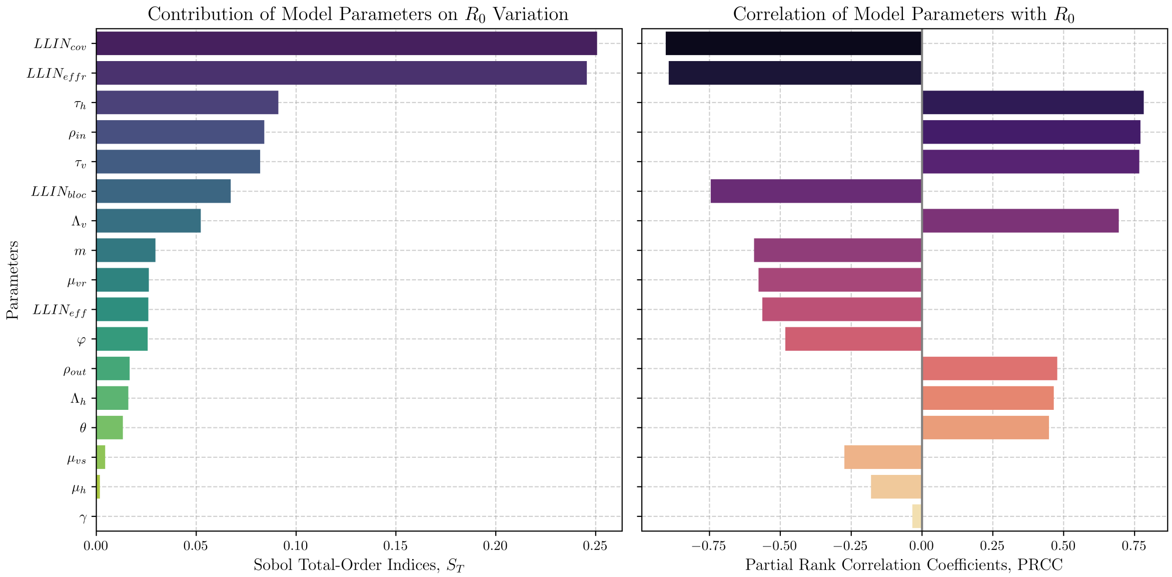

Figure 2: Sensitivity of malaria transmission reproduction number during bed net usage $\mathcal{R}_0^L$ to changes in model parameters

On the other hand, reduction in bed net efficacies (LLIN_{eff} and LLIN_{effr}), percentage of LLIN coverage (LLIN_{cov}), vector natural mortality rates (μ_{vs} and μ_{vr}), human natural and malaria induced death rates (μ_h and γ), and proportion of indoor biting vectors m decrease $\mathcal{R}_0^L$ threshold, with γ offering the least influence. Therefore, to effectively reduce malaria transmission within the human population, public health interventions should prioritize attaining bed net coverage through mass distribution campaigns and consistent bed net usage throughout the campaign period. Also, LLINs with higher efficacies towards pyrethroid-resistant vectors (for example, pyrethroid PBO and dual active ingredient nets) should be encouraged due to their higher effect on the $\mathcal{R}_0^L$ threshold.

4.2. Effect of Decreasing LLIN Retention on Malaria Dynamics

To investigate the effect of decreasing retention on malaria dynamics, three bed net campaigns were deployed for decreasing LLIN retention periods. From Figure (3), the proportion of susceptible humans fluctuates between 0.18 and 0.30 in the absence of bed nets, with respect to the seasonal pattern. This demonstrates a fluctuation in malaria infections as a response to seasonal variations in mosquito populations. At baseline, about 5% of the human population remains infectious over time. However, when LLINs are deployed, they substantially suppress malaria cases to zero, although these cases recover towards the end of the campaign as intervention effectiveness wanes to minimum levels.

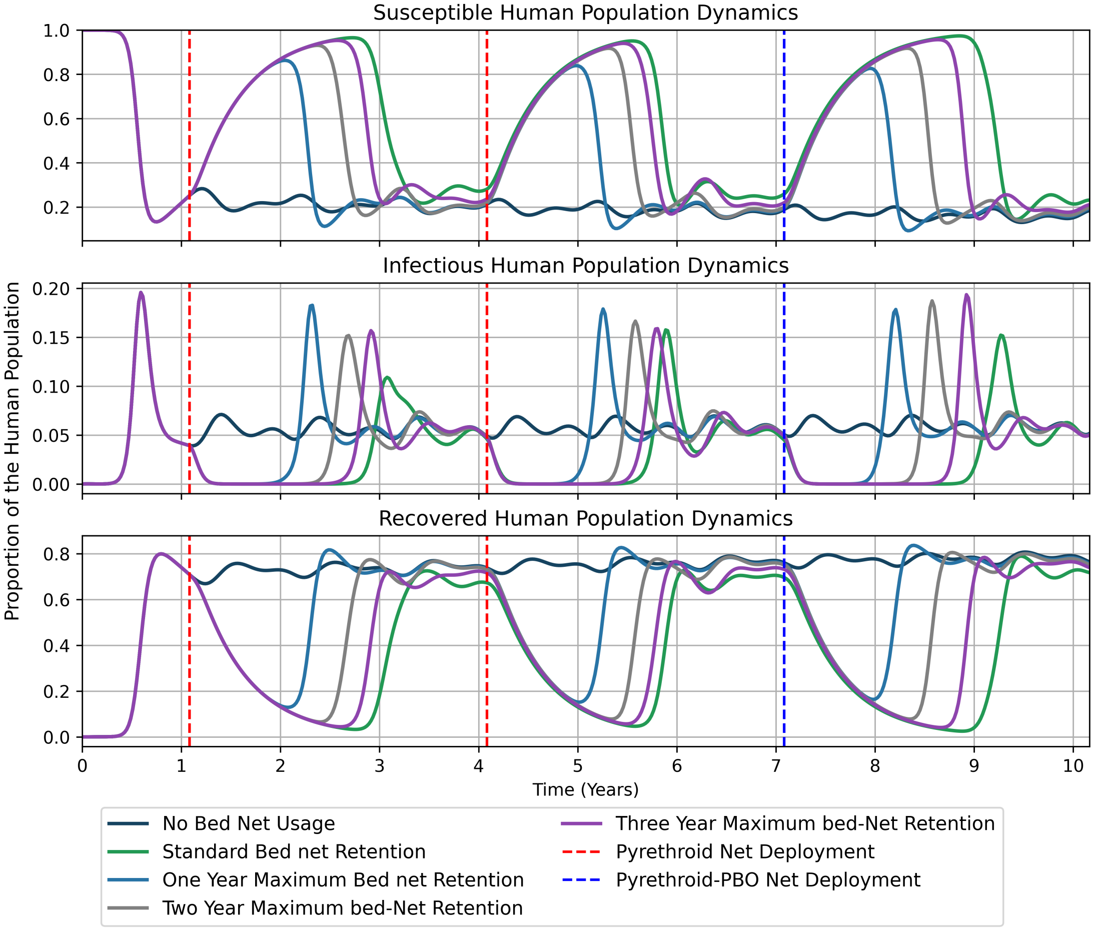

Figure 3: Effect of reducing LLIN retention periods on malaria dynamics across three bed net campaigns.

The standard bed net utilization offers the best malaria protection to humans as it suppresses infectious humans to negligible levels for a longer time of about two and a half years, before

their resurgence. The proportion of recovered humans decreases with the increase in averted malaria infections, for a given campaign period. However, the intervention effectiveness wanes over time and this proportion increases towards the baseline level as more people get infected with malaria.

To quantify the impact of LLIN campaigns across the four retention scenarios, the percentage of susceptible humans protected from malaria infection by bed nets is plotted in Figure (4). This shows that the standard bed net utilization had a better performance in averting

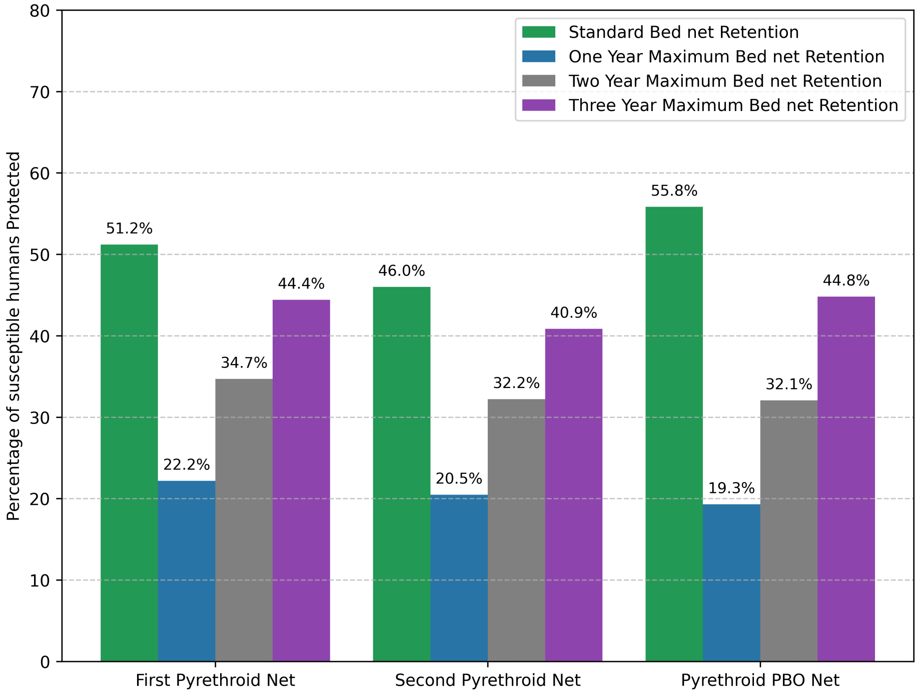

Figure 4: The percentage of susceptible humans protected from malaria infection due to use of LLINs in vector control.

malaria infections, protecting 51.2%, 46.0%, and 55.8% of the would-be malaria cases in the first, second, and third campaigns, respectively. Therefore, PBO LLINs avert more malaria infections due to their ability to kill both pyrethroid sensitive and resistant malaria vectors.

However, the one-year retention scenario performed worst in protecting susceptible humans due to its short duration in blocking and killing malaria vectors. Additionally, no advantage was registered from the use of pyrethroid PBO nets during the third campaign for the one and two-year maximum utilization periods. This is because such utilization levels do not provide

a sufficient killing force due to limited exposure periods towards malaria vectors, leading to low overall mosquito suppression impact and consequently low effectiveness levels.

4.3. Effect of Decreasing LLIN Retention on Mosquito Population Dynamics

This examined the effect of sequential LLIN campaigns on the epidemiological and total mosquito dynamics within seasonal settings.

4.3.1. LLIN Retention and Total Mosquito Population Dynamics

At baseline, the mosquito population stabilized after one year, following a bimodal seasonal profile with a primary mosquito population peak occurring in April and the a secondary, smaller peak in October [69, 70]. Figure (5) shows that standard bed net utilization has the greatest vector suppression effect across all scenarios. This suppresses the vector population to less than 2 million mosquitoes shortly after the pyrethroid-only deployment, but this effect reduces as bed net effectiveness wanes over time. The second deployment has a relatively reduced effect under some scenarios, but the overall dynamics tend towards baseline due to decay in bed net coverage and effectiveness.

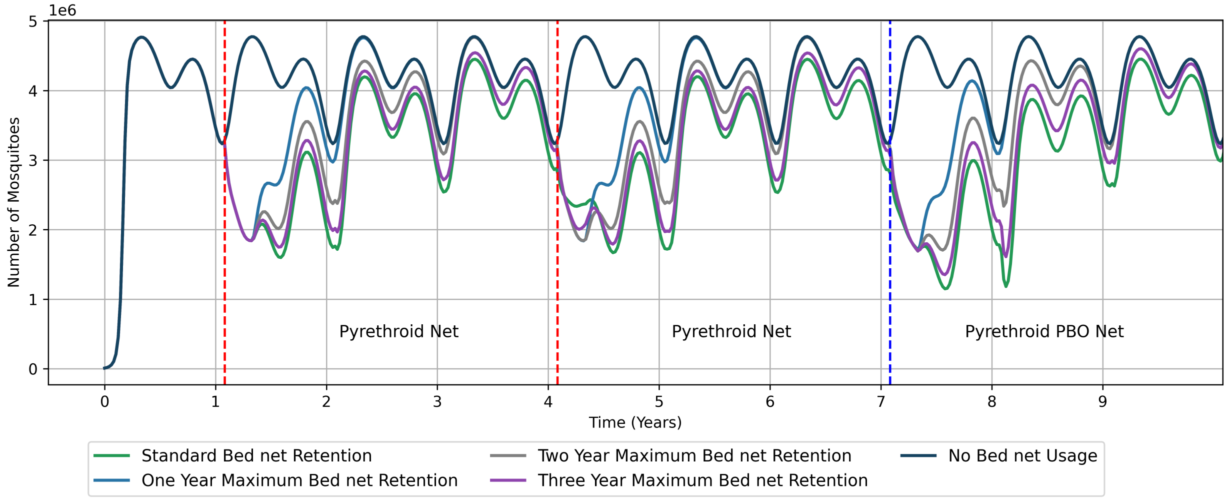

Figure 5: Impact of decreasing LLIN retention periods on mosquito population dynamics.

The third campaign demonstrates a greater suppression effect, reducing the mosquito population to about one million vectors within the first year of deployment, although this population recovers by the second year as the PBO synergist, coverage, and killing effectiveness decay.

4.3.2. LLIN Retention and Mosquito Epidemiological Dynamics

Figure (6) illustrates the impact of LLIN campaigns on pyrethroid-sensitive malaria vectors. Generally, LLIN deployments cause sudden reductions in the mosquito population, especially pyrethroid-sensitive vectors, but their numbers recover as the bed net effectiveness decays. Susceptible sensitive vectors maintained maximum levels when no bed net campaigns were deployed, stabilizing above 80% of the total mosquito population, while latently infected and infectious mosquitoes stabilized between 5% to 7.5%. Pyrethroid-only bed net cam-

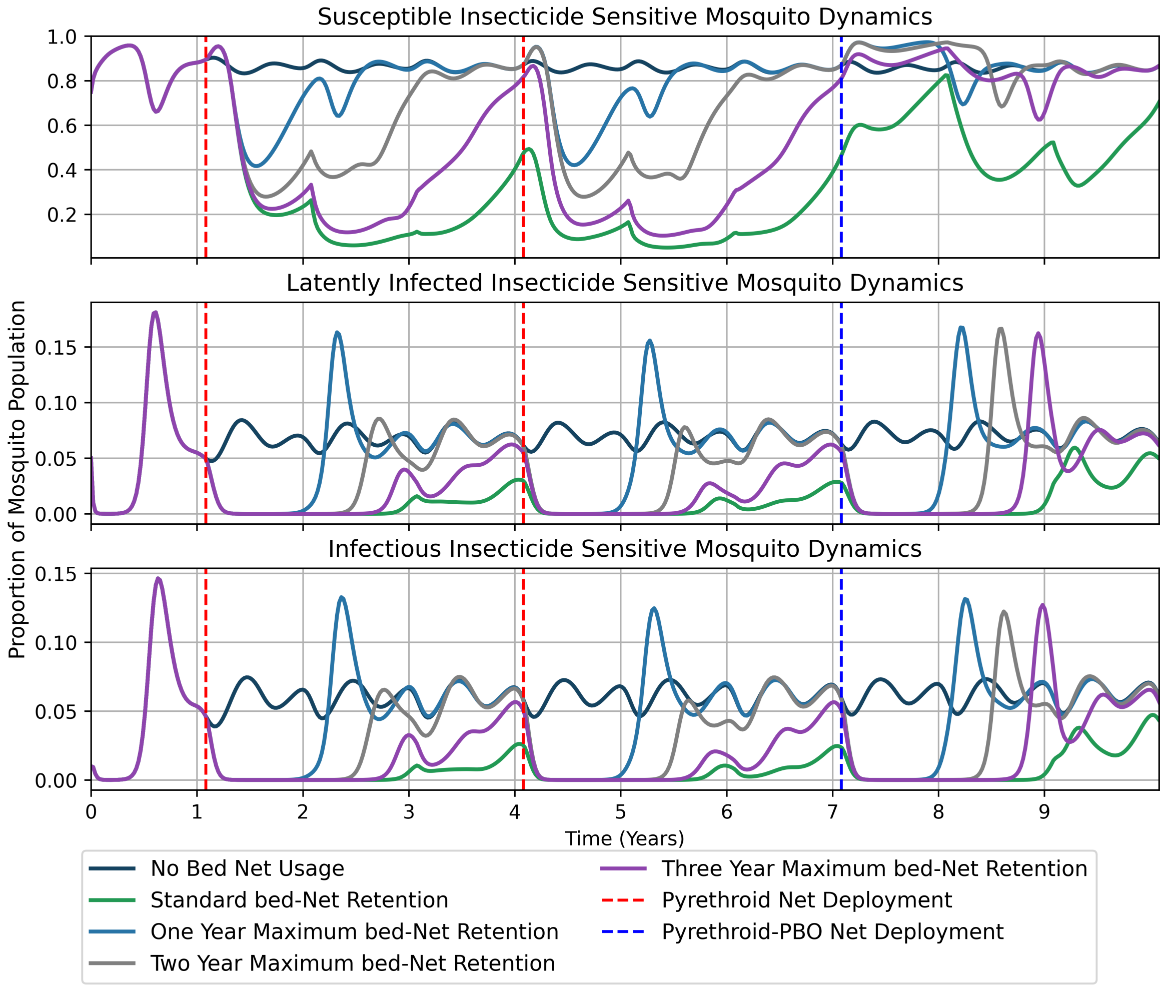

Figure 6: Effect of reduction in LLIN retention periods, across different deployment campaigns, on pyrethroid sensitive mosquito dynamics

paigns suppressed sensitive vectors to nearly zero for almost two years upon deployment under standard utilization settings, but the PBO nets showed less impact on susceptible pyrethroid-sensitive vectors. All campaigns demonstrate high effectiveness in suppressing

both latently infected and infectious malaria vectors, and PBO nets reduced these mosquitoes to near zero for the greater part of the campaign period. The one-year utilization scenario performed worst in reducing sensitive vectors, with the susceptible mosquitoes recovering towards baseline just after five months of deployment, and infected vectors recovering after one year into the campaign.

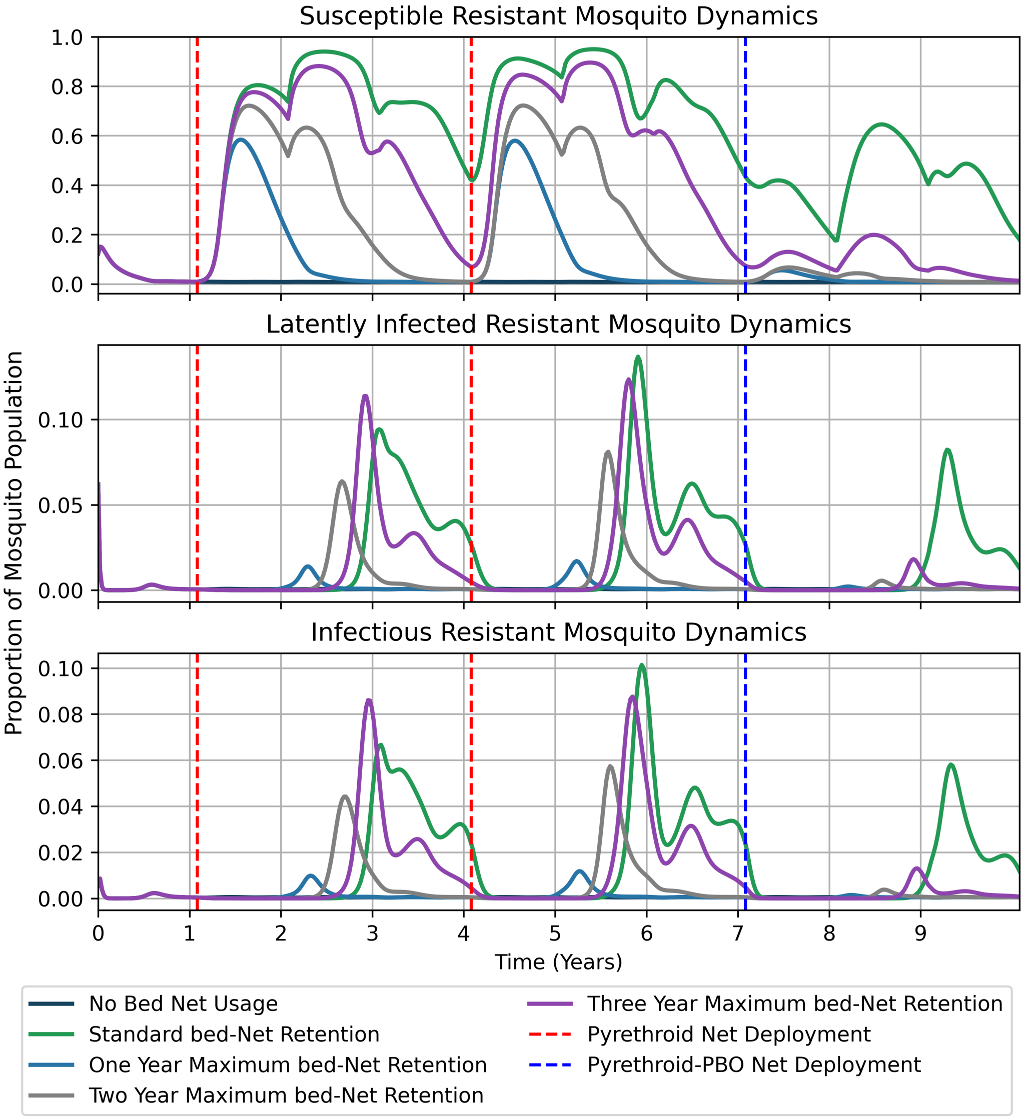

Figure 7: Effect of reduction in LLIN retention periods, across different deployment campaigns, on pyrethroid resistant mosquito dynamics

On the other hand, the proportion of pyrethroid-resistant vectors increased upon deployment of pyrethroid-only nets (7). This is because, as pyrethroid-sensitive mosquitoes are

suppressed, pyrethroid-resistant vectors grow in number, gradually becoming a substantial proportion of the mosquito population. Susceptible pyrethroid-resistant vectors increase at a faster rate than infected mosquitoes because newly emerging vectors are recruited into the susceptible class, thus taking some time before successfully blood feeding on infectious humans to acquire malaria parasites. The percentage of susceptible pyrethroid-resistant vectors increased to 90% of the mosquito population by the second year of the campaign, although it steadily declined towards the end of the campaign. The one-year utilization scenario indicated lower peaks and a faster reduction in resistant vectors, tending towards zero after about one and a half years. This is because such a level of usage applies selection pressure for a short period, insufficient to maintain resistant proportions throughout the entire campaign period. Therefore, across the four utilization settings, standard usage sustained resistant vectors for longer periods due to its higher selection for resistance. However, PBO nets displayed better suppression of resistant vectors across the four retention scenarios, although this effort reduced as the overall bed net killing effectiveness waned.

For pyrethroid-only campaigns, the proportion of infected resistant mosquitoes remains minimal during the initial phase of each campaign period, across the four utilization scenarios. This is because a mosquito must first survive a blood feeding attempt before it can become infected with malaria parasites. Immediately after LLIN deployment, strong blocking and killing effects substantially limit human-mosquito contact [71], while the number of infectious humans available for malaria transmission is low at this time [72]. However, as LLIN effectiveness gradually declines over time, more mosquitoes successfully blood feed. This effectiveness continues to decay further, and more sensitive mosquitoes survive the bed net intervention, causing an increase in their proportion, consequently reducing the fraction of resistant vectors.

4.4. Effect of Bed net Utilization and Fitness cost on the Evolution of Pyrethroid Resistance

The effect of bed net utilization and variations in fitness cost on the dynamics of pyrethroid resistance within the mosquito vectors, across the four bed net retention scenarios, was investigated in Figure (8) and Figure (9) respectively.

4.4.1. Effect of Bed net Utilization on the Evolution of Pyrethroid Resistance

Figure (8) shows the variations in sensitive, heterozygous and homozygous resistant genotype frequencies under three bed net campaigns, across different bed net utilization settings.

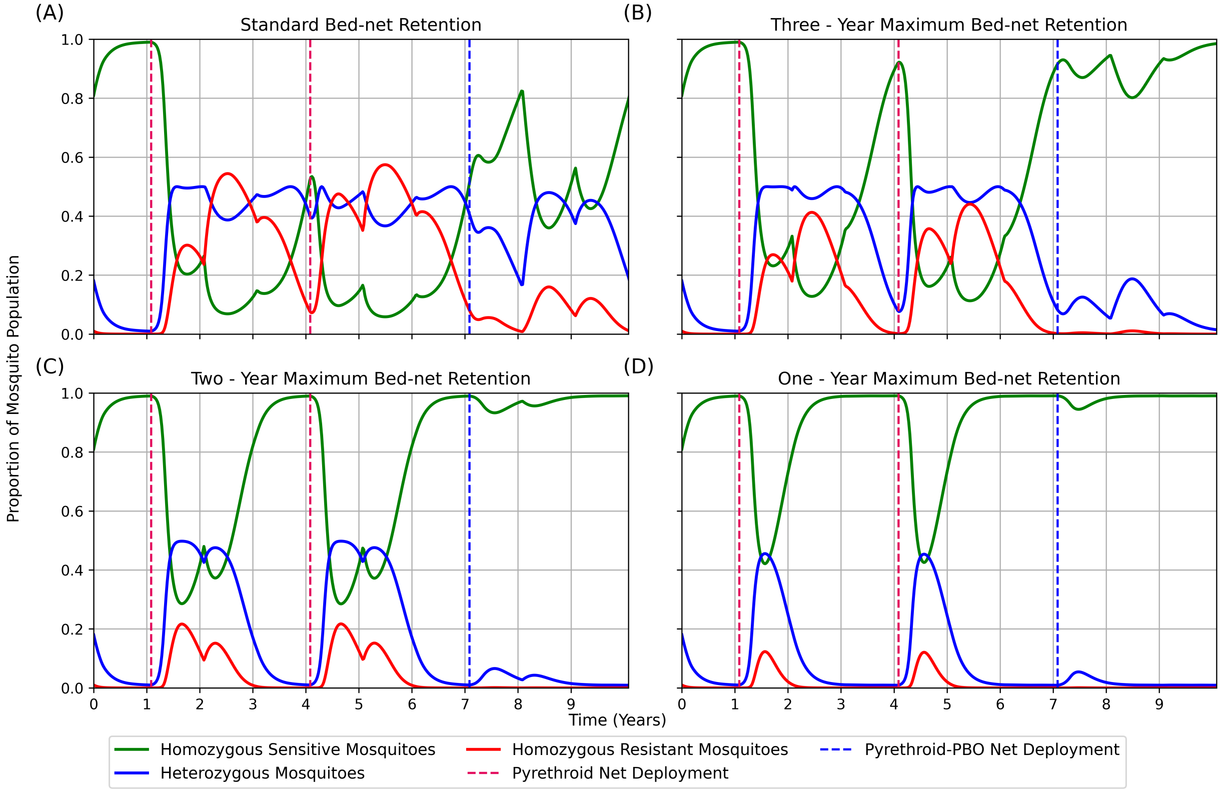

Figure 8: Impact of decreasing LLIN retention periods, across different deployment campaigns,on mosquito genotype frequencies

From panel A of Figure (8), the deployment of pyrethroid-only nets substantially reduces the proportion of sensitive vectors to a minimum of 0.1 after about sixteen months before gradually recovering as the bed net effectiveness wanes off. The proportion of resistant vectors consequently increases due to the intense selection pressure, with the heterozygous and homozygous resistant mosquitoes reaching a maximum of 0.5 in four months and 0.55 after sixteen months, respectively. Heterozygous mosquitoes rise at a faster rate when LLINs are deployed, compared to homozygous resistant vectors, due to their survival advantage and reproductive efficiency termed as ”heterozygous advantage”. However, as the selection pressure decays towards the end of the campaign, the percentage of homozygous resistant

vectors reduces to about 10% of the total population. When the selection pressure declines, the homozygous sensitive vectors continue to mate with homozygous resistant mosquitoes, replenishing the proportion of heterozygous vectors. Additionally, heterozygous mosquitoes suffer lower fitness costs, hence have better survival advantage when the bed net coverage and killing effectiveness decay, compared to homozygous resistant vectors. Therefore, the percentage of these mosquitoes suffers minimal suppression effects from homozygous sensitive vectors when the bed net coverage and killing effectiveness are reduced to minimum levels by the end of the campaign. The deployment of pyrethroid PBO nets reduces the emergence of homozygous resistant and heterozygous vectors, with the maximum proportions reaching 0.16 and 0.48 respectively. Therefore, PBO nets partially restored the mosquito pyrethroid sensitivity by suppressing the percentage of heterozygous and homozygous resistant mosquitoes to 20% and 2% respectively by the end of this campaign, and revamping the percentage of sensitive vectors up to 80% by the end of the campaign.

Panels C and D of Figure (8) demonstrate that the one-year and two-year bed net retention scenarios exert less selection pressure to amplify and maintain resistant genotypes across pyrethroid-only bed net campaigns. Following the deployment of LLINs, the frequency of pyrethroid-sensitive genotype decreased, while that for homozygous resistant and heterozygous genotypes increased to a maximum of 20% and 50% for two-year utilization, and 12% and 45% for a one-year retention scenario, respectively. When LLINs are utilized for a maximum of one or two years, the proportion of homozygous resistant vectors is restored to minimum frequencies before the end of each campaign. Therefore, these retention scenarios provide some selection for resistance, but they lack sufficient pressure to significantly increase and sustain resistance within the vector population.

4.4.2. Effect of Fitness Cost on the Evolution of Pyrethroid Resistance

In this subsection, different fitness cost values are used to understand their effect on the evolution of pyrethroid resistance among mosquito populations. Two kinds of fitness costs are considered, with one affecting mosquito recruitment (c) and the other reducing adult lifespan (w) of heterozygous and homozygous resistant vectors.

Figure 9 demonstrates that increasing fitness costs generally reduces the evolution of pyrethroid

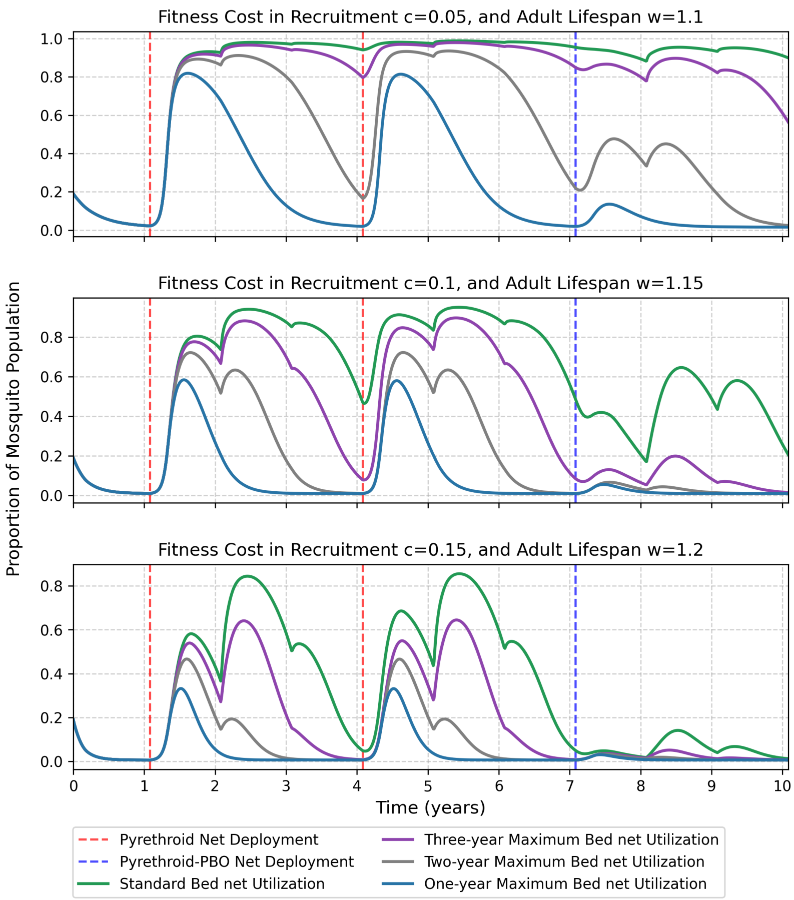

Figure 9: Effect of increasing fitness costs on the evolution of pyrethroid resistance within the mosquito population

resistance in the mosquito population. With a fitness cost of 5% reduction in resistant vector recruitment, and 10% decrease in adult lifespan of resistant mosquitoes, a higher selection force is observed. Under these conditions, the standard utilization scenario leads to near fixation of resistant genotypes by the end of the first and second pyrethroid-only campaigns. The PBO net deployment during the third campaign substantially reduced the percentage of resistant vectors for one and two-year utilization scenarios to 2%. However, longer retention periods showed less effect, reducing the percentage of resistant vectors to 95% and 58% for the standard and three-year utilization scenarios, respectively.

When these fitness costs in recruitment and adult lifespan increase to 10% and 15% respectively, resistance emerges at a reduced rate reaching a maximum of 94% in sixteen months, but this reduces to 48% by the end of the first campaign. During the second campaign,

a similar trend is observed. However, the deployment of pyrethroid PBO nets leads to a substantial reduction in resistance level, peaking at 62% before gradually declining to 20% at the end of the campaign period. This decline underscores the effectiveness of pyrethroid PBO LLINs in mitigating pyrethroid resistance, which is consistent with [73, 74]. Selection pressure for resistance reduces as utilization periods decrease, with the one and two-year retention scenarios showing no net increase in resistance from pyrethroid-only campaigns.

Higher fitness costs of 15% and 20% in mosquito recruitment and adult lifespan, respectively, substantially reduced the selection for resistance within and across pyrethroid-only campaigns. Under standard utilization, the percentage of resistant vectors increased to about 83% of the mosquito population after sixteen months from deployment, but this reduced to less than 10% by the end of this campaign. Shorter utilization periods show lower resistance peaks and overall selection force, thus registering negligible resistant percentages across pyrethroid-only campaigns. At these low levels of resistance, application of PBO nets suppressed the within-campaign resistance peaks and generally maintained the minimum proportions of resistant vectors. Therefore, the evolution of insecticide resistance is slowed by increasing fitness costs among vector populations [75]. Fixation of resistance genotype is less likely to occur when fitness costs are at least 15% and 20% for vector recruitment and adult lifespan, respectively. Under such conditions, a single pyrethroid PBO net campaign sufficiently suppresses resistant vectors to negligible proportions.

5. Discussion

This study formulates a deterministic mathematical model incorporating genotype-specific mosquito dynamics and human vector interactions. Malaria transmission reproduction numbers $\mathcal{R}_0$ and $\mathcal{R}_0^L$ with and without bed net usage respectively were computed. Sensitivity analysis on $\mathcal{R}_0^L$ showed that to effectively reduce malaria transmission, interventions promoting high bed net effective coverage and increased effectiveness of bed nets among pyrethroid-resistant vectors should be promoted for enhanced vector control.

Results show that an initial bed net coverage of 81%, without consistent bed net utilization, is insufficient in maintaining the protective effect of pyrethroid-only and pyrethroid-PBO

nets. Longer retention periods were found to be more effective across the different nets, but this reduces with decreasing utilization periods. Pyrethroid-PBO nets demonstrated higher effectiveness under standard utilization, increasing the number of averted infections from 46.0% to 55.8% of the human population, although the effect greatly deteriorates with shorter utilization periods. This highlights the importance of coverage and effective utilization on the effectiveness of LLIN campaigns, and how low utilisation levels can compromise the effectiveness of PBO nets (see also [76, 21, 20]).

The study notes that the effectiveness of LLINs measured by vector suppression, particularly pyrethroid-only and pyrethroid PBO nets, can be undermined by the declining coverage and inconsistent use. The entomological impact of both types of nets was substantially negated within 16 months due to the decline in effective coverage. A similar observation was made in a scientific research study [76], which showed that LLIN retention and usage significantly decline within 12 - 18 months of the campaign, undermining their long-term impact. Under standard utilization, PBO nets suppressed malaria vectors by about 72%, but pyrethroid-only had a smaller effect on this population.

The results also demonstrate that under standard utilization, successive pyrethroid-only campaigns amplify resistant mosquito genotypes, especially heterozygotes, but the deployment of PBO nets reverses this trend. Consistent with prior results [77, 78], pyrethroid-PBO nets offer a critical benefit in reversing insecticide resistance within the malaria vector population, although their effectiveness depends on sustained and consistent utilization. Further analysis shows that pyrethroid-PBO nets are more effective in mosquito populations where resistance to pyrethroid insecticides carries a higher fitness cost, regardless of the level of utilization. This is because higher fitness costs slow the evolution of pyrethroid resistance due to lower vector recruitment rates and survival advantage, a result in line with [73]. It is noted that the threat of pyrethroid resistance evolution is highest with high bed net utilization levels, but reducing this utilization is not a viable option in terms of malaria control. This provides a basis to rotate pyrethroid-only nets with LLINs enhanced with chlorfenapyr, pyriproxyfen, or PBO synergist, depending on the status of resistance, and complement these nets with other vector control tools like larval source management, IRS, house modifications, among

others.

Heterogeneity and localized transmission hotspots play an important role in malaria spread [79], hence the assumption that there is homogeneous mixing between human and mosquito populations limits the findings of this study. Public health bed net campaigns face delays, causing irregular deployment of nets [31], which impacts malaria dynamics. Therefore the assumption of regular replacement of bed nets after three years may not practically hold, especially in African settings. Pyrethroid resistance under field conditions is polygenic in nature [80] and fitness costs incurred by resistant vectors vary with seasonal conditions, genetic background, and differences in mosquito adaptive responses [81]. This study used a genetic structure represented by two alleles with fixed fitness costs, a limitation for its findings.

Acknowledgment

This study was supported by the African Consortium in Modelling for Effective Vector Control (ACoMVeC) project INV-047049. However, the sponsors did not have any role in the study design, choice of parametrization, analysis, or manuscript preparation and publication.

Authors to this study are grateful to anonymous reviewers for their important feedback that helped to improve and refine this work.

Conflict of interest

The authors to this manuscript declare no conflict of interest.

References

[1] World Health Organization, World malaria report 2024: Addressing inequity in the global malaria response, Licence: CC BY-NC-SA 3.0 IGO, World Health Organization, Geneva (2024).

[2] World Health Organization, World malaria day 2025: Malaria ends with us, published 25 April 2025. Statement by Dr Kasonde Mwinga, WHO Representative to Uganda

(2025).
URL https://www.afro.who.int/countries/uganda/news/world-malaria-day-2025-malaria-ends-us

[3] J. F. Namuganga, A. Epstein, J. I. Nankabirwa, A. Mpimbaza, M. Kiggundu, A. Sserwanga, J. Kapisi, The impact of stopping and starting indoor residual spraying on malaria burden in Uganda, Nature Communications 12 (1) (2021) 2635.

[4] G. Kombate, L. Djalogue, P. Ngangue, K. A.-M. Soubeiga, D. E. Grobbee, M. van der Sande, Integrated malaria vector control strategies and their effectiveness in sub-Saharan Africa: a systematic review protocol for interventional studies, BMJ open 15 (2) (2025) e091569.

[5] Q. Liu, Y. Wang, X. Liu, S. I. Hay, S. Lai, Stratified sustainable vector control strategies and measures for malaria control and elimination in China: a 70 year journey, BMJ 389 (2025).

[6] F. Okumu, S. J. Moore, P. Selvaraj, A. H. Yafin, E. O. Juma, G. G. Shirima, S. Majambere, A. Hardy, B. G. Knols, B. J. Msugupakulya, Elevating larval source management as a key strategy for controlling malaria and other vector-borne diseases in Africa, Parasites & Vectors 18 (1) (2025) 45.

[7] T. Fox, J. Furnival-Adams, M. Chaplin, M. Napier, E. A. Olanga, House modifications for preventing malaria, Cochrane Database of Systematic Reviews (10) (2022).

[8] G. Newby, P. Chaki, M. Latham, D. Marrenjo, E. Ochomo, D. Nimmo, E. Thomsen, A. Tatarsky, E. O. Juma, M. Macdonald, Larviciding for malaria control and elimination in Africa, Malaria Journal 24 (1) (2025) 16.

[9] Y. Zhou, W.-X. Zhang, E. Tembo, M.-Z. Xie, S.-S. Zhang, X.-R. Wang, T.-T. Wei, X. Feng, Y.-L. Zhang, J. Du, Effectiveness of indoor residual spraying on malaria control: a systematic review and meta-analysis, Infectious Diseases of Poverty 11 (04) (2022) 29–42.

[10] S. Bhatt, D. Weiss, E. Cameron, D. Bisanzio, B. Mappin, U. Dalrymple, K. Battle, The effect of malaria control on Plasmodium falciparum in Africa between 2000 and 2015, Nature 526 (7572) (2015) 207–211.

[11] B. B. Tukei, A. Beke, H. Lamadrid-Figueroa, Assessing the effect of indoor residual spraying (IRS) on malaria morbidity in Northern Uganda: a before and after study, Malaria Journal 16 (2017) 1–9.

[12] H. Ranson, Current and future prospects for preventing malaria transmission via the use of insecticides, Cold Spring Harbor Perspectives in Medicine 7 (11) (2017) a026823.

[13] S. W. Lindsay, M. B. Thomas, I. Kleinschmidt, Threats to the effectiveness of insecticide-treated bednets for malaria control: thinking beyond insecticide resistance, The Lancet Global Health 9 (9) (2021) e1325–e1331.

[14] A. Oruni, M. Tchouakui, C. S. D. Tagne, J. Hearn, J. Kayondo, C. S. Wondji, Temporal evolution of insecticide resistance and bionomics in Anopheles funestus, a key malaria vector in Uganda, Scientific Reports 14 (1) (2024) 32027.

[15] Y. Akuamoah-Boateng, R. C. Brenyah, S. A. Kwarteng, P. Obuam, I. Owusu-Frimpong, A. K. Agyapong, K. Badu, Malaria transmission, vector diversity, and insecticide resistance at a peri-urban site in the forest zone of Ghana, Frontiers in Tropical Diseases 2 (2021) 739771.

[16] B. D. Menze, M. Tchouakui, L. M. Mugenzi, W. Tchapga, M. Tchoupo, M. J. Wondji, M. Chiumia, T. Mzilahowa, C. S. Wondji, Marked aggravation of pyrethroid resistance in major malaria vectors in Malawi between 2014 and 2021 is partly linked with increased expression of P450 alleles, BMC Infectious Diseases 22 (1) (2022) 660.

[17] P. W. Orondo, S. G. Nyanjom, H. Atieli, J. Githure, B. M. Ondeto, K. O. Ochwedo, C. J. Omondi, J. W. Kazura, M.-C. Lee, G. Zhou, Insecticide resistance status of Anopheles arabiensis in irrigated and non-irrigated areas in western Kenya, Parasites & Vectors 14 (1) (2021) 335.

[18] World Health Organization, Guidance on the prioritization of insecticide-treated nets in situations where resources are limited, iSBN 978-92-4-006942-8 (electronic), ISBN 978-92-4-006943-5 (print), CC BY-NC-SA 3.0 IGO licence (2023). URL https://iris.who.int/bitstream/handle/10665/366406/9789240069428-eng.pdf?sequence=1

[19] World Health Organization, Who guidelines for malaria, 16 october 2023 (2023). URL https://iris.who.int/bitstream/handle/10665/373339/WHO-UCN-GMP-2023.01-Rev.1-eng.pdf

[20] A. Kwiringira, C. Nanziri, S. M. Migamba, S. R. Ahirirwe, D. Kadobera, A. R. Ario, Ownership and Use of Long-lasting Insecticidal Nets and Factors Associated, Immediately after a Mass Distribution Campaign in Uganda: A Cross-sectional Survey of Fourteen districts (2021).

[21] J. Okiring, S. Gonahasa, M. Nassali, J. F. Namuganga, I. Bagala, C. Maiteki-Sebuguzi, J. Opigo, I. Nabende, J. Nangendo, J. Kabami, LLIN Evaluation in Uganda Project (LLINEUP2)—Factors associated with coverage and use of long-lasting insecticidal nets following the 2020–21 national mass distribution campaign: a cross-sectional survey of 12 districts, Malaria Journal 21 (1) (2022) 293.

[22] T. F. Seyoum, Z. Andualem, H. F. Yalew, Insecticide-treated bed net use and associated factors among households having under-five children in East Africa: a multilevel binary logistic regression analysis, Malaria Journal 22 (1) (2023) 10.

[23] M. Kanyangarara, H. Hamapumbu, E. Mamini, J. Lupiya, J. C. Stevenson, S. Mharakurwa, M. Chaponda, P. E. Thuma, L. Gwanzura, S. Munyati, Malaria knowledge and bed net use in three transmission settings in southern Africa, Malaria Journal 17 (2018) 1–12.

[24] A. Bertozzi-Villa, C. A. Bever, H. Koenker, D. J. Weiss, C. Vargas-Ruiz, A. K. Nandi, H. S. Gibson, J. Harris, K. E. Battle, S. F. Rumisha, Maps and metrics of insecticide-treated net access, use, and nets-per-capita in Africa from 2000-2020, Nature Communications 12 (1) (2021) 3589.

[25] I. Enahoro, S. Eikenberry, A. B. Gumel, S. Huijben, K. Paaijmans, Long-lasting insecticidal nets and the quest for malaria eradication: a mathematical modeling approach, Journal of Mathematical Biology 81 (1) (2020) 113–158.

[26] P. L. Birget, J. C. Koella, An epidemiological model of the effects of insecticide-treated bed nets on malaria transmission, PloS one 10 (12) (2015) e0144173.

[27] F. O. Okumu, S. S. Kiware, S. J. Moore, G. F. Killeen, Mathematical evaluation of community level impact of combining bed nets and indoor residual spraying upon malaria transmission in areas where the main vectors are anopheles arabiensis mosquitoes, Parasites & Vectors 6 (2013) 1–13.

[28] B. Levick, A. South, I. M. Hastings, A two-locus model of the evolution of insecticide resistance to inform and optimise public health insecticide deployment strategies, PLoS Computational Biology 13 (1) (2017) e1005327.

[29] S. Barbosa, K. Kay, N. Chitnis, I. M. Hastings, Modelling the impact of insecticide-based control interventions on the evolution of insecticide resistance and disease transmission, Parasites & Vectors 11 (2018) 1–21.

[30] A. A. Msola, Mathematical modelling of malaria based on uganda data (2018).

[31] J. Okiring, S. Gonahasa, C. Maiteki-Sebuguzi, A. Katureebe, I. Bagala, P. Mutungi, S. P. Kigozi, J. F. Namuganga, J. I. Nankabirwa, M. R. Kamya, LLIN Evaluation in Uganda Project (LLINEUP): modelling the impact of COVID-19-related disruptions on delivery of long-lasting insecticidal nets on malaria indicators in Uganda, Malaria Journal 23 (1) (2024) 180.

[32] T. S. Churcher, N. Lissenden, J. T. Griffin, E. Worrall, H. Ranson, The impact of pyrethroid resistance on the efficacy and effectiveness of bednets for malaria control in Africa, Elife 5 (2016) e16090.

[33] J. Mohammed-Awel, A. B. Gumel, Mathematics of an epidemiology-genetics model for assessing the role of insecticides resistance on malaria transmission dynamics, Mathematical Biosciences 312 (2019) 33–49.

[34] National Human Genome Research Institute (NHGRI), Allele, https://www.genome.gov/genetics-glossary/Allele, accessed: 2025-08-11 (2024).

[35] Study.com, Genotype definition, types & examples, accessed: 2025-08-11 (2024). URL https://study.com/academy/lesson/what-are-genotypes-definition-examples-quiz.html

[36] M. L. G. Kuniyoshi, F. L. P. d. Santos, Mathematical modelling of vector-borne diseases and insecticide resistance evolution, Journal of Venomous Animals and Toxins including Tropical Diseases 23 (2018).

[37] Yang, M. Macoris, K. Galvani, M. Andrighetti, D. Wanderley, Assessing the effects of temperature on the population of Aedes aegypti, the vector of dengue, Epidemiology & Infection 137 (8) (2009) 1188–1202.

[38] P. van den Driessche, J. Watmough, Reproduction numbers and sub-threshold endemic equilibria for compartmental models of disease transmission, Mathematical Biosciences 180 (1) (2002) 29–48.

[39] A. Perasso, An introduction to the basic reproduction number in mathematical epidemiology, ESAIM: Proceedings and Surveys 62 (2018) 123–138.

[40] B. D. Menze, M. F. Kouamo, M. J. Wondji, W. Tchapga, M. Tchoupo, M. O. Kusimo, C. S. Mouhamadou, J. M. Riveron, C. S. Wondji, An experimental hut evaluation of PBO-based and pyrethroid-only nets against the malaria vector Anopheles funestus reveals a loss of bed nets efficacy associated with GSTe2 metabolic resistance, Genes 11 (2) (2020) 143.

[41] R. N'Guessan, A. Asidi, P. Boko, A. Odjo, M. Akogbeto, O. Pigeon, M. Rowland, An experimental hut evaluation of PermaNet® 3.0, a deltamethrin—piperonyl butoxide combination net, against pyrethroid-resistant Anopheles gambiae and Culex quinquefasciatus mosquitoes in southern Benin, Transactions of the Royal Society of Tropical Medicine and Hygiene 104 (12) (2010) 758–765.

[42] F. Mechan, A. Katureebe, V. Tuhaise, M. Mugote, A. Oruni, I. Onyige, K. Bumali, J. Thornton, K. Maxwell, M. Kyohere, LLIN Evaluation in Uganda Project (LLINEUP)–The durability of long-lasting insecticidal nets treated with and without piperonyl butoxide (PBO) in Uganda, BioRxiv (2022) 2022–02.

[43] J. Baafi, A. Hurford, Modeling the impact of seasonality on mosquito population dynamics: Insights for vector control strategies, Bulletin of Mathematical Biology 87 (2) (2025) 33.

[44] D. Z. Childs, M. Boots, The interaction of seasonal forcing and immunity and the resonance dynamics of malaria, Journal of the Royal Society Interface 7 (43) (2010) 309–319.

[45] D. L. Nkahe, E. Kopya, B. Djiappi-Tchamen, W. Toussile, N. Sonhafouo-Chiana, S. Kekeunou, R. Mimpfoundi, P. Awono-Ambene, C. S. Wondji, C. Antonio-Nkondjio, Fitness cost of insecticide resistance on the life-traits of a anopheles coluzzii population from the city of yaoundé, cameroon, Wellcome Open Research 5 (2020) 171.

[46] J. Mohammed-Awel, A. B. Gumel, A genetic-epidemiology modeling framework for malaria mosquitoes and disease, Mathematical and computational modeling of phenomena arising in population biology and nonlinear oscillations. Contemporary mathematics 793 (2024).

[47] D. Otali, R. J. Novak, W. Wan, S. Bu, D. R. Moellering, M. De Luca, Increased production of mitochondrial reactive oxygen species and reduced adult life span in an insecticide-resistant strain of anopheles gambiae, Bulletin of entomological research 104 (3) (2014) 323–333.

[48] G. Msangi, M. I. Olotu, A. M. Mahande, A. Philbert, E. J. Kweka, The Impact of Insecticide Pre-Exposure on Longevity, Feeding Succession, and Egg Batch Size of Wild Anopheles gambiae sl, Journal of Tropical Medicine 2020 (1) (2020) 8017187.

[49] J. K. Osoro, M. G. Machani, E. Ochomo, C. Wanjala, E. Omukunda, S. Munga, A. K. Githeko, G. Yan, Y. A. Afrane, Insecticide resistance exerts significant fitness costs in

immature stages of Anopheles gambiae in western Kenya, Malaria journal 20 (1) (2021) 259.

[50] C. N. Ngonghala, J. Wairimu, J. Adamski, H. Desai, Impact of adaptive mosquito behavior and insecticide-treated nets on malaria prevalence, Journal of Biological Systems 28 (02) (2020) 515–542.

[51] A. Rivero, A. Magaud, A. Nicot, J. Vézilier, Energetic cost of insecticide resistance in culex pipiens mosquitoes, Journal of medical entomology 48 (3) (2011) 694–700.

[52] T. S. Churcher, R. E. Sinden, N. J. Edwards, I. D. Poulton, T. W. Rampling, P. M. Brock, J. T. Griffin, L. M. Upton, S. E. Zakutansky, K. A. Sala, Probability of transmission of malaria from mosquito to human is regulated by mosquito parasite density in naive and vaccinated hosts, PLoS Pathogens 13 (1) (2017) e1006108.

[53] T. S. Churcher, T. Bousema, M. Walker, C. Drakeley, P. Schneider, A. L. Ouédraogo, M.-G. Basáñez, Predicting mosquito infection from plasmodium falciparum gametocyte density and estimating the reservoir of infection, Elife 2 (2013) e00626.

[54] J. Bradley, W. Stone, D. F. Da, I. Morlais, A. Dicko, A. Cohuet, W. M. Guelbeogo, A. Mahamar, S. Nsango, H. M. Soumaré, Predicting the likelihood and intensity of mosquito infection from sex specific plasmodium falciparum gametocyte density, Elife 7 (2018) e34463.

[55] J. Olum, D. Mukunya, B. Nambozo, R. Nantale, F. Oguttu, J. Epuitai, I. Lume, B. Wanume, P. Olupot-Olupot, D. Amanya, Severe malaria re-admissions in Northern Uganda: a cross-sectional study, Malaria Journal 24 (1) (2025) 64.

[56] A. K. Musiime, D. L. Smith, M. Kilama, J. Rek, E. Arinaitwe, J. I. Nankabirwa, M. R. Kamya, M. D. Conrad, G. Dorsey, A. M. Akol, Impact of vector control interventions on malaria transmission intensity, outdoor vector biting rates and Anopheles mosquito species composition in Tororo, Uganda, Malaria Journal 18 (2019) 1–9.

[57] Uganda Bureau of Statistics 2024, The National Population and Housing Census 2024 – Final Report.

[58] W. Mamai, N. S. Bimbile-Somda, H. Maiga, J. G. Juarez, Z. A. Muosa, A. B. Ali, R. S. Lees, J. R. Gilles, Optimization of mosquito egg production under mass rearing setting: effects of cage volume, blood meal source and adult population density for the malaria vector, Anopheles arabiensis, Malaria Journal 16 (1) (2017) 41.

[59] J. Matthews, A. Bethel, G. Osei, An overview of malarial anopheles mosquito survival estimates in relation to methodology, Parasites & vectors 13 (1) (2020) 233.

[60] J. R. Balentine, C. P. Davis, How long will it take to recover from malaria?, last Editorial Review: October 3, 2018. MedicineNet. Accessed April 24, 2025 (2018). URL https://www.medicinenet.com/how_long_will_it_take_to_recover_from_malaria/ask.htm

[61] E. Guissou, D. F. Da, D. F. d. S. Hien, K. B. Yameogo, S. R. Yerbanga, G. A. Ouédraogo, K. R. Dabiré, T. Lefèvre, A. Cohuet, Intervention reducing malaria parasite load in vector mosquitoes: No impact on Plasmodium falciparum extrinsic incubation period and the survival of Anopheles gambiae, PloS Pathogens 19 (5) (2023) e1011084.

[62] Target Malaria, Uganda Virtual Press Kit, Tech. rep., Uganda Virus Research Institute, [online; accessed 14/01/2025] (January 2025).

[63] W. Takken, D. Charlwood, S. W. Lindsay, The behaviour of adult Anopheles gambiae, sub-Saharan Africa's principal malaria vector, and its relevance to malaria control: a review, Malaria Journal 23 (1) (2024) 161.

[64] K. Gleave, A. Guy, F. Mechan, M. Emery, A. Murphy, V. Voloshin, C. E. Towers, D. Towers, H. Ranson, G. M. Foster, Impacts of dual active-ingredient bed nets on the behavioural responses of pyrethroid resistant Anopheles gambiae determined by room-scale infrared video tracking, Malaria Journal 22 (1) (2023) 132.

[65] M. Okia, R. Ndyomugyenyi, J. Kirunda, A. Byaruhanga, S. Adibaku, D. K. Lwamafa, F. Kironde, Bioefficacy of long-lasting insecticidal nets against pyrethroid-resistant populations of Anopheles gambiae ss from different malaria transmission zones in Uganda, Parasites & Vectors 6 (2013) 1–10.

[66] P. M. Sovegnon, R. Akoton, I. J. Stopard, T. S. Churcher, P. J. McCall, H. Ranson, G. M. Foster, L. S. Djogbénou, Efficacy of Interceptor G2, Royal Guard and PermaNet 3.0 against pyrethroid-resistant Anopheles gambiae sl from Za-Kpota, southern Benin: an experimental hut trial, Parasites & Vectors 17 (1) (2024) 300.

[67] Impact of different mosquito collection methods on indicators of anopheles malaria vectors in uganda.

[68] S. J. Brozak, J. Mohammed-Awel, A. B. Gumel, Mathematics of a single-locus model for assessing the impacts of pyrethroid resistance and temperature on population abundance of malaria mosquitoes, Infectious Disease Modelling 7 (3) (2022) 277–316.

[69] J. Majaliwa, M. Tenywa, D. Bamanya, W. Majugu, P. Isabirye, C. Nandozi, J. Nampijja, P. Musinguzi, A. Nimusiima, K. Luswata, Characterization of historical seasonal and annual rainfall and temperature trends in selected climatological homogenous rainfall zones of Uganda, Global Journal of Science Research 15 (4) (2015) 21–40.

[70] M. R. Jury, Uganda rainfall variability and prediction, Theoretical and Applied Climatology 132 (2018) 905–919.

[71] K. P. Paaijmans, S. Huijben, Taking the 'i'out of llins: using insecticides in vector control tools other than long-lasting nets to fight malaria, Malaria Journal 19 (1) (2020) 73.

[72] A. Epstein, S. Gonahasa, J. F. Namuganga, M. J. Nassali, C. Maiteki-Sebuguzi, I. Nabende, K. Snyman, J. I. Nankabirwa, J. Opigo, M. J. Donnelly, et al., Evaluating the impact of two next-generation long-lasting insecticidal nets on malaria incidence in Uganda: an interrupted time-series analysis using routine health facility data, BMJ Global Health 10 (3) (2025).

[73] S. Gonahasa, J. F. Namuganga, M. J. Nassali, C. Maiteki-Sebuguzi, I. Nabende, A. Epstein, K. Snyman, J. I. Nankabirwa, J. Opigo, M. J. Donnelly, LLIN Evaluation in Uganda Project (LLINEUP2)–Effect of long-lasting insecticidal nets (LLINs) treated with pyrethroid plus pyriproxyfen vs LLINs treated with pyrethroid plus piperonyl bu-

toxide in Uganda: A cluster-randomised trial, PLOS Global Public Health 5 (2) (2025) e0003558.

[74] N. Protopopoff, J. F. Mosha, L. A. Messenger, E. Lukole, J. D. Charlwood, A. Wright, E. Kessy, A. Manjurano, F. W. Mosha, I. Kleinschmidt, Effectiveness of piperonyl butoxide and pyrethroid-treated long-lasting insecticidal nets (LLINs) versus pyrethroid-only LLINs with and without indoor residual spray against malaria infection: third year results of a cluster, randomised controlled, two-by-two factorial design trial in Tanzania, Malaria Journal 22 (1) (2023) 294.

[75] L. M. Rigby, G. Rašić, C. L. Peatey, L. E. Hugo, N. W. Beebe, G. J. Devine, Identifying the fitness costs of a pyrethroid-resistant genotype in the major arboviral vector aedes aegypti, Parasites & Vectors 13 (1) (2020) 358.

[76] J. Okiring, P. Katamba, C. Kamya, F. Namugaya, G. Lavoy, G. de Broucker, A. Ssebagereka, E. E. Kiracho, J. Mak, B. N. Patenaude, Inequity in the use of long-lasting insecticidal nets before and after nationwide mass distribution campaign in 48 districts of uganda: Verse toolkit analysis, BMC Public Health 25 (1) (2025) 1–11.

[77] N. Protopopoff, J. F. Mosha, E. Lukole, J. D. Charlwood, A. Wright, C. D. Mwalimu, A. Manjurano, F. W. Mosha, W. Kisinza, I. Kleinschmidt, et al., Effectiveness of a long-lasting piperonyl butoxide-treated insecticidal net and indoor residual spray interventions, separately and together, against malaria transmitted by pyrethroid-resistant mosquitoes: a cluster, randomised controlled, two-by-two factorial design trial, The Lancet 391 (10130) (2018) 1577–1588.

[78] M. Chaplin, L. Choi, H. Ranson, Piperonyl butoxide (PBO) combined with pyrethroids in insecticide-treated nets to prevent malaria in Africa, Cochrane Database of Systematic Reviews (5) (2021).

[79] S. Mandal, R. R. Sarkar, S. Sinha, Mathematical models of malaria-a review, Malaria journal 10 (1) (2011) 202.

[80] J. M. Riveron, S. Huijben, W. Tchapga, M. Tchouakui, M. J. Wondji, M. Tchoupo, H. Irving, N. Cuamba, M. Maquina, K. Paaijmans, et al., Escalation of pyrethroid resistance in the malaria vector anopheles funestus induces a loss of efficacy of piperonyl butoxide–based insecticide-treated nets in mozambique, The Journal of infectious diseases 220 (3) (2019) 467–475.

[81] T. Bodineau, P. de Villemereuil, S. Agostini, B. Decencière, J.-F. Le Galliard, S. Meylan, Breeding phenology drives variation in reproductive output, reproductive costs, and offspring fitness in a viviparous ectotherm, Journal of Evolutionary Biology 37 (9) (2024) 1023–1034.

6. Appendices

6.0.1. Positivity, well-posedness and Boundedness

To fully represent the model system (2.2), the initial conditions of the form $\mathcal{D}(0) = (S_h(0), I_h(0), R_h(0), S_{SS}(0), E_{SS}(0), I_{SS}(0), S_{SR}(0), E_{SR}(0), I_{SR}(0), S_{RR}(0), E_{RR}(0), I_{RR}(0))$, where $N_h(0) = S_h(0) + I_h(0) + R_h(0)$ and $N_v = S_{SS}(0) + E_{SS}(0) + I_{SS} + S_{SR}(0) + E_{SR}(0) + I_{SR} + S_{RR}(0) + E_{RR}(0) + I_{RR}$ need to be within an epidemiologically feasible region. The state variables $S_h, I_h, R_h, S_{SS}, E_{SS}, I_{SS}, S_{SR}, E_{SR}, I_{SR}, S_{RR}, E_{RR}$ and I_{RR} and their corresponding initial conditions in $\mathcal{D}(0)$ are positive since they represent human and mosquito populations. Therefore, for initial conditions of the form $\mathcal{D}(0)$ in the epidemiologically feasible region,

$$\xi = \Big\{(S_h, I_h, R_h, S_{SS}, E_{SS}, I_{SS}, S_{SR}, E_{SR}, I_{SR}, S_{RR}, E_{RR}, I_{RR}) \in \mathbb{R}^{12}_{+} : 0 < S_h + I_h + R_h < N_h(0) \exp^{(\Lambda_h - \mu_h)t}, 0 < S_{SS} + E_{SS} + I_{SS} + S_{SR} + E_{SR} + I_{SR} + S_{RR} + E_{RR} + I_{RR} < \frac{K N_v(0)}{N_v(0) + (K - N_v(0)) \exp^{-\Lambda_v t}}\Big\}$$

The human population, from system (2.2), is such that $\frac{dN_h}{dt} = \Lambda_h - \mu_h N_h - \gamma I_h \leq \Lambda_h - \mu_h N_h$. This follows that $N_h(t) \leq \frac{\Lambda_h}{\mu_h} + \left(N_h(0) - \frac{\Lambda_h}{\mu_h}\right) \exp^{-\mu_h t}$ where $N_h(0)$ is the total number of people at time $t = 0$. Similarly, the total population of mosquitoes is given by the equation $\frac{dN_v}{dt} = \Lambda_v N_v \left(1 - \frac{N_v}{K}\right) - (m\sigma_s + \mu_{vs}) N_{SS} - (m\sigma_r + \mu_{vr})(N_{SR} + N_{RR}) \leq \Lambda_v N_v \left(1 - \frac{N_v}{K}\right)$.

It follows that $N_v(t) \leq \dfrac{KN_v(0)}{N_v(0) + (K - N_v(0))\exp^{-\lambda_v t}}$ for all $t \geq 0$, where $N_v(0)$ is the initial vector population. Therefore, as time $t \to \infty$;

1. The $\lim_{t\to\infty} N_h(t) \leq \dfrac{\Lambda_h}{\mu_h}$, thus the total human population converges to the steady state value $\dfrac{\Lambda_h}{\mu_h}$.
2. Similarly, $\lim_{t\to\infty} N_v(t) \leq \dfrac{KN_v(0)}{N_v(0)} = K$. Thus, the total mosquito population approaches the carrying capacity from below implying that this vector population will saturate around K in the long-run.

The epidemiologically feasible region $\xi = \Bigg\{(S_h, I_h, R_h, S_{SS}, E_{SS}, I_{SS}, S_{SR}, E_{SR}, I_{SR}, S_{RR}, E_{RR}, I_{RR}) \in \mathbb{R}^{12}_{+} : 0 < S_h + I_h + R_h < \dfrac{\Lambda_h}{\mu_h} + \left(N_h(0) - \dfrac{\Lambda_h}{\mu_h}\right)\exp^{-\mu_h t}, 0 < S_{SS} + E_{SS} + I_{SS} + S_{SR} + E_{SR} + I_{SR} + S_{RR} + E_{RR} + I_{RR} < \dfrac{KN_v(0)}{N_v(0) + (K - N_v(0))\exp^{-\Lambda_v t}}\Bigg\}$ is positively invariant and attracting for all $t \geq 0$ with respect to the model system (2.2).

Lemma 1. *The solutions to system* (2.2) *are positive, indicating that region* $\mathbb{R}^{12}_{+}$ *is positively invariant.*

Proof. Suppose there exists t_1 such that $S_h(t_1) = 0$, $S_h(t_1)' \leq 0$ and $S_h(t) > 0$, $I_h(t) > 0$, $R_h(t) > 0$, $S_{SS}(t) > 0$, $E_{SS}(t) > 0$, $I_{SS}(t) > 0$, $S_{SR}(t) > 0$, $E_{SR}(t) > 0$, $I_{SR}(t) > 0$, $S_{RR}(t) > 0$, $E_{RR}(t) > 0$, $I_{RR}(t) > 0$, for $0 < t < t_1$. Thus,

$$S_h'(t_1) = \Lambda_h + \vartheta R_h - \frac{\tau_h(I_{SS}(t_1) + I_{SR}(t_1) + I_{RR}(t_1))}{N_h}\big(m\beta_{bloc}\rho_{in} + (1-m)\rho_{out}\big)S_h(t_1) - \mu_h S_h(t_1) > 0, \tag{6.1}$$

which contradicts with $S_h(t_1)' \leq 0$. Therefore, t_1 does not exist. Further more, suppose t_2 exists such that $I_h(t_2) = 0$, $I_h(t_2)' \leq 0$ and $S_h(t) > 0$, $I_h(t) > 0$, $R_h(t) > 0$, $S_{SS}(t) > 0$, $E_{SS}(t) > 0$, $I_{SS}(t) > 0$, $S_{SR}(t) > 0$, $E_{SR}(t) > 0$, $I_{SR}(t) > 0$, $S_{RR}(t) > 0$, $E_{RR}(t) > 0$, $I_{RR}(t) > 0$, for $0 < t < t_2$. It follows that:

$$I_h'(t_2) = \frac{\tau_h(I_{SS}(t_2) + I_{SR}(t_2) + I_{RR}(t_2))S_h}{N_h}\big(m\beta_{bloc}\rho_{in} + (1-m)\rho_{out}\big) - \varphi I_h(t_2) - (\mu_h + \gamma)I_h(t_2) > 0, \tag{6.2}$$

which is in contradiction with $I_h(t_2)' \leq 0$. The same argument can be made for $R_h(t)$, $S_{SS}(t)$, $E_{SS}(t)$, $I_{SS}(t)$, $S_{SR}(t)$, $E_{SR}(t)$, $I_{SR}(t)$, $S_{RR}(t)$, $E_{RR}(t)$ and $I_{RR}(t)$. Therefore, starting with

model system (2.2) with non-negative initial data in $\mathbb{R}^{12}_{+}$, the solution obtained is non-negative for all $t > 0$. $\square$

6.0.2. Malaria free equilibrium (MFE) state and Basic Reproduction number $\mathcal{R}_0$

The MFE is obtained by setting the left hand side of model system (2.2) to zero and considering the infectious classes in both human and mosquito populations to be zero. At MFE, let $S_h(t) = S_h^*$, $I_h(t) = I_h^*$, $R_h(t) = R_h(t)^*$, $S_{SS}(t) = S_{SS}^*$, $E_{SS}(t) = E_{SS}^*$, $I_{SS}(t) = I_{SS}^*$, $S_{SR}(t) = S_{SR}^*$, $E_{SR}(t) = E_{SR}^*$, $I_{SR}(t) = I_{SR}^*$, $S_{RR}(t) = S_{RR}^*$, $E_{RR}(t) = E_{RR}^*$ and $I_{RR}(t) = I_{RR}^*$. Thus, the malaria free equilibrium during a bed net campaign ($\mu_{vr} \neq \mu_{vs}$), MFE state $E_0 = (S_h^*, I_h^*, R_h^*, S_{SS}^*, E_{SS}^*, I_{SS}^*, S_{SR}^*, E_{SR}^*, I_{SR}^*, S_{RR}^*, E_{RR}^*, I_{RR}^*)$ for model system (2.2), is given by $E_0 = \left(\dfrac{\Lambda_h}{\mu_h}, 0, 0, \dfrac{p^2\Lambda_v N_v^*(K - N_v^*)}{K(\mu_{vs} + m\sigma_s)}, 0, 0, \dfrac{2pq\Lambda_v N_v^*(K - N_v^*)}{K(\mu_{vr} + m\sigma_r)}, 0, 0, \dfrac{q^2\Lambda_v N_v^*(K - N_v^*)}{K(\mu_{vr} + m\sigma_s)}, 0, 0\right)$. Using the next generation matrix method [38], the basic reproduction number for system (2.2) is obtained by letting the rate of appearance of new malaria infection be given by $\mathcal{F}$ and the rate of transfer of infections through different classes $\mathcal{V}$ as follows;

$$F = \begin{bmatrix} \frac{\tau_h(I_{SS}+I_{SR}+I_{RR})S_h}{N_h}\left(m\beta_{bloc}\rho_{in} + (1-m)\rho_{out}\right) \\ \frac{\tau_v I_h S_{SS}}{N_h}\left(m\beta_{bloc}\rho_{in} + (1-m)\rho_{out}\right) \\ 0 \\ \frac{\tau_v I_h S_{SR}}{N_h}\left(m\beta_{bloc}\rho_{in} + (1-m)\rho_{out}\right) \\ 0 \\ \frac{\tau_v I_h S_{RR}}{N_h}\left(m\beta_{bloc}\rho_{in} + (1-m)\rho_{out}\right) \\ 0 \end{bmatrix}, \mathcal{V} = \begin{bmatrix} \varphi I_h + (\mu_h + \gamma) I_h \\ \theta E_{SS} + (m\sigma_s + \mu_{vs}) E_{SS} \\ (m\sigma_s + \mu_{vr}) I_{SS} - \theta E_{SS} \\ \theta E_{SR} + (m\sigma_r + \mu_{vr}) E_{SR} \\ (m\sigma_r + \mu_{vr}) I_{SR} - \theta E_{SR} \\ \theta E_{RR} + (m\sigma_r + \mu_{vr}) E_{RR} \\ (m\sigma_r + \mu_{vr}) I_{RR} - \theta E_{RR} \end{bmatrix} \tag{6.3}$$

Thus, the Jacobian matrices F and V is evaluated at MFE, where $I_h^* = 0$, $R_h^* = 0$, $E_{SS}^* = 0$, $I_{SS}^* = 0$, $E_{SR}^* = 0$, $I_{SR}^* = 0$, $E_{RR}^* = 0$ and $I_{RR}^* = 0$ to obtain;

$$F = \begin{bmatrix} 0 & 0 & \frac{\tau_h S_h^* \omega_{net}}{N_h^*} & 0 & \frac{\tau_h S_h^* \omega_{net}}{N_h^*} & 0 & \frac{\tau_h S_h^* \omega_{net}}{N_h} \\ \frac{\tau_v S_{SS}^* \omega_{net}}{N_h^*} & 0 & 0 & 0 & 0 & 0 & 0 \\ 0 & 0 & 0 & 0 & 0 & 0 & 0 \\ \frac{\tau_v S_{SR}^* \omega_{net}}{N_h^*} & 0 & 0 & 0 & 0 & 0 & 0 \\ 0 & 0 & 0 & 0 & 0 & 0 & 0 \\ \frac{\tau_v S_{RR}^* \omega_{net}}{N_h^*} & 0 & 0 & 0 & 0 & 0 & 0 \\ 0 & 0 & 0 & 0 & 0 & 0 & 0 \end{bmatrix}, V = \begin{bmatrix} \varphi + \mu_h + \gamma & 0 & 0 & 0 & 0 & 0 & 0 \\ 0 & \theta + m\sigma_s + \mu_{vs} & 0 & 0 & 0 & 0 & 0 \\ 0 & -\theta & m\sigma_s + \mu_{vs} & 0 & 0 & 0 & 0 \\ 0 & 0 & 0 & \theta + m\sigma_r + \mu_{vr} & 0 & 0 & 0 \\ 0 & 0 & 0 & -\theta & m\sigma_r + \mu_{vr} & 0 & 0 \\ 0 & 0 & 0 & 0 & 0 & \theta + m\sigma_r + \mu_v & 0 \\ 0 & 0 & 0 & 0 & 0 & -\theta & m\sigma_r + \mu_v \end{bmatrix}$$

where $\omega_{net} = m\beta_{bloc}\rho_{in} + (1-m)\rho_{out}$. The basic reproduction number $\mathcal{R}_0$ is the spectral radius of the next generation matrix FV^{-1} given by;

$$\mathcal{R}_0^L = \sqrt{\frac{\tau_h\Lambda_h\omega_{net}}{\mu_h(\varphi+\gamma_h+\mu_h)}} \times \sqrt{\frac{\theta\tau_v p^2\omega_{net}N_v^*(K-N_v^*)}{K(m\sigma_s+\mu_{vs})^2(\theta+m\sigma_s+\mu_{vs})} + \frac{q(2p+q)\tau_v\Lambda_v\omega_{net}N_v^*(K-N_v^*)}{K(m\sigma_r+\mu_{vr})^2(\theta+m\sigma_r+\mu_{vr})}}$$

and N_v^* is the total mosquito population at MFE.

Sensitivity analysis figure

Click here to access/download;Figure;sensitivity_analysis.png

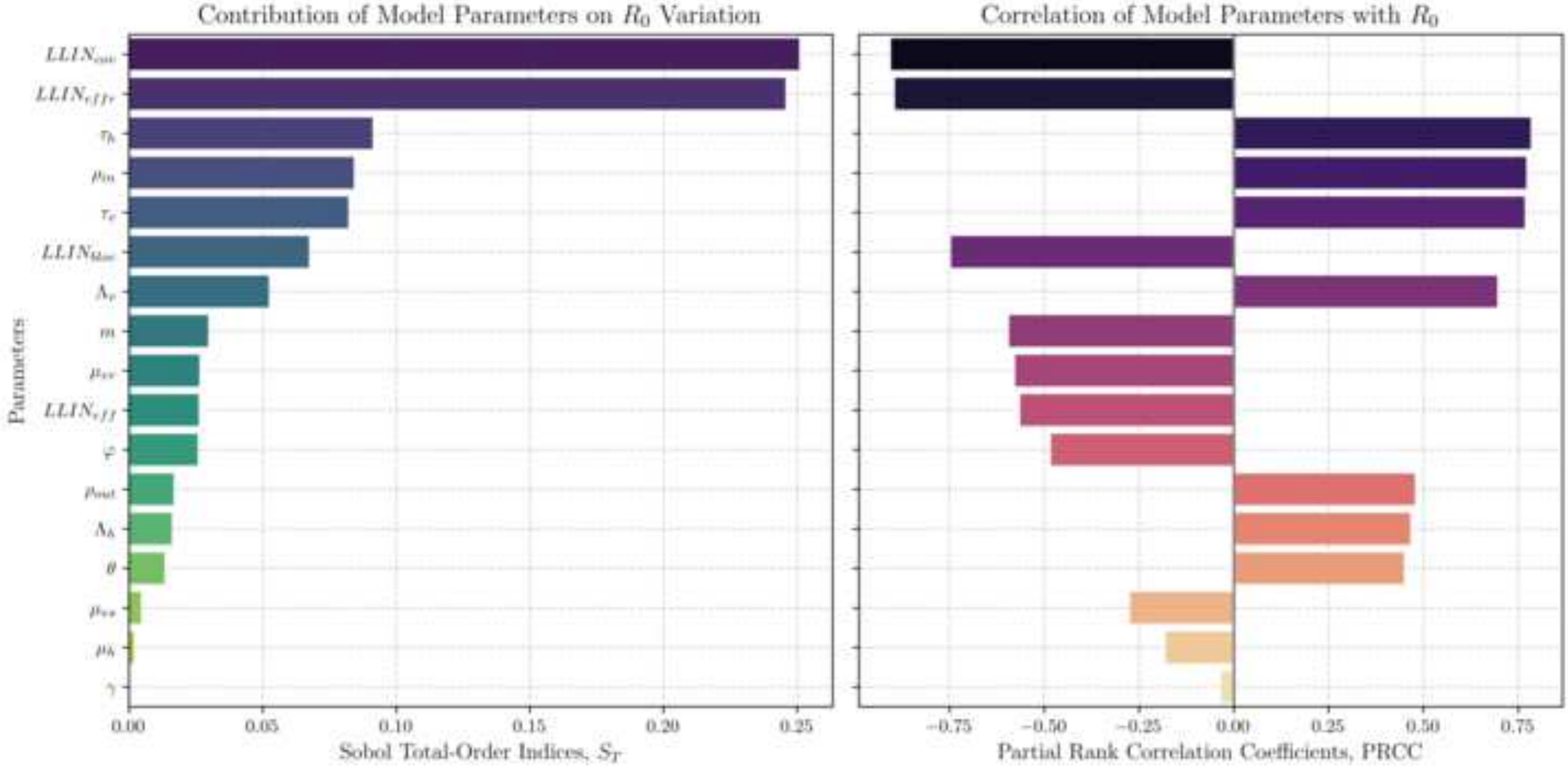

Summarized impact of LLIN on malaria dynamics

Click here to access/download;Figure;summarized_net_impact_human_malaria_dynamics_4.png

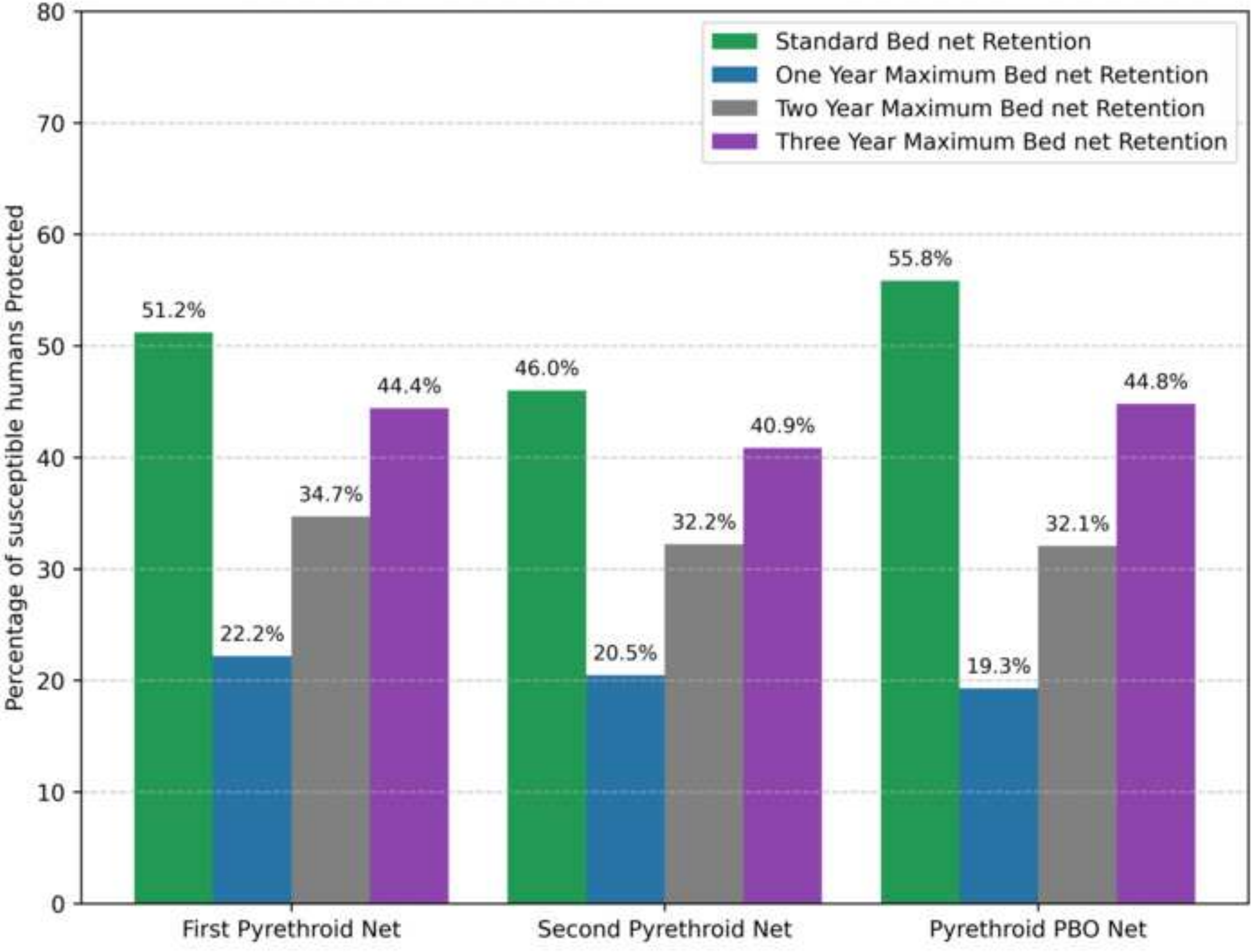

Click here to access/download;Figure;three_campaign_genotype_plot_4.png

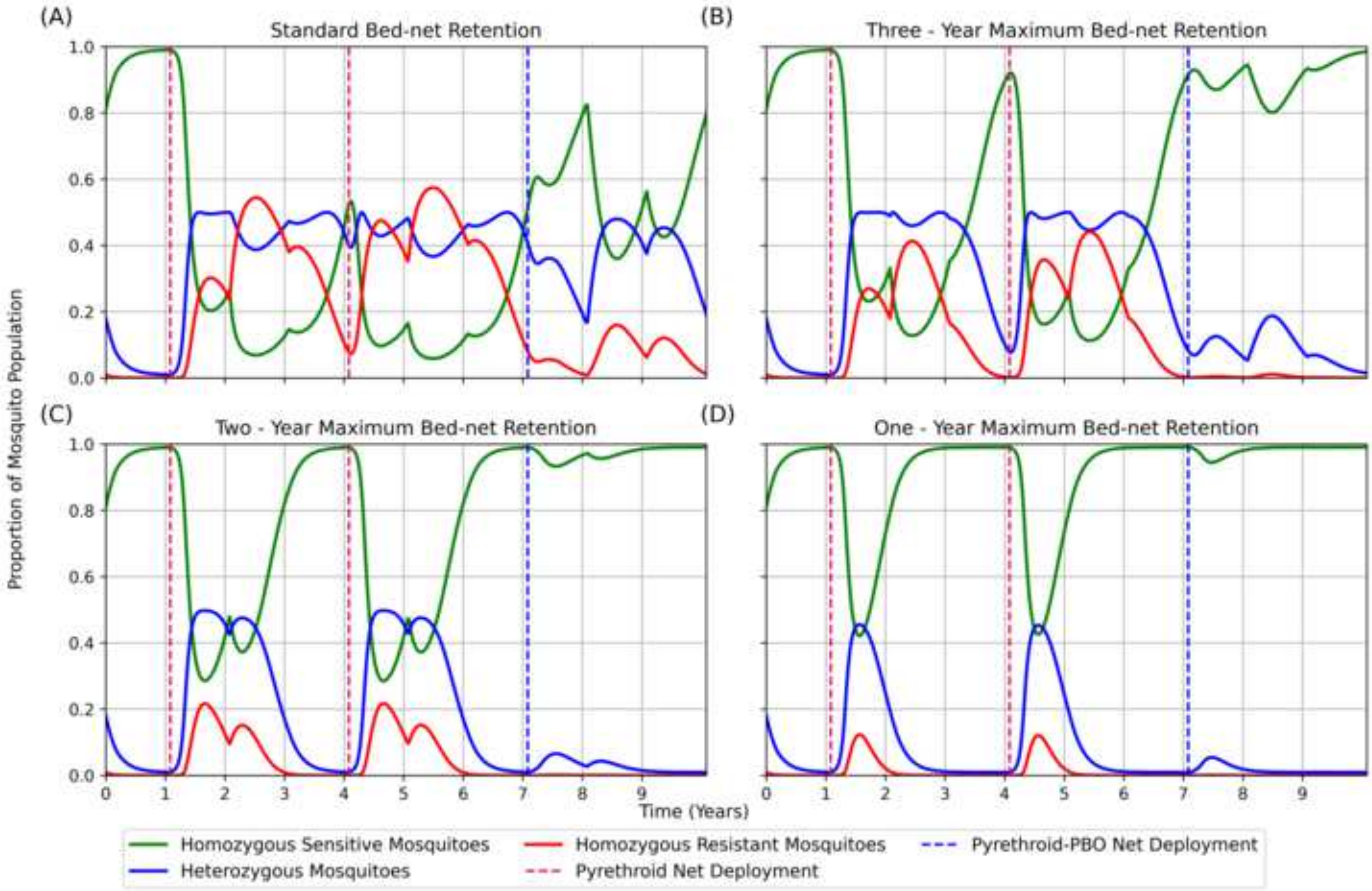

Click here to access/download;Figure;three_campaign_human_malaria_dynamics_4.png

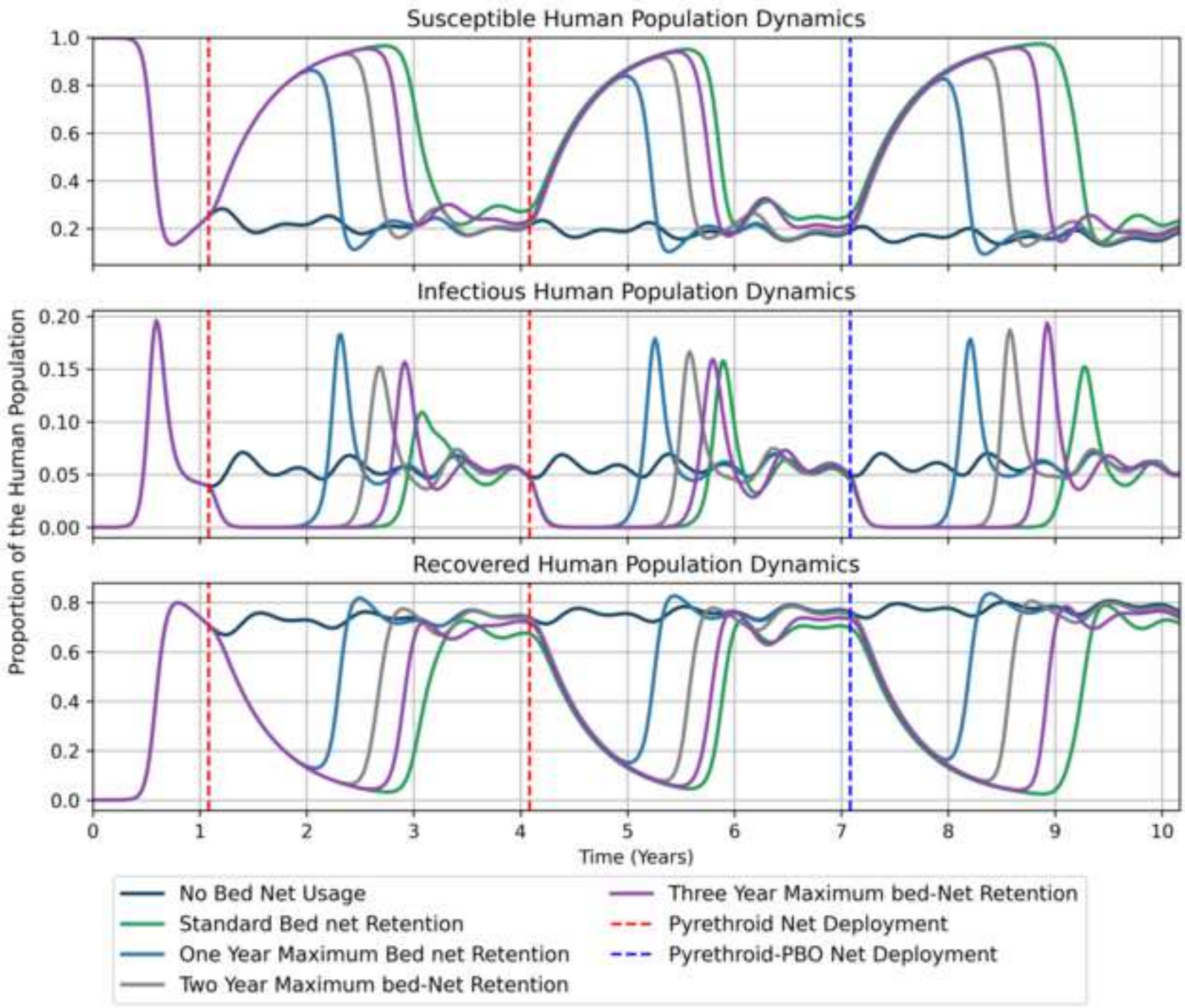

Click here to access/download;Figure;three_campaign_resist_mosquito_epide

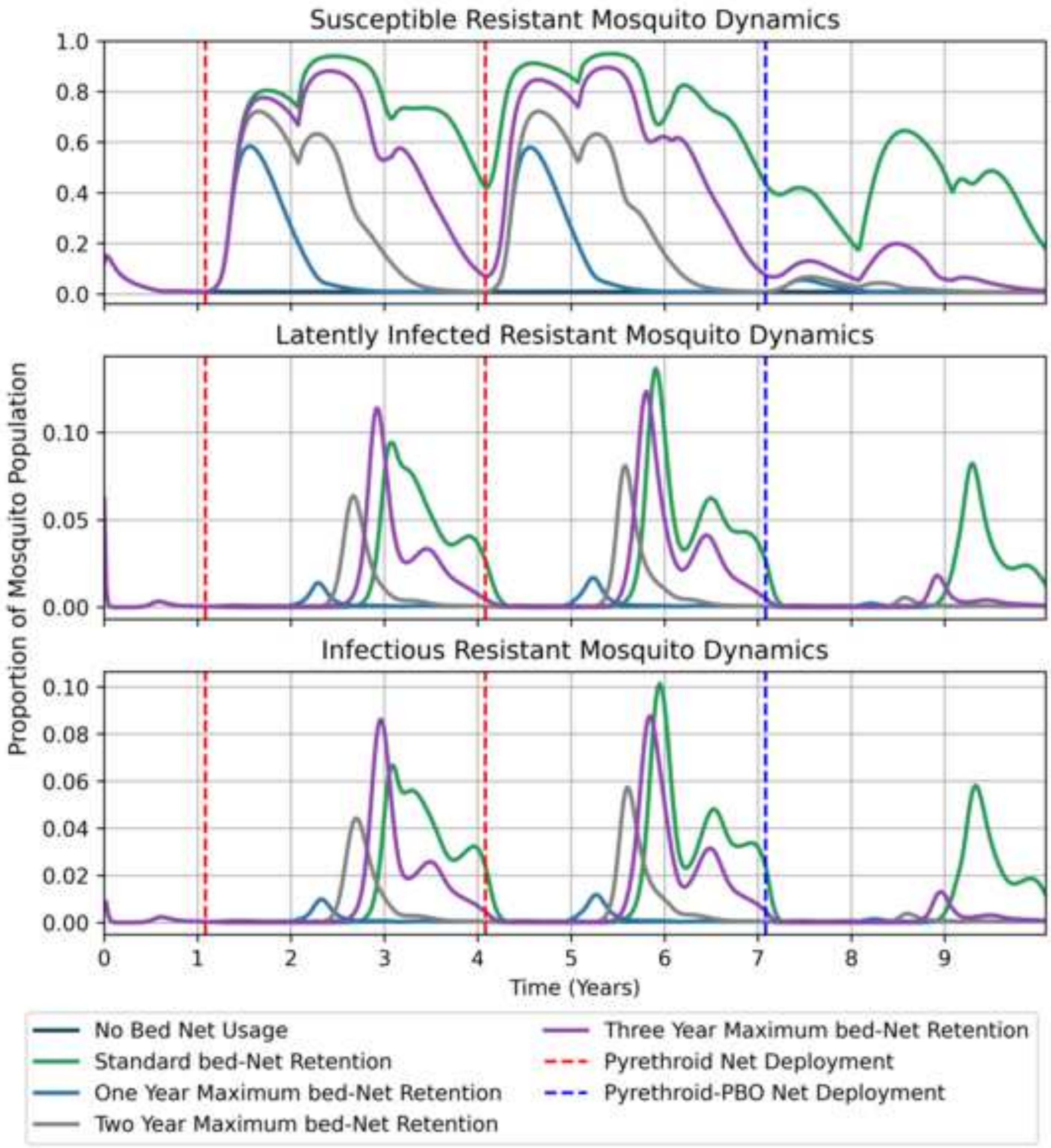

Impact of LLINs on Sensitive Vector

Click here to access/download;Figure;three_campaign_sensitive_mosquito_epidemiology_plot_4.p

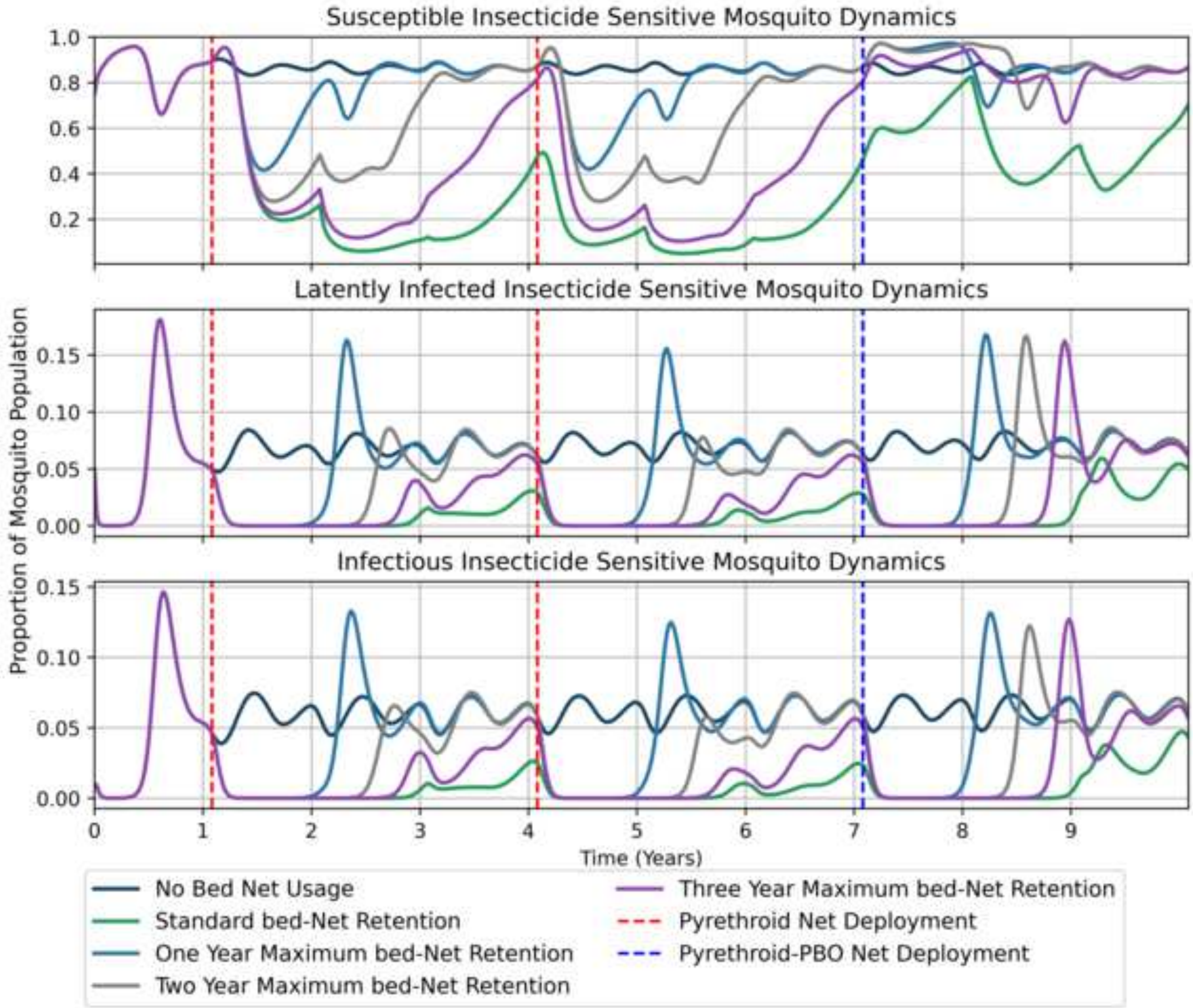

Impact of LLINs on total mosquito Population

Click here to access/download;Figure;three_campaign_total_mosquito_dynamics_plot_4.png

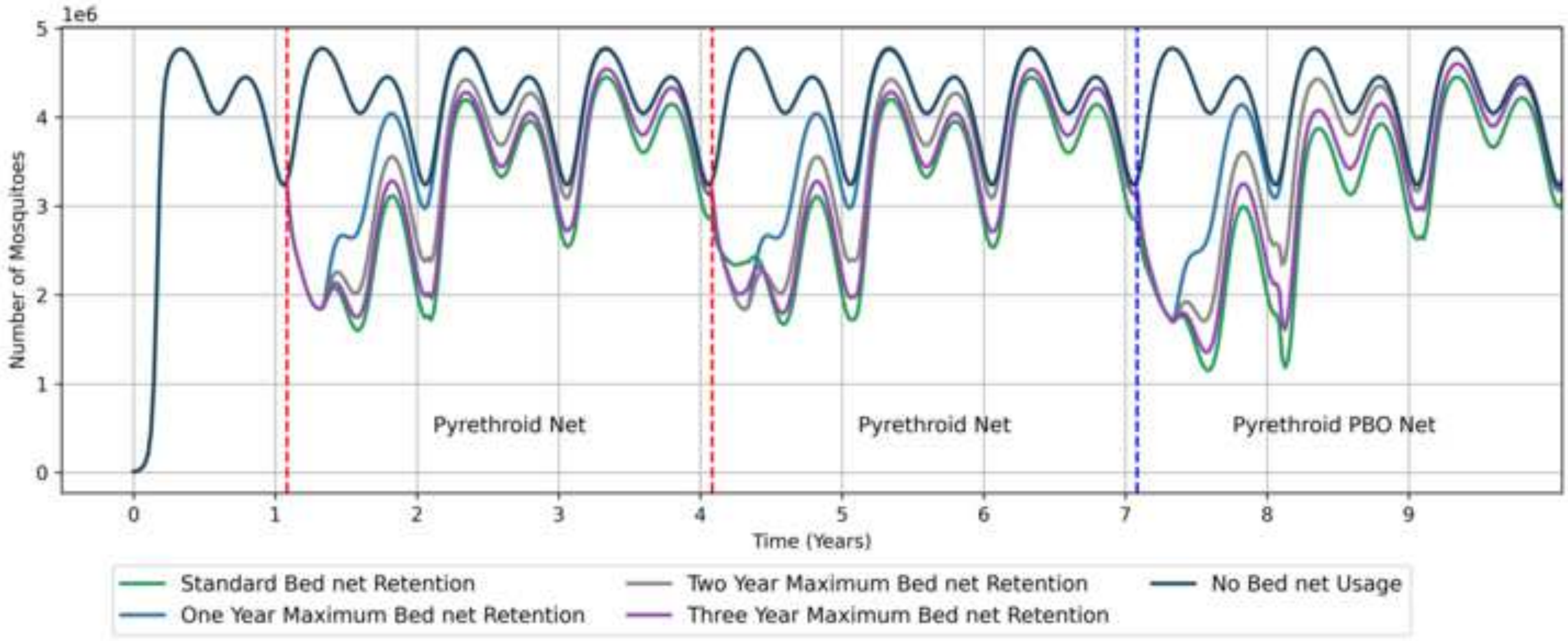

Effect of fitness cost on Resistance Evolution

Click here to access/download;Figure;total_mosquito_resistance_evolution_4.

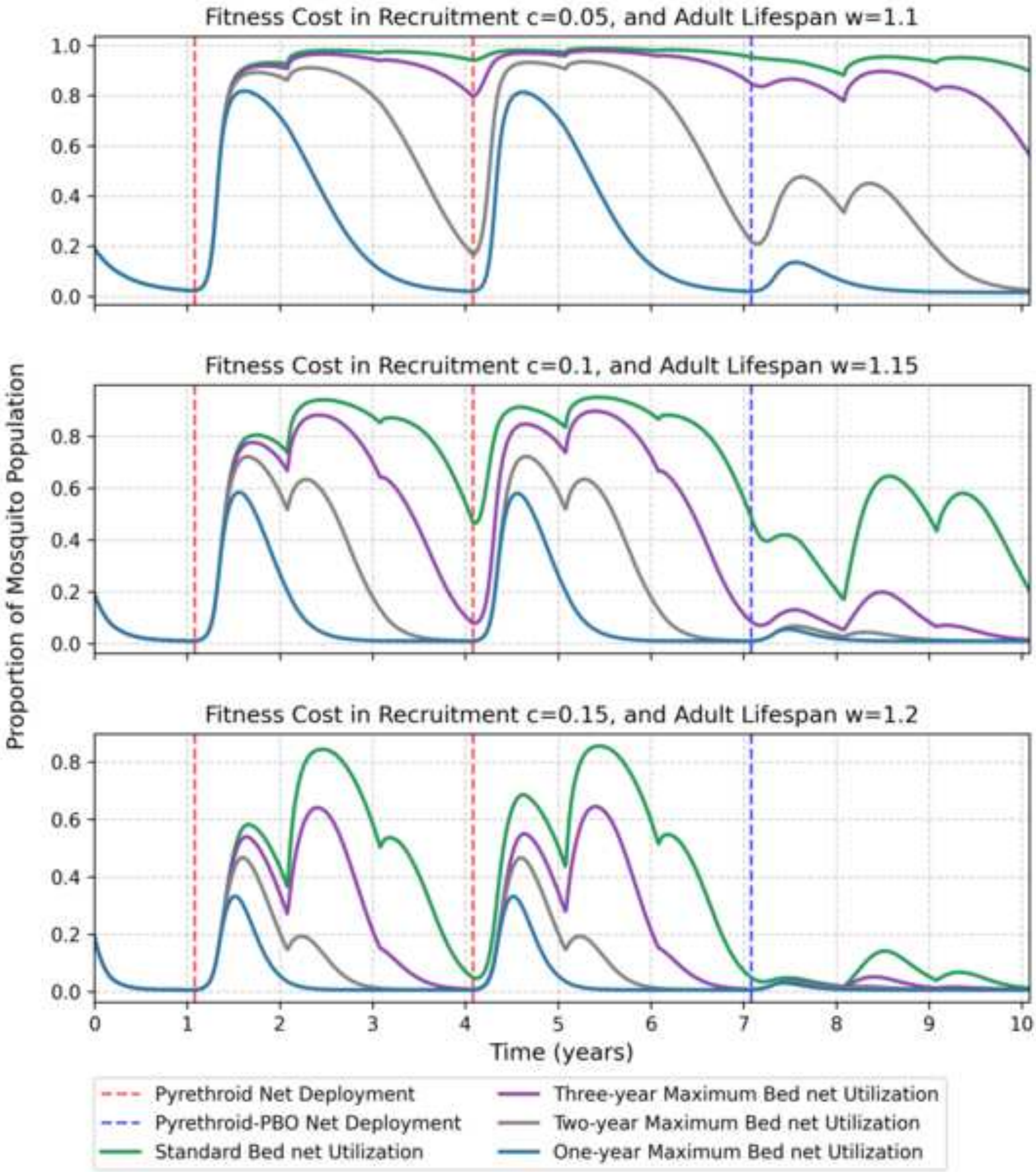

Supplement: Attachment — Submitted filename: PONE-D-25-51344_reviewer.pdf [file pone.0353301.s003.pdf]
