# Supplementary material for: Modelling the impact of mosquito bed net utilization on malaria transmission and evolution of pyrethroid resistance
Source: PLoS One. 2026 Jul 15;21(7):e0353301. doi: 10.1371/journal.pone.0353301 (PMC13372178; doi:10.1371/journal.pone.0353301)
Supplement: Attachment — Submitted filename: Response to Reviewers.pdf [file pone.0353301.s004.pdf]

January 02, 2026

Makerere University,

P.O Box 7062,

Kampala, Uganda.

Phone: +256705461622

Email: isseguya@gmail.com

Through the Academic Editor, PLOS ONE,

1875 Mission Street, Suite 103 #188,

San Francisco, CA 94103,

United States.

Dear Editor and Reviewers,

RE: Response to the Academic Editor and Reviewers Comments

On behalf, of the authors for manuscript titled "Modelling the impact of mosquito bed net utilization on malaria transmission and evolution of pyrethroid resistance", we thank we thank the editor and reviewers for their careful comments about our manuscript and constructive feed back provided. This manuscript has been revised in response to the comments to improve its quality and a detailed account of these revisions have been reported as follows.

(a) Academic editor

The manuscript has been revised to meet the PLOS ONE structure using the PLOS LaTeX template. The funding information has been withdrawn from the manuscript body (particularly, acknowledgment section) and included in the funding statement as guided.

Funding statement: This study was supported by the African Consortium in Modelling for Effective Vector Control (ACoMVeC) project INV-047049. However, the sponsors

did not have any role in the study design, choice of parametrization, analysis, or manuscript preparation and publication.

(b) Reviewer One:

(i) Comment: Could the authors kindly define what they mean with 'Malaria Dynamics' for clarity.

Action: The authors of this manuscript appreciate calling out this, and the manuscript title has been modified to: "Modelling the impact of mosquito bed net utilization on malaria transmission and evolution of pyrethroid resistance". See the modified manuscript title on page one.

(ii) Comment: From the abstract: "This study formulates a genotype-specific compartmental model accounting for malaria transmission between the human and vector populations." From the epidemiological model, for a successful malaria transmission, three things come into play: Host, Agent and environment. Were this accounted for by the model?

Action: The environment was not modeled as an explicit compartment, but its effects were captured through terms like mosquito recruitment (particularly logistic growth and carrying capacity). Additionally, the aquatic mosquito life stage was not explicitly considered for this model because the model did not account for aquatic stage vector control measures like larval source management and larviciding.

(iii) Comment: "It incorporates the blocking and insecticidal killing effects of LLINs, and pyrethroid-induced fitness costs, to evaluate the impact of LLIN utilization on malaria and resistance dynamics." How was this done? Blocking effect is as a result of fabric integrity, killing effect is all about the chemical/bioefficacy effect. Why did the authors look at the pyrethroid fitness costs, we are aware that pyrethroids are the cheapest forms of

insecticides used for Longlasting insecticidal nets (LLINs) protection. Do we have any comparative parameter for this aspect in this study?

Action: The blocking effect was applied in the malaria transmission term. This was represented by β_bloc = 1 - (f X LLIN_cov X LLIN_bloc), where f is the proportion of mosquitoes that blood feed indoors, LLIN_cov is the effective bed net coverage and LLIN_bloc is the effectiveness of bed net blocking. For numerical simulations, both LLIN_cov and LLIN_bloc were considered to decay exponentially through the duration of the campaign period. In this case, the overall effectiveness of LLIN blocking covers the physical/ fabric integrity.

Insecticide killing effect was applied as an added mosquito mortality (in addition to natural mortality rate). This killing effect was set to depend on the LLIN effective coverage and considered to decay exponentially over the campaign period. Refer to the model system on page page 5.

Pyrethroid fitness costs are a key determinant of evolutionary and epidemiological consequences of insecticide resistance. The reduction in mosquito fitness through reduced fecundity and adult life expectancy affects the total mosquito population and consequently evolution of pyrethroid resistance and overall effectiveness of bed net intervention. This motivated the inclusion of fitness costs in the model structure.

(iv) Comment: "Reproductive numbers, with bed net usage R^L_0 and without bed net usage R_0, were calculated and sensitivity analysis conducted." What is Reproductive numbers in this case?

Action: Thank you for this, the sentence structure was revised foe better clarity, see abstract section on page 1.

(v) Comment: According to WHO, bed net usage is an individual having slept under a net

the previous night prior the survey. Was this considered by the model?

Action: Yes. While the model does not track individual-level nightly behavior, bed net utilization is represented at population level as effective coverage. Effective coverage, in this regard, refers to the proportion of humans that is covered by LLINs and uses them correctly on a given night. This is consistent with WHO interpretation, but implemented in as a time varying parameter to cater for decay in LLIN effectiveness. See page 8, lines 206 - 211.

(vi) Comment: And how was sensitive analysis done? Ideally, at this point what I mean is that the authors must make it very clear, in a manner that readers of same and different backgrounds can understand without specific scientific training. Now that the study looks/is determined towards public health impact, can policy makers understand the take-home messages if they were to be guided by WHO guidelines?

Action: Sensitivity analysis was done by varying all model parameters responsible for malaria transmission across a wide range of possible values at the same time. We then used the Sobol and Partial rank correlation coefficient (PRCC) to determine which parameters consistently had the strongest influence on malaria transmission.

The write up for sensitivity analysis section has been toned down to accommodate a reader with a background lucking such techniques, and the take home message was made clearer. See page 10, lines 229 - 234.

(vii) Comment: The title of the manuscript is “Modelling the Impact of Mosquito Bed Net Utilization on Malaria Dynamics and Evolution of Pyrethroid Resistance”, yet we see the authors in the results section of abstract talk about LLIN coverage. Coverage is different from utilization. What do the authors mean with “highest” in “highest effect in reducing malaria transmission”, what was the measure or level?

Action: The "LLIN coverage" was originally used to mean effective LLIN coverage or utilization. To improve clarity, the sentence structure has been modified and we decided to stick on LLIN utilization other than "LLIN coverage". See abstract section on page 1, section 4 page 8 line 178, and line 253 page 10.

Also, "highest effect in reducing malaria transmission" was used to mean parameters that yielded the largest sensitivity indices for both Sobol and Partial rank correlation coefficient (PRCC). To improve clarity, this sentence structure has been modified to "The utilization of LLINs and their effectiveness against pyrethroid-resistant vectors demonstrated the strongest effect in reducing malaria transmission in both Sobol and partial rank correlation coefficient PRCC analyses making them the most influential parameters in reducing malaria infections". See abstract section on page 1.

(viii) Comment: Please the authors need to take note of this: Despite of an initial LLIN coverage of 81% (Where?/When? How was this obtained?), without consistent utilization (What is the meaning of this? What was the measure? How do we define consistent usage?), protection (measure of protection has not been provided please or talked about in the abstract) against malaria infection from pyrethroid only and pyrethroid piperonyl butoxide (PBO) nets, was not sustained over time (What was the timeline?).

Action: Clarity for this section of the abstract was improved by introducing the context before the simulation findings of the study. The 81% initial bed net coverage was motivated by the universal bed net campaign coverage for the LLINEUP Project in Uganda between 2017 and 2018 from which different research studies have been conducted, for example by Mechan *et al.* (2022). In this study, consistent utilization/ usage was meant to refer to regular and proper use of bed nets every night by people who own them throughout the campaign period. This has been streamlined in the manuscript. See page 8

lines 180 - 191, and Table 1 on page 9 .

The measure protection of bed nets against malaria infection was in terms of the proportion of susceptible humans prevented by bed nets from becoming infected with malaria. This was highlighted in section 4, subsection 4.2 line 253 - 255 of the manuscript. “Over time” was used to mean the three year campaign period. This has been rectified in the manuscript (see abstract section and page 8 line 190).

(ix) Comment: PBO (Start a sentence always in full not abbreviations-This needs also to be considered throughout the manuscript text) nets averted more infections and suppressed malaria vectors by 72% (does this account for the parasite and the vector?) under standard utilization (What is the meaning of standard utilization), although this effectiveness declined with shorter retention periods.

Action: Start of sentences with abbreviations, like “PBO” was rectified both in the abstract and entire body of the manuscript.

No, the 72% reduction only accounts for a reduction in the population of malaria vectors.

According to the study, standard bed net utilization refers to a utilization scenario, where LLIN deployment increases effective coverage to 81% at the start of each campaign, after which coverage declines exponentially due to bed net attrition and reduced usage. The rate of decay is such that effective coverage would reach 1% after four years if nets were not replaced. However, effective coverage doesn’t reach the minimum level of 1% since new bed nets are deployed every after three years. See section 4, page 8 lines 182 - 186.

(ix) Comment: Also, the study showed that resistance evolves faster with repeated

pyrethroid-only campaigns, but PBO nets negate this trend especially when such resistance carries a higher fitness cost. While shorter LLIN retention slows the evolution of pyrethroid resistance, it is not a viable option for malaria. Do the authors imply that LLINs may not offer substantial protection? Was there a control to make this conclusion such as the use of indoor residual spraying (IRS)?

Action: The authors do not imply that LLINs provide limited protection. On contrary, the study demonstrates that higher LLIN utilization enhances malaria control and vector suppression. While lower LLIN utilization scenarios had slower evolution of resistance, these are not epidemiologically viable for malaria control. This has been streamlined in both the abstract and discussion section.

The study focuses on only LLIN interventions and does not include IRS. Therefore, the conclusion and abstract have been cleaned to align it with the findings of the study. See page 19 lines 488 and 489, and abstract section on page 2.

(c) Reviewer Two:

Response: All comments by the second reviewer were carefully studied and none of them was found to warrant an explanation or modifications into the drafting of the study.

(d) Reviewer Three:

(i) Comment: Abstract: Line 10: remove “of” in “Despite of an initial LLIN…”.

Action: This has been addressed in the new manuscript edition, see abstract section.

(ii) Comment: Line 7: the quote of 2793 deaths in 2023 related to malaria seems low. I believe the estimate was over 16,000, but official records were 2793. Please incorporate additional information in the deaths related to malaria. Line 9:

“20% of hospital deaths… “ are due to malaria?! 2793 deaths seems low for a population of 50 million.

Action: This has been rectified in reference to the World Malaria reports of 2025 and 2024. See lines 4 - 9 of section 1, pages 1 and 2.

(ii) Comment: Line 17: please describe a value associated with “highly effective”.

Action: This has been revised to quantify the effectiveness of IRS by explicitly reporting effect from the cited meta-analysis. Specifically, we stated that IRS was associated with a 65% reduction in malaria infection risk overall, and a 73% reduction when coverage was at least 80%, as reported by Zhou et al. (2022). See page 2 lines 18 - 21.

(iii) Comment: Line 20: Can you be more specific with the amount of reduction in malaria morbidity?

Action: This reduction in malaria morbidity as a result of IRS has been quantified in the manuscript. See lines 21 - 24, page 2.

(iv) Comment: Line 79: change Mosquito in “The Mosquito population” to lowercase. Line 82: change Malaria Transmission in “The Malaria Transmission” to lowercase.

Action: This has been rectified throughout the entire manuscript. E.g see page page 10 line 251.

(v) Comment: Line 120: you should describe the study in the methods section, not in the results of the study section.

Action: The mathematical model formulated under section 2 (page 3, line 62) is a representation of malaria dynamics between the human and vector population in presence of insecticide resistance, and in this formulation all parameters are fixed (do not change over time). This forms foundation of the study and mathematical properties of this model structure were analyzed.

The introductory descriptions at the start of the numerical results section (page 8) ;

(a) Present simulation scenarios considered in the study, specifying the LLIN utilization periods modeled under scenario-based rates of exponential decay in effective coverage.

(b) Introduce LLIN effectiveness parameters (e.g mosquito blocking effect, insecticide killing effect and effective LLIN coverage) as time dependent, and how they exponentially decay over time according to each LLIN utilization setting.

(c) It also modifies the mosquito recruitment term to follow a seasonal pattern on assumption that more mosquito eggs will become winged mosquitoes during the rainy season than dry season.

Therefore, such considerations and modifications where only applied in the numerical simulation and it would be misleading describing them at the start of section 2 (methods section).

(vi) Comment: Line 123: Please be more descriptive, does the efficacy of the bed nets diminish from 100% to 1% over a year, 2 years, and 3 years, but the nets are replaced yearly?

Action: This has been rectified and it is now explicitly stated that effective coverage begins at 81% upon bed net deployment, and decays exponentially up to 1%. The bed nets are replaced at the end of each campaign period (each campaign is expected to last three years). See section 4, lines 181 - 191.

(vii) Comment: Line 129-130: Please describe in section 4 and/or the methods section that the model parameters have references for their selection listed in Table 1.

Action: This has been addressed in section 4, lines 205 - 206.

(viii) Comment: Line 233: Not sure what "Susceptible pyrethroid-resistant" means… does that mean they are susceptible to malaria?

Action: This class refers to vectors which are susceptible to malaria infection (are prone to getting infected with malaria) and are resistant to pyrethroid insecticides.

(ix) Comment: Line 281-284: the phrasing here is inaccurate. PBO cannot restore pyrethroid sensitivity in mosquitoes. It acts as a synergist to suppress the detoxification enzymes rendering the resistant mosquitoes susceptible to pyrethroids they would normally be resistant to. Unfortunately, metabolic resistance is not the only form of insecticide resistance in mosquitoes and these model results may be misleading.

Action: This has been rectified in the manuscript document: see pages 15 and 16, lines 364 - 370.

(x) Comment: Line 303 and 320: What is the standard utilization scenario?

Action: This refers to bed net utilization scenario, where LLIN deployment increases effective coverage to 81% at the start of each campaign, after which coverage declines exponentially due to bed net attrition and reduced usage. The rate of decay is such that effective coverage would reach 1% after four years if nets were not replaced. However, effective coverage doesn't reach the minimum level of 1% since new bed nets are deployed every after three years. See section 4, page 8 lines 185 - 189.

(xi) Comment: Line 328: What are the fitness costs found in nature?

Action: Fitness cost refers to the reduction in one or more life history traits, like survival, fecundity, rate of development or mating success, experienced by insecticide resistant mosquitoes relative to insecticide sensitive vectors in

absence of insecticide pressure.

In this study insecticide resistant mosquitoes (particularly heterozygous and homozygous resistant vectors) were considered to suffer fitness costs in terms of reduced fecundity and reduced adult longevity.

(xii) Comment: Line 366-367: You only assessed pyrethrin and PBO for the third year, how can you compare pbo with the other synergists/AI's in this list

Action: No, PBO was applied in the third campaign not year. In this line, PBO was not compared with other synergists/AI's in this list. The statement on page 18, lines 456 - 458 is discussing a result from figure 9 (page 17) which indicates that pyrethroid-PBO nets performed better in cases where mosquitoes incurred higher fitness cost (across all utilization scenarios) in terms of slowing the evolution of pyrethroid resistance.

(xii) Comment: Please be sure to describe your tables and figures in a way that if they were separated from the main body of the manuscript, they could stand on their own. As they are currently written they cannot. For example Fig. 3 caption: "Effect of reducing LLIN retention periods of malaria dynamics across three bed net campaigns." This makes no reference to the fact that these are simulated values generated by a model, and not actual values from a real-world experiment. Be sure to consider using colors and patterns in your figures that are better for color blind readers.

Action: Tables and figure captions have been modified. They can now stand on their own if separated from the main body of the manuscript. E.g see pages 9, 11, 12,13, 14, 15 and 16.

Sincerely,

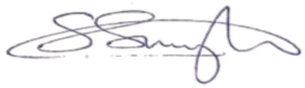

Ivan Sseguya
