# Supplementary material for: Modelling the impact of mosquito bed net utilization on malaria transmission and evolution of pyrethroid resistance
Source: PLoS One. 2026 Jul 15;21(7):e0353301. doi: 10.1371/journal.pone.0353301 (PMC13372178; doi:10.1371/journal.pone.0353301)
Supplement: Attachment — Submitted filename: Response_to_Reviewers_auresp_2.pdf [file pone.0353301.s005.pdf]

February 05, 2026

Makerere University,

P.O Box 7062,

Kampala, Uganda.

Phone: +256705461622

Email: isseguya@gmail.com

Through the Academic Editor, PLOS ONE,

1875 Mission Street, Suite 103 #188,

San Francisco, CA 94103,

United States.

Dear Editor and Reviewers,

RE: Response to the Academic Editor and Reviewers Comments

(a) Reviewer 1:

No comment was registered to warrant feed back.

(b) Reviewer 4:

(i) Comment: There is no uniformity for WHO. for example Ref no 43. Organization WH. But in all other references it is WHO.

Action: This was corrected, with World health Organization cited in the proper form.

(ii) Comment: In reference no. 71. Neither Author/s nor publisher have been mentioned.

Action: This was addressed to include author, year of publication and publisher.

Sincerely,

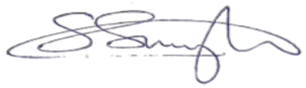

Ivan Sseguya
