# Supplementary material for: Modelling the impact of mosquito bed net utilization on malaria transmission and evolution of pyrethroid resistance
Source: PLoS One. 2026 Jul 15;21(7):e0353301. doi: 10.1371/journal.pone.0353301 (PMC13372178; doi:10.1371/journal.pone.0353301)
Supplement: Attachment — Submitted filename: Response to Reviewers_march26.pdf [file pone.0353301.s006.pdf]

March 29, 2026

Makerere University,

P.O Box 7062,

Kampala, Uganda.

Phone: +256705461622

Email: isseguya@gmail.com

Through the Academic Editor, PLOS ONE,

1875 Mission Street, Suite 103 #188,

San Francisco, CA 94103,

United States.

Dear Editor and Reviewers,

RE: Report on the Required Reviewer - Revisions

On behalf, of the authors for manuscript titled "Modelling the impact of mosquito bed net utilization on malaria transmission and evolution of pyrethroid resistance", we thank we thank reviewers for their careful comments about our manuscript and constructive feed back provided. This manuscript has been revised in response to the comments to improve its quality and a detailed account of these revisions have been reported as follows.

(a) Reviewer Five:

(i) Comment: Line 18: Instead of "systematic review", should state "meta-analysis", because you are reporting the numerical calculations, not the literature review portion.

Action: This line makes reference to numerical results. Therefore, the wording has been modified to "meta-analysis", see page 2 line 18.

(ii) Comment: Line 21: aversion of "68% of 663 million malaria cases registered" does not make sense, because these were reported cases. Please revise your wording

for clarity.

Action: The 663 million refers to the total number of malaria cases averted globally, from which LLINs have a contribution of 68%. This has been modified in the text, see page 2 line 21.

(iii) Comment: Lines 42 and 43: For clarity, "bed net utilization" should read "bed net retention time per campaign".

Action: Reference is made in terms of the median time for bed net retention. Therefore, “retention” is the most appropriate word to use under this context. This has been revised, see page 2 lines 43 and 44.

(iv) Comment: Lines 96-100, All these "assumptions" are limitations which should be discussed in further detail in the discussion

Action: These have been included among the limitations, see page 19 lines 482 to 484.

(v) Comment: Line 101: Modelling recruitment of new mosquitoes is defined on line 86, but not clear here, where logistic term A is discussed.

Action: The recruitment function is given by $\Lambda_v N_v(1 - N_v/K)$, but this is made logistic because of the term $N_v(1 - N_v/K)$. The A to represent the term $N_v(1 - N_v/K)$ was adopted to reduce crowding on the compartment diagram. Thus , o the compartmental diagram, recruitment was represented by $\Lambda_v A$.

(vi) Comment: Line 115: Please explain epidemiological feasible region in words and describe its implication in this research. Also direct readers to the Appendix for equation and further understanding.

Action: Epidemiological feasible region was used to refer to a region where all model variables and parameters remain positive and bounded for all time, and the population remains non-negative and biologically realistic. In the manuscript, this modification was effected on page 5 line 115 and 116, and page 20 line 504. And, the reader has been directed to the Appendix in this paragraph.

(vii) Comment: Line 183: The way you decided upon rate of decay of bed net use was not clear and produced some questionability in my mind of major findings, until I dug deeper into the references you had used. I think more communication

describing these decisions would help manuscript narrative.

Action: Bed net utilization was assumed to decay exponentially over time and at any time t, the level of bed net utilization was expressed as $\mathrm{LLIN}_{cov}(t) = \mathrm{LLIN}_{cov}(0)\exp(-\delta_{cov} t)$, where $\mathrm{LLIN}_{\mathrm{cov}}(0) = 0.81$ is the initial bed net utilization and $\mathrm{delta}_{\mathrm{cov}}$ is the rate of decay in effective bed net coverage, such that: $\delta_{cov} = \frac{\ln(\mathrm{LLIN}_{cov}(0)/\mathrm{LLIN}_{\min})}{T}$, For more details see page 11 lines 183 to 200.

(viii) Comment: Line 186: Remove the phrase "On the hand," completely. It is not grammatically correct and does not lend information or context.

Action: The phrase "On the hand," has been removed from the entire manuscript. For example see page 11 lines 192.

(ix) Comment: Lines 219-226: The utility of this paragraph is misleading, since the model assumes no fitness costs are incurred by malaria vectors (line 126).

Action: Fitness costs occur in presence of selection pressure from LLIN campaigns, thus without bed net deployment, mosquitoes are expected to be free from fitness costs. This phrase "assuming no fitness costs are incurred by malaria vectors" has been removed from line 137 (page 6) and considerations for the analytical section and numerical simulation part of the model described in the first paragraph of the "model description and formulation section", see line 62 on page 3.

(x) Comment: Line 232: Partial Rank Correlation techniques were not discussed in the manuscript

Action: The PRCC values were obtained by rank-transforming inputs and outputs, removing effects of other parameters via regression, and correlating the resulting residuals using Spearman's method on the Sobol-generated sample set, see page 9 lines 248 to 251.

(xi) Comment: Lines 241-242: These results highlight the importance of LLINcov and LLINeffr, and the narrative surrounding modelling and mathematical

decisions surrounding these variables should be clear and sound throughout the manuscript.

Action: The importance of LLINcov and LLINeffr was highlighted because these act directly on the human–vector contact and mosquito survival components transmission process, which represent primary pathways driving malaria spread in the model system. For details see page 9 lines 259 to 264.

(xii) Comment: Line 243: Word "this" should be removed Overall, the way model results read should be clear that this was a simulation and these are results OF THIS STUDY, not a global truth. Using present tense language makes it appear as a generalizable truth instead of simply reporting your results. This is consistent throughout the manuscript and should be revised for clarity.

Action: The word *'this'* has been removed from the line. Additionally, the results section has been revised to make clear that the reported findings derive from model simulations specific to this study, rather than representing generalizable truths, see page 9 and 10, lines 256 to 279.

(xiii) Comment: Lines 244-246: The way that "rate of parasite development in vectors" is repeated reads confusingly. Additionally, clarify that increase in theta had the smallest effect OF THE INCLUDED PARAMETERS on malaria transmission.

Action: This was renamed to "rate at which latently infected vectors become infectious (theta)" and the sentence was modified to be consistent with the parameter definition in the table of model parameters, see page 10 lines 268 to 270.

(xiv) Comment: Lines 263-264 and Fig 3: Range of suppression of malaria cases from 0 to close to 100% appears strikingly unrealistic

Action: The malaria cases are suppressed from 5% to less than 1%, but as the LLIN effectiveness wane off over the campaign period, these "recover" (towards 5%), see page 11 lines 290 and 291.

(xv) Comment: Lines 266-267: As in the rest of the manuscript, results should be

presented in past tense, not present tense

Action: The past tense was adopted in all reporting of model results, see page 14 lines 326, 339, 345, 349, page 17 line 397, page 18 line 421, page 19 line 456, and page 11 lines 287, 293 to 295. The present tense was maintained in the conclusion section.

(xvi) Comment: Lines 282-284: Please re-evaluate and rephrase this section for clarity, without speculation. No advantage was registered from the use of pyrethroid PBO nets during the third campaign? I see that each bednet campaign was expected to last a period of three years, and pyrethroid-PBO nets were introduced in the third campaign, at year 7 of the simulation. Line 277 states that PBO LLINs prevent more malaria infections due to ability to kill sensitive and resistant vectors. Line 314 reports that PBO nets reduced mosquitoes to near zero for the most of the campaign period, Please re-analyze and clarify.

Action: “No advantage” was used to mean that pyerethroid-PBO LLINs had no advantage over pyrethroid-only nets under the Two and One year maximum utilization scenarios. This has been re-written to:“Figure 4 also demonstrated that the advantage of pyrethroid-PBO LLINs over pyrethroid-only nets, in terms of protecting humans from malaria infection, reduced with decrease in LLIN utilization levels. Notably, under these conditions, no such advantage was registered in the two and one year maximum LLIN utilization scenarios”. For details, see pages 11, 12 and 13, lines 280 to 316.

(xvi) Comment: Line 188: I understand this was a simulation model, not an examination of effects in the real world.

Action: This was a simulated model, simulated using parameters from published literature sources, see page 8 lines 205 and 206.

(b) Reviewer Six:

(i) Comment: The terminology around LLIN 'utilization', 'coverage', and 'effective coverage' could be made more precise. Although this has improved since the original submission, these concepts remain somewhat conflated. In my understanding, since the central contribution of the paper is framed around bed net utilization, the manuscript would benefit from a clearer distinction between behavioural use, ownership/access, retention over time, and declining net performance.

Action: All confusing or conflicting terminologies have been withdrawn from the manuscript, and consistently adopted utilization (e.g LLIN or bed net utilization).

(ii) Comment: The distinction between the analytical model and the simulation framework could be made more explicit. The manuscript presents an analytical system in which parameters are treated as constant, but the simulations later introduce time-varying LLIN-related parameters and seasonal mosquito recruitment. The authors do make the distinction implicitly, in the sense that they first present model system and then, in the methods/results setup, they explicitly say that the system was numerically simulated in Python, and that for numerical simulations several LLIN-related parameters were allowed to decay exponentially over time. The authors also explicitly introduce seasonal mosquito recruitment as Λv (t) and a sigmoid function to model the growth of effective coverage after distribution, showing that the simulation layer is not identical to the simpler analytical setup. This is acceptable, but it may be stated more clearly that the theoretical analysis refers to a simplified baseline formulation, whereas the numerical simulations extend that framework to include time-dependent intervention and seasonal effects.

Action: Thank you for bringing this to our attention. The distinction between the

analytical model and the simulation framework was clarified at the start of the Model description and formulation section, explicitly stating that the analytical formulation uses constant parameters for tractable threshold analysis while the numerical simulations extend this baseline model to incorporate seasonal mosquito recruitment, fitness costs, and time dependent LLIN related parameters. For more details, see page 3, lines 57 to 67, page 8, lines 189 to 205, and line 235.

(iii) Comment: The manuscript would still benefit from a final brief language and formatting edit. Two specific points I noted are: 1) the manuscript generally uses American English (for example, "utilization"), but on page 2 there is an instance of the British spelling "utilisation", which should be standardised; 2) on page 9, Anopheles gambiae should be italicised as per standard scientific formatting.

Action: Language and formatting has been streamlined throughout the manuscript. See page 2 line 49, page 9 line 23.

Sincerely,

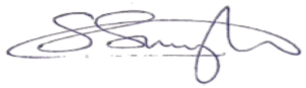

Ivan Sseguya
