# Supplementary material for: Modelling the impact of mosquito bed net utilization on malaria transmission and evolution of pyrethroid resistance
Source: PLoS One. 2026 Jul 15;21(7):e0353301. doi: 10.1371/journal.pone.0353301 (PMC13372178; doi:10.1371/journal.pone.0353301)
Supplement: Attachment — Submitted filename: Response_to_Reviewers_may_2026.pdf [file pone.0353301.s007.pdf]

May 21, 2026

Makerere University,

P.O Box 7062,

Kampala, Uganda.

Phone: +256705461622

Email: isseguya@gmail.com

Through the Academic Editor, PLOS ONE,

1875 Mission Street, Suite 103 #188,

San Francisco, CA 94103,

United States.

Dear Editor and Reviewers,

RE: Report on the Required Reviewer - Revisions

On behalf, of the authors for manuscript titled “Modelling the impact of mosquito bed net utilization on malaria transmission and evolution of pyrethroid resistance”, we thank we thank reviewers for their careful comments about our manuscript and constructive feed back provided. This manuscript has been revised in response to reviewer comments obtained as feed back and an account of these revisions is as follows:

(a) Reviewer Seven:

(i) Comment: It is good to mention the region or the country at the end of the first sentence. “………in the malaria endemic regions in ……………….” options (a) across the globe (b)African region (c) a particular country in Africa, e.g. Uganda.

Action: This sentence has been modified accordingly to “Long-lasting Insecticide Nets (LLINs) are central to malaria prevention, but their effectiveness is

threatened by the increasing pyrethroid resistance and low bed net utilization in malaria-endemic regions globally”. See page 1.

(ii) Comment: In fig 4 legend, mention separately three successive LLIN campaigns.

Action: This clarification has been made in the legend of figure 4 on page 12.

(iii) Comment: Revise the reiteration of descriptions in the Result and Discussion sections.

Action: The description of results and discussion section has been reiterated. Repetitions have been removed between the tow sections, description of results made more specific, and discussed these results in relation to existing literature. See pages 11 to 18 for description of results, and pages 18 and 19 for discussion of results.

(iv) Comment: The last sentence of the Conclusion should be removed and replaced with some recommendations to mitigate the selection pressure developed against the mosquito population due to prolonged utilization of pyrethroid-based LLINs in the Malaria control program.

Action: The last sentence under question has been dropped and recommendations based on the results of this study have been adopted. See page 20, line 530 to 535.

Sincerely,

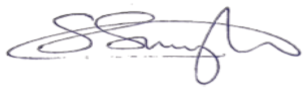

Ivan Sseguya
