# Supplementary material for: Modelling the impact of mosquito bed net utilization on malaria transmission and evolution of pyrethroid resistance
Source: PLoS One. 2026 Jul 15;21(7):e0353301. doi: 10.1371/journal.pone.0353301 (PMC13372178; doi:10.1371/journal.pone.0353301)
Supplement: Peer review history

# Peer review: Decision Letter 0

**DOI:** 10.1371/journal.pone.0353301.r001

**Authors:** Rajib Chowdhury

19 Nov 2025

PONE-D-25-51344

Modelling the Impact of Mosquito Bed Net Utilization on Malaria Dynamics and Evolution of Pyrethroid Resistance

PLOS ONE

Dear Dr. Ivan,

Thank you for submitting your manuscript to PLOS ONE. After careful consideration, we feel that it has merit but does not fully meet PLOS ONE’s publication criteria as it currently stands. Therefore, we invite you to submit a revised version of the manuscript that addresses the points raised during the review process.

Please submit your revised manuscript by Jan 03 2026 11:59PM. If you will need more time than this to complete your revisions, please reply to this message or contact the journal office at plosone@plos.org. When you're ready to submit your revision, log on to https://www.editorialmanager.com/pone/ and select the 'Submissions Needing Revision' folder to locate your manuscript file.

Please include the following items when submitting your revised manuscript:

- A rebuttal letter that responds to each point raised by the academic editor and reviewer(s). You should upload this letter as a separate file labeled 'Response to Reviewers'.
- A marked-up copy of your manuscript that highlights changes made to the original version. You should upload this as a separate file labeled 'Revised Manuscript with Track Changes'.
- An unmarked version of your revised paper without tracked changes. You should upload this as a separate file labeled 'Manuscript'.

If you would like to make changes to your financial disclosure, please include your updated statement in your cover letter. Guidelines for resubmitting your figure files are available below the reviewer comments at the end of this letter.

If applicable, we recommend that you deposit your laboratory protocols in protocols.io to enhance the reproducibility of your results. Protocols.io assigns your protocol its own identifier (DOI) so that it can be cited independently in the future. For instructions see: https://journals.plos.org/plosone/s/submission-guidelines#loc-laboratory-protocols. Additionally, PLOS ONE offers an option for publishing peer-reviewed Lab Protocol articles, which describe protocols hosted on protocols.io. Read more information on sharing protocols at https://plos.org/protocols?utm_medium=editorial-email&utm_source=authorletters&utm_campaign=protocols.

We look forward to receiving your revised manuscript.

Kind regards,

Rajib Chowdhury, M.Sc.; MPH

Academic Editor

PLOS ONE

Journal Requirements:

When submitting your revision, we need you to address these additional requirements.

1. Please ensure that your manuscript meets PLOS ONE's style requirements, including those for file naming. The PLOS ONE style templates can be found at

https://journals.plos.org/plosone/s/file?id=wjVg/PLOSOne_formatting_sample_main_body.pdf and https://journals.plos.org/plosone/s/file?id=ba62/PLOSOne_formatting_sample_title_authors_affiliations.pdf

2. Please note that PLOS One has specific guidelines on code sharing for submissions in which author-generated code underpins the findings in the manuscript. In these cases, we expect all author-generated code to be made available without restrictions upon publication of the work. Please review our guidelines at https://journals.plos.org/plosone/s/materials-and-software-sharing#loc-sharing-code and ensure that your code is shared in a way that follows best practice and facilitates reproducibility and reuse.

3. Please update your submission to use the PLOS LaTeX template. The template and more information on our requirements for LaTeX submissions can be found at http://journals.plos.org/plosone/s/latex.

4. Thank you for stating the following in the Acknowledgments Section of your manuscript:

[This study was supported by the African Consortium in Modelling for Effective Vector Control (ACoMVeC) project INV-047049. However, the sponsors did not have any role in the study design, choice of parametrization, analysis, or manuscript preparation and publication.]

We note that you have provided funding information that is not currently declared in your Funding Statement. However, funding information should not appear in the Acknowledgments section or other areas of your manuscript. We will only publish funding information present in the Funding Statement section of the online submission form.

Please remove any funding-related text from the manuscript and let us know how you would like to update your Funding Statement. Currently, your Funding Statement reads as follows:

[The author(s) received no specific funding for this work.]

Please include your amended statements within your cover letter; we will change the online submission form on your behalf.

5. We note that your Data Availability Statement is currently as follows: [All relevant data are within the manuscript and its Supporting Information files.]

Please confirm at this time whether or not your submission contains all raw data required to replicate the results of your study. Authors must share the “minimal data set” for their submission. PLOS defines the minimal data set to consist of the data required to replicate all study findings reported in the article, as well as related metadata and methods (https://journals.plos.org/plosone/s/data-availability#loc-minimal-data-set-definition).

For example, authors should submit the following data:

- The values behind the means, standard deviations and other measures reported;

- The values used to build graphs;

- The points extracted from images for analysis.

Authors do not need to submit their entire data set if only a portion of the data was used in the reported study.

If your submission does not contain these data, please either upload them as Supporting Information files or deposit them to a stable, public repository and provide us with the relevant URLs, DOIs, or accession numbers. For a list of recommended repositories, please see https://journals.plos.org/plosone/s/recommended-repositories.

If there are ethical or legal restrictions on sharing a de-identified data set, please explain them in detail (e.g., data contain potentially sensitive information, data are owned by a third-party organization, etc.) and who has imposed them (e.g., an ethics committee). Please also provide contact information for a data access committee, ethics committee, or other institutional body to which data requests may be sent. If data are owned by a third party, please indicate how others may request data access.

6. Please amend the manuscript submission data (via Edit Submission) to include authors Prashanth Selvarajb and Jonathan Kayondo.

7. Please remove your figures from within your manuscript file, leaving only the individual TIFF/EPS image files, uploaded separately. These will be automatically included in the reviewers’ PDF.

If the reviewer comments include a recommendation to cite specific previously published works, please review and evaluate these publications to determine whether they are relevant and should be cited. There is no requirement to cite these works unless the editor has indicated otherwise.

[Note: HTML markup is below. Please do not edit.]

Reviewers' comments:

Reviewer's Responses to Questions

**Comments to the Author**

1. Is the manuscript technically sound, and do the data support the conclusions?

The manuscript must describe a technically sound piece of scientific research with data that supports the conclusions. Experiments must have been conducted rigorously, with appropriate controls, replication, and sample sizes. The conclusions must be drawn appropriately based on the data presented.

Reviewer #1: Partly

Reviewer #2: Yes

Reviewer #3: Yes

**********

2. Has the statistical analysis been performed appropriately and rigorously?

Reviewer #1: I Don't Know

Reviewer #2: Yes

Reviewer #3: Yes

**********

3. Have the authors made all data underlying the findings in their manuscript fully available?

The PLOS Data policy requires authors to make all data underlying the findings described in their manuscript fully available without restriction, with rare exception (please refer to the Data Availability Statement in the manuscript PDF file). The data should be provided as part of the manuscript or its supporting information, or deposited to a public repository. For example, in addition to summary statistics, the data points behind means, medians and variance measures should be available. If there are restrictions on publicly sharing data—e.g. participant privacy or use of data from a third party—those must be specified.

Reviewer #1: No

Reviewer #2: Yes

Reviewer #3: Yes

**********

4. Is the manuscript presented in an intelligible fashion and written in standard English?

PLOS ONE does not copyedit accepted manuscripts, so the language in submitted articles must be clear, correct, and unambiguous. Any typographical or grammatical errors should be corrected at revision, so please note any specific errors here.

Reviewer #1: No

Reviewer #2: Yes

Reviewer #3: Yes

**********

5. Review Comments to the Author

Please use the space provided to explain your answers to the questions above. You may also include additional comments for the author, including concerns about dual publication, research ethics, or publication ethics. (Please upload your review as an attachment if it exceeds 20,000 characters)

Reviewer #1: Modelling the Impact of Mosquito Bed Net Utilization on Malaria Dynamics and Evolution of Pyrethroid Resistance

Abstract:

Could the authors kindly define what they mean with ‘Malaria Dynamics’ for clarity.

From the abstract: “This study formulates a genotype-specific compartmental model accounting for malaria transmission between the human and vector populations.” From the epidemiological model, for a successful malaria transmission, three things come into play: Host, Agent and environment. Were this accounted for by the model?

“It incorporates the blocking and insecticidal killing effects of LLINs, and pyrethroid-induced fitness costs, to evaluate the impact of LLIN utilization on malaria and resistance dynamics.” How was this done? Blocking effect is as a result of fabric integrity, killing effect is all about the chemical/bioefficacy effect. Why did the authors look at the pyrethroid fitness costs, we are aware that pyrethroids are the cheapest forms of insecticides used for Longlasting insecticidal nets (LLINs) protection. Do we have any comparative parameter for this aspect in this study?

“Reproductive numbers, with bed net usage RL 0 and without bed net usage R0, were calculated and sensitivity analysis conducted.” What is Reproductive numbers in this case? According to WHO, bed net usage is an individual having slept under a net the previous night prior the survey. Was this considered by the model? And how was sensitive analysis done? Ideally, at this point what I mean is that the authors must make it very clear, in a manner that readers of same and different backgrounds can understand without specific scientific training. Now that the study looks/is determined towards public health impact, can policy makers understand the take-home messages if they were to be guided by WHO guidelines?

The title of the manuscript is “Modelling the Impact of Mosquito Bed Net Utilization on Malaria Dynamics and Evolution of Pyrethroid Resistance”, yet we see the authors in the results section of abstract talk about LLIN coverage. Coverage is different from utilization. What do the authors mean with “highest” in “highest effect in reducing malaria transmission”, what was the measure or level?

Please the authors need to take note of this: Despite of an initial LLIN coverage of 81% (Where?/When? How was this obtained?), without consistent utilization (What is the meaning of this? What was the measure? How do we define consistent usage?), protection (measure of protection has not been provided please or talked about in the abstract) against malaria infection from pyrethroid only and pyrethroid piperonyl butoxide (PBO) nets, was not sustained over time (What was the timeline?). PBO (Start a sentence always in full not abbreviations-This needs also to be considered throughout the manuscript text) nets averted more infections and suppressed malaria vectors by 72% (does this account for the parasite and the vector?) under standard utilization (What is the meaning of standard utilization), although this effectiveness declined with shorter retention periods. Also, the study showed that resistance evolves faster with repeated pyrethroid-only campaigns, but PBO nets negate this trend especially when such resistance carries a higher fitness cost. While shorter LLIN retention slows the evolution of pyrethroid resistance, it is not a viable option for malaria. Do the authors imply that LLINs may not offer substantial protection? Was there a control to make this conclusion such as the use of indoor residual spraying (IRS)?

Reviewer #2: The proposed model advances beyond traditional SIR/SEIR frameworks by employing a highly detailed disaggregation:

Vector Population (Mosquito): The vector population not only tracks the epidemiological state (Susceptible, Exposed/Latently Infected, Infectious – an SEI model) but also incorporates three distinct genotypes within each state (SS, SR, RR). This results in nine vector compartments, which is essential for accurately modeling the population genetics of resistance.

Logistic Recruitment: Vector growth is modeled using a logistic function ($\Lambda_v N_v (1 - N_v/K)$), thereby integrating the environmental carrying capacity ($K$). This formulation is more biologically realistic than simple exponential growth.

Hardy-Weinberg Gene Frequencies: The recruitment of new mosquitoes is based on the allelic frequencies ($\mathbf{p^2, 2pq, q^2}$), ensuring that the inheritance of resistance is modeled in a genetically correct manner (under the premise of random mating, typical in the absence of mating selection).

The methodology is highly appropriate for the stated objective. The disaggregation of the mosquito population by epidemiological state (SEI) and genotype (SS, SR, RR), coupled with the direct inclusion of bed net efficacy parameters ($\mathbf{LLINbloc}$ and $\mathbf{LLINeff}$), establishes a comprehensive mathematical framework for investigating the dynamics and evolution of resistance. The study is well-positioned to yield valuable insights into the trade-offs between malaria control and the accelerated evolution of pyrethroid resistance.

The model results provide crucial evidence to improve intervention strategies:

Emphasis on Effective Utilization, Not Just Coverage (Lines 340-345): The model demonstrates that high initial coverage (81%) is insufficient if consistent utilization and the net retention period are short (less than 16 months).

Gain: Public health authorities should reorient their focus from distribution indicators to behavioral and durability metrics. Investing in continuous health education for correct use and timely net replacement is shown to be more effective than solely relying on large initial distribution campaigns.

Evidence for PBO Nets (Lines 355-360): The results validate the use of Pyrethroid-PBO nets as a strategic tool to reverse the resistance trend (by reducing the amplification of heterozygous genotypes).

Gain: The model offers the technical justification for local managers to prioritize the purchase and deployment of next-generation nets (PBO or Chlorfenapyr) in areas where conventional pyrethroid resistance is already established or high. The higher cost of these nets is justified by the gain in vector suppression efficacy (72% versus pyrethroid-only nets).

The study findings can be directly applied to the strategic planning of vector surveillance:

Basis for Insecticide Rotation (Lines 365-369): The study suggests net rotation as a Resistance Management strategy.

Local Application: Before starting a new distribution campaign, the municipality should perform local vector susceptibility/resistance testing (bioassays). If resistance is high, the decision should be to rotate towards PBO or other enhanced nets, complementing these efforts with Larval Source Management (LSM) and Indoor Residual Spraying (IRS) in high-risk areas (hotspots).

Addressing Heterogeneity and Hotspots (Lines 370-371): Although the model assumes homogeneous mixing, the discussion correctly identifies heterogeneity and hotspots as limitations.

Local Application: Managers should utilize Geographic Information Systems (GIS) to map areas of high incidence and low net utilization. The response must be focalized, directing the more expensive interventions (IRS, PBO) and enhanced education toward these critical areas to optimize spending.

Revision of Net Replacement Schedules (Lines 350-352): The observation that entomological impact is negated within 16 months under inconsistent usage challenges the typical 3-year replacement cycle.

Local Application: The municipality should consider substituting and repairing nets on a shorter cycle (12-18 months) in communities with low retention or high physical damage, ensuring that effective coverage does not drop prematurely.

Reviewer #3: Abstract: Line 10: remove “of” in “Despite of an initial LLIN…”.

Line 7: the quote of 2793 deaths in 2023 related to malaria seems low. I believe the estimate was over 16,000, but official records were 2793. Please incorporate additional information in the deaths related to malaria.

Line 9: “20% of hospital deaths… “ are due to malaria?! 2793 deaths seems low for a population of 50 million.

Line 17: please describe a value associated with “highly effective”.

Line 20: Can you be more specific with the amount of reduction in malaria morbidity?

Line 79: change Mosquito in “The Mosquito population” to lowercase.

Line 82: change Malaria Transmission in “The Malaria Transmission” to lowercase.

Line 120: you should describe the study in the methods section, not in the results of the study section.

Line 123: Please be more descriptive, does the efficacy of the bednets diminish from 100% to 1% over a year, 2 years, and 3 years, but the nets are replaced yearly?

Line 129-130: Please describe in section 4 and/or the methods section that the model parameters have references for their selection listed in Table 1.

Line 233: Not sure what “Susceptible pyrethroid-resistant” means… does that mean they are susceptible to malaria?

Line 281-284: the phrasing here is inaccurate. PBO cannot restore pyrethroid sensitivity in mosquitoes. It acts as a synergist to suppress the detoxification enzymes rendering the resistant mosquitoes susceptible to pyrethroids they would normally be resistant to. Unfortunately, metabolic resistance is not the only form of insecticide resistance in mosquitoes and these model results may be misleading.

Line 303 and 320: What is the standard utilization scenario?

Line 328: What are the fitness costs found in nature?

Line 366-367: You only assessed pyrethrin and PBO for the third year, how can you compare pbo with the other synergists/AI’s in this list?

General:

Please be sure to describe your tables and figures in a way that if they were separated from the main body of the manuscript, they could stand on their own. As they are currently written they cannot. For example Fig. 3 caption: “Effect of reducing LLIN retention periods of malaria dynamics across three bed net campaigns.” This makes no reference to the fact that these are simulated values generated by a model, and not actual values from a real-world experiment.

Be sure to consider using colors and patterns in your figures that are better for color blind readers.

**********

6. PLOS authors have the option to publish the peer review history of their article (what does this mean?). If published, this will include your full peer review and any attached files.

If you choose “no”, your identity will remain anonymous but your review may still be made public.

**Do you want your identity to be public for this peer review?**  For information about this choice, including consent withdrawal, please see our Privacy Policy.

Reviewer #1: No

Reviewer #2: No

Reviewer #3: No

**********

[NOTE: If reviewer comments were submitted as an attachment file, they will be attached to this email and accessible via the submission site. Please log into your account, locate the manuscript record, and check for the action link "View Attachments". If this link does not appear, there are no attachment files.]

To ensure your figures meet our technical requirements, please review our figure guidelines: https://journals.plos.org/plosone/s/figures

You may also use PLOS’s free figure tool, NAAS, to help you prepare publication quality figures: https://journals.plos.org/plosone/s/figures#loc-tools-for-figure-preparation.

NAAS will assess whether your figures meet our technical requirements by comparing each figure against our figure specifications.

Attachment Submitted filename: PONE-D-25-51344_reviewer.pdf

# Author comment: Author response to Decision Letter 1

**DOI:** 10.1371/journal.pone.0353301.r002

2 Jan 2026

All the required responses to reviewers have been reported in the "Response to Reviewer" letter attached to this resubmission.

Attachment Submitted filename: Response to Reviewers.pdf

# Peer review: Decision Letter 1

**DOI:** 10.1371/journal.pone.0353301.r003

**Authors:** Rajib Chowdhury

1 Feb 2026

PONE-D-25-51344R1

Modelling the Impact of Mosquito Bed Net Utilization on Malaria Transmission and Evolution of Pyrethroid Resistance

PLOS One

Dear Dr. Ivan,

Thank you for submitting your manuscript to PLOS ONE. After careful consideration, we feel that it has merit but does not fully meet PLOS ONE’s publication criteria as it currently stands. Therefore, we invite you to submit a revised version of the manuscript that addresses the points raised during the review process.

Please submit your revised manuscript by Mar 18 2026 11:59PM. If you will need more time than this to complete your revisions, please reply to this message or contact the journal office at plosone@plos.org. When you're ready to submit your revision, log on to https://www.editorialmanager.com/pone/ and select the 'Submissions Needing Revision' folder to locate your manuscript file.

Please include the following items when submitting your revised manuscript:

- A letter that responds to each point raised by the academic editor and reviewer(s). You should upload this letter as a separate file labeled 'Response to Reviewers'.
- A marked-up copy of your manuscript that highlights changes made to the original version. You should upload this as a separate file labeled 'Revised Manuscript with Track Changes'.
- An unmarked version of your revised paper without tracked changes. You should upload this as a separate file labeled 'Manuscript'.

-->If you would like to make changes to your financial disclosure, please include your updated statement in your cover letter. Guidelines for resubmitting your figure files are available below the reviewer comments at the end of this letter.

If applicable, we recommend that you deposit your laboratory protocols in protocols.io to enhance the reproducibility of your results. Protocols.io assigns your protocol its own identifier (DOI) so that it can be cited independently in the future. For instructions see: https://journals.plos.org/plosone/s/submission-guidelines#loc-laboratory-protocols. Additionally, PLOS ONE offers an option for publishing peer-reviewed Lab Protocol articles, which describe protocols hosted on protocols.io. Read more information on sharing protocols at https://plos.org/protocols?utm_medium=editorial-email&utm_source=authorletters&utm_campaign=protocols.

We look forward to receiving your revised manuscript.

Kind regards,

Rajib Chowdhury, M.Sc.; MPH

Academic Editor

PLOS One

Journal Requirements:

1. If the reviewer comments include a recommendation to cite specific previously published works, please review and evaluate these publications to determine whether they are relevant and should be cited. There is no requirement to cite these works unless the editor has indicated otherwise.

2. Please review your reference list to ensure that it is complete and correct. If you have cited papers that have been retracted, please include the rationale for doing so in the manuscript text, or remove these references and replace them with relevant current references. Any changes to the reference list should be mentioned in the rebuttal letter that accompanies your revised manuscript. If you need to cite a retracted article, indicate the article’s retracted status in the References list and also include a citation and full reference for the retraction notice.

[Note: HTML markup is below. Please do not edit.]

Reviewers' comments:

Reviewer's Responses to Questions

**Comments to the Author**

1. If the authors have adequately addressed your comments raised in a previous round of review and you feel that this manuscript is now acceptable for publication, you may indicate that here to bypass the “Comments to the Author” section, enter your conflict of interest statement in the “Confidential to Editor” section, and submit your "Accept" recommendation.

Reviewer #1: All comments have been addressed

Reviewer #4: All comments have been addressed

**********

2. Is the manuscript technically sound, and do the data support the conclusions?

The manuscript must describe a technically sound piece of scientific research with data that supports the conclusions. Experiments must have been conducted rigorously, with appropriate controls, replication, and sample sizes. The conclusions must be drawn appropriately based on the data presented.

Reviewer #1: Yes

Reviewer #4: Yes

**********

3. Has the statistical analysis been performed appropriately and rigorously?

Reviewer #1: Yes

Reviewer #4: Yes

**********

4. Have the authors made all data underlying the findings in their manuscript fully available?

The PLOS Data policy requires authors to make all data underlying the findings described in their manuscript fully available without restriction, with rare exception (please refer to the Data Availability Statement in the manuscript PDF file). The data should be provided as part of the manuscript or its supporting information, or deposited to a public repository. For example, in addition to summary statistics, the data points behind means, medians and variance measures should be available. If there are restrictions on publicly sharing data—e.g. participant privacy or use of data from a third party—those must be specified.

Reviewer #1: Yes

Reviewer #4: Yes

**********

5. Is the manuscript presented in an intelligible fashion and written in standard English?

PLOS ONE does not copyedit accepted manuscripts, so the language in submitted articles must be clear, correct, and unambiguous. Any typographical or grammatical errors should be corrected at revision, so please note any specific errors here.

Reviewer #1: Yes

Reviewer #4: Yes

**********

6. Review Comments to the Author

Please use the space provided to explain your answers to the questions above. You may also include additional comments for the author, including concerns about dual publication, research ethics, or publication ethics. (Please upload your review as an attachment if it exceeds 20,000 characters)

Reviewer #1: The authors have taken time and keenly handled my comments. I wish to thank them and the editor of this journal.

Reviewer #4: Please correct the following mistakes:

1. There is no uniformity for WHO. for example Ref no 43. Organization WH. But in all other references it is WHO.

2. In reference no. 71. Neither Author/s nor publisher have been mentioned.

**********

7. PLOS authors have the option to publish the peer review history of their article (what does this mean?). If published, this will include your full peer review and any attached files.

If you choose “no”, your identity will remain anonymous but your review may still be made public.

**Do you want your identity to be public for this peer review?**  For information about this choice, including consent withdrawal, please see our Privacy Policy.

Reviewer #1: No

Reviewer #4: No

**********

[NOTE: If reviewer comments were submitted as an attachment file, they will be attached to this email and accessible via the submission site. Please log into your account, locate the manuscript record, and check for the action link "View Attachments". If this link does not appear, there are no attachment files.]

To ensure your figures meet our technical requirements, please review our figure guidelines: https://journals.plos.org/plosone/s/figures

You may also use PLOS’s free figure tool, NAAS, to help you prepare publication quality figures: https://journals.plos.org/plosone/s/figures#loc-tools-for-figure-preparation.

NAAS will assess whether your figures meet our technical requirements by comparing each figure against our figure specifications.

# Author comment: Author response to Decision Letter 2

**DOI:** 10.1371/journal.pone.0353301.r004

10 Feb 2026

All reviews have been addressed accordingly as indicated in the "Response to Reviewers" file.

Attachment Submitted filename: Response_to_Reviewers_auresp_2.pdf

# Peer review: Decision Letter 2

**DOI:** 10.1371/journal.pone.0353301.r005

**Authors:** Rajib Chowdhury

16 Mar 2026

PONE-D-25-51344R2

Modelling the Impact of Mosquito Bed Net Utilization on Malaria Transmission and Evolution of Pyrethroid Resistance

PLOS One

Dear Dr. Ivan,

Thank you for submitting your manuscript to PLOS ONE. After careful consideration, we feel that it has merit but does not fully meet PLOS ONE’s publication criteria as it currently stands. Therefore, we invite you to submit a revised version of the manuscript that addresses the points raised during the review process.

Please submit your revised manuscript by Apr 30 2026 11:59PM. If you will need more time than this to complete your revisions, please reply to this message or contact the journal office at plosone@plos.org. When you're ready to submit your revision, log on to https://www.editorialmanager.com/pone/ and select the 'Submissions Needing Revision' folder to locate your manuscript file.

Please include the following items when submitting your revised manuscript:

- A letter that responds to each point raised by the academic editor and reviewer(s). You should upload this letter as a separate file labeled 'Response to Reviewers'.
- A marked-up copy of your manuscript that highlights changes made to the original version. You should upload this as a separate file labeled 'Revised Manuscript with Track Changes'.
- An unmarked version of your revised paper without tracked changes. You should upload this as a separate file labeled 'Manuscript'.

-->If you would like to make changes to your financial disclosure, please include your updated statement in your cover letter. Guidelines for resubmitting your figure files are available below the reviewer comments at the end of this letter.

If applicable, we recommend that you deposit your laboratory protocols in protocols.io to enhance the reproducibility of your results. Protocols.io assigns your protocol its own identifier (DOI) so that it can be cited independently in the future. For instructions see: https://journals.plos.org/plosone/s/submission-guidelines#loc-laboratory-protocols. Additionally, PLOS ONE offers an option for publishing peer-reviewed Lab Protocol articles, which describe protocols hosted on protocols.io. Read more information on sharing protocols at https://plos.org/protocols?utm_medium=editorial-email&utm_source=authorletters&utm_campaign=protocols.

We look forward to receiving your revised manuscript.

Kind regards,

Rajib Chowdhury, M.Sc.; MPH

Academic Editor

PLOS One

**Journal Requirements:**

1. If the reviewer comments include a recommendation to cite specific previously published works, please review and evaluate these publications to determine whether they are relevant and should be cited. There is no requirement to cite these works unless the editor has indicated otherwise.

2. Please review your reference list to ensure that it is complete and correct. If you have cited papers that have been retracted, please include the rationale for doing so in the manuscript text, or remove these references and replace them with relevant current references. Any changes to the reference list should be mentioned in the rebuttal letter that accompanies your revised manuscript. If you need to cite a retracted article, indicate the article’s retracted status in the References list and also include a citation and full reference for the retraction notice.

[Note: HTML markup is below. Please do not edit.]

Reviewers' comments:

Reviewer's Responses to Questions

**Comments to the Author**

1. If the authors have adequately addressed your comments raised in a previous round of review and you feel that this manuscript is now acceptable for publication, you may indicate that here to bypass the “Comments to the Author” section, enter your conflict of interest statement in the “Confidential to Editor” section, and submit your "Accept" recommendation.

Reviewer #1: All comments have been addressed

Reviewer #5: All comments have been addressed

Reviewer #6: All comments have been addressed

**********

2. Is the manuscript technically sound, and do the data support the conclusions?

The manuscript must describe a technically sound piece of scientific research with data that supports the conclusions. Experiments must have been conducted rigorously, with appropriate controls, replication, and sample sizes. The conclusions must be drawn appropriately based on the data presented.

Reviewer #1: Yes

Reviewer #5: Yes

Reviewer #6: Yes

**********

3. Has the statistical analysis been performed appropriately and rigorously?

Reviewer #1: Yes

Reviewer #5: Yes

Reviewer #6: I Don't Know

**********

4. Have the authors made all data underlying the findings in their manuscript fully available?

The PLOS Data policy requires authors to make all data underlying the findings described in their manuscript fully available without restriction, with rare exception (please refer to the Data Availability Statement in the manuscript PDF file). The data should be provided as part of the manuscript or its supporting information, or deposited to a public repository. For example, in addition to summary statistics, the data points behind means, medians and variance measures should be available. If there are restrictions on publicly sharing data—e.g. participant privacy or use of data from a third party—those must be specified.

Reviewer #1: Yes

Reviewer #5: Yes

Reviewer #6: Yes

**********

5. Is the manuscript presented in an intelligible fashion and written in standard English?

PLOS ONE does not copyedit accepted manuscripts, so the language in submitted articles must be clear, correct, and unambiguous. Any typographical or grammatical errors should be corrected at revision, so please note any specific errors here.

Reviewer #1: Yes

Reviewer #5: Yes

Reviewer #6: Yes

**********

6. Review Comments to the Author

Please use the space provided to explain your answers to the questions above. You may also include additional comments for the author, including concerns about dual publication, research ethics, or publication ethics. (Please upload your review as an attachment if it exceeds 20,000 characters)

**Reviewer #1:**  I wish to thank the authors for considering and addressing my comments in order to improve their manuscript.

**Reviewer #5:**  Thank you for this work. A few minor thoughts and suggestions:

Line 18: Instead of "systematic review", should state "meta-analysis", because you are reporting the numerical calculations, not the literature review portion

Line 21: aversion of "68% of 663 million malaria cases registered" does not make sense, because these were reported cases. Please revise your wording for clarity.

Lines 42 and 43: For clarity, "bed net utilization" should read "bed net retention time per campaign"

Lines 96-100All these "assumptions" are limitations which should be discussed in further detail in the discussion

Line 101: Modelling recruitment of new mosquitoes is defined on line 86, but not clear here, where logistic term A is discussed

Line 115: Please explain epidemiological feasible region in words and describe its implication in this research. Also direct readers to the Appendix for equation and further understanding.

Line 183: The way you decided upon rate of decay of bed net use was not clear and produced some questionability in my mind of major findings, until I dug deeper into the references you had used. I think more communication describing these decisions would help manuscript narrative.

Line 186: Remove the phrase "On the hand," completely. It is not grammatically correct and does not lend information or context.

Lines 219-226: The utility of this paragraph is misleading, since the model assumes no fitness costs are incurred by malaria vectors (line 126).

Line 232: Partial Rank Correlation techniques were not discussed in the manuscript

Lines 241-242: These results highlight the importance of LLINcov and LLINeffr, and the narrative surrounding modelling and mathematical decisions surrounding these variables should be clear and sound throughout the manuscript.

Line 243: Word "this" should be removed

Overall, the way model results read should be clear that this was a simulation and these are results OF THIS STUDY, not a global truth. Using present tense language makes it appear as a generalizable truth instead of simply reporting your results. This is consistent throughout the manuscript and should be revised for clarity.

Lines 244-246: The way that "rate of parasite development in vectors" is repeated reads confusingly. Additionally, clarify that increase in theta had the smallest effect OF THE INCLUDED PARAMETERS on malaria transmission.

Lines 263-264 and Fig 3: Range of suppression of malaria cases from 0 to close to 100% appears strikingly unrealistic

Lines 266-267: As in the rest of the manuscript, results should be presented in past tense, not present tense

Lines 282-284: Please re-evaluate and rephrase this section for clarity, without speculation. No advantage was registered from the use of pyrethroid PBO nets during the third campaign? I see that each bednet campaign was expected to last a period of three years, and pyrethroid-PBO nets were introduced in the third campaign, at year 7 of the simulation. Line 277 states that PBO LLINs prevent more malaria infections due to ability to kill sensitive and resistant vectors. Line 314 reports that PBO nets reduced mosquitoes to near zero for the most of the campaign period, Please re-analyze and clarify.

Line 188: I understand this was a simulation model, not an examination of effects in the real world.

Line 434: partial rank correlation coefficient analysis was not discussed in the manuscript

**Reviewer #6:**  I have carefully reviewed the manuscript, including the revised versions, and I appreciate the substantial effort made by the authors to improve the work.

The manuscript has a clear and relevant rationale: it tries to simultaneously model malaria transmission and the evolution of Pyrethroid resistance under different patterns of Long-lasting Insecticide Net (LLIN) utilization, including a third campaign using pyrethroid-piperonyl butoxide (PBO)-treated nets.

After the first two rounds of reviews, the manuscript is more organised, the current title is more aligned with the work produced, the introduction is more up-to-date with recent literature and well-framed regarding the current situation in Uganda, and the novelty of the model is better presented. The objective is well explained, and the results section is easier to follow, considering that the authors made an effort to better describe the different study scenarios before presenting the outputs.

Overall, I consider this study relevant, as it addresses an important question at the interface of malaria transmission dynamics, vector control, and insecticide resistance. The inclusion of repeated campaigns and a later PBO-based intervention adds practical interest to the analysis.

Nevertheless, although the manuscript has improved markedly, I believe a few issues could still benefit from clarification before acceptance. My current recommendation would be minor revision.

My remaining comments are as follows:

1. The terminology around LLIN ‘utilization’, ‘coverage’, and ‘effective coverage’ could be made more precise. Although this has improved since the original submission, these concepts remain somewhat conflated. In my understanding, since the central contribution of the paper is framed around bed net utilization, the manuscript would benefit from a clearer distinction between behavioural use, ownership/access, retention over time, and declining net performance.

2. The distinction between the analytical model and the simulation framework could be made more explicit. The manuscript presents an analytical system in which parameters are treated as constant, but the simulations later introduce time-varying LLIN-related parameters and seasonal mosquito recruitment. The authors do make the distinction implicitly, in the sense that they first present model system and then, in the methods/results setup, they explicitly say that the system was numerically simulated in Python, and that for numerical simulations several LLIN-related parameters were allowed to decay exponentially over time. The authors also explicitly introduce seasonal mosquito recruitment as Λv (t) and a sigmoid function to model the growth of effective coverage after distribution, showing that the simulation layer is not identical to the simpler analytical setup. This is acceptable, but it may be stated more clearly that the theoretical analysis refers to a simplified baseline formulation, whereas the numerical simulations extend that framework to include time-dependent intervention and seasonal effects.

3. The manuscript would still benefit from a final brief language and formatting edit. Two specific points I noted are: 1) the manuscript generally uses American English (for example, “utilization”), but on page 2 there is an instance of the British spelling “utilisation”, which should be standardised; 2) on page 9, Anopheles gambiae should be italicised as per standard scientific formatting.

The manuscript has clearly improved across revisions, and I appreciate the authors’ efforts in addressing earlier concerns. I believe the remaining issues are limited and addressable without major restructuring.

I have not commented here on the statistical analysis in detail, as this lies outside my main area of expertise.

**********

7. PLOS authors have the option to publish the peer review history of their article (what does this mean?). If published, this will include your full peer review and any attached files.

If you choose “no”, your identity will remain anonymous but your review may still be made public.

**Do you want your identity to be public for this peer review?**  For information about this choice, including consent withdrawal, please see our Privacy Policy.

Reviewer #1: No

Reviewer #5: No

Reviewer #6: No

**********

[NOTE: If reviewer comments were submitted as an attachment file, they will be attached to this email and accessible via the submission site. Please log into your account, locate the manuscript record, and check for the action link "View Attachments". If this link does not appear, there are no attachment files.]

To ensure your figures meet our technical requirements, please review our figure guidelines: https://journals.plos.org/plosone/s/figures

You may also use PLOS’s free figure tool, NAAS, to help you prepare publication quality figures: https://journals.plos.org/plosone/s/figures#loc-tools-for-figure-preparation.

NAAS will assess whether your figures meet our technical requirements by comparing each figure against our figure specifications.

# Author comment: Author response to Decision Letter 3

**DOI:** 10.1371/journal.pone.0353301.r006

10 Apr 2026

All reviews have been attended to and a detailed report, under the file name "Response to Reviewers_march26", has been attached in the uploaded files.

Attachment Submitted filename: Response to Reviewers_march26.pdf

# Peer review: Decision Letter 3

**DOI:** 10.1371/journal.pone.0353301.r007

**Authors:** Rajib Chowdhury

12 May 2026

PONE-D-25-51344R3

Modelling the Impact of Mosquito Bed Net Utilization on Malaria Transmission and Evolution of Pyrethroid Resistance

PLOS One

Dear Dr. Ivan,

Thank you for submitting your manuscript to PLOS ONE. After careful consideration, we feel that it has merit but does not fully meet PLOS ONE’s publication criteria as it currently stands. Therefore, we invite you to submit a revised version of the manuscript that addresses the points raised during the review process.

Please submit your revised manuscript by Jun 26 2026 11:59PM. If you will need more time than this to complete your revisions, please reply to this message or contact the journal office at plosone@plos.org. When you're ready to submit your revision, log on to https://www.editorialmanager.com/pone/ and select the 'Submissions Needing Revision' folder to locate your manuscript file.

Please include the following items when submitting your revised manuscript:

- A letter that responds to each point raised by the academic editor and reviewer(s). You should upload this letter as a separate file labeled 'Response to Reviewers'.
- A marked-up copy of your manuscript that highlights changes made to the original version. You should upload this as a separate file labeled 'Revised Manuscript with Track Changes'.
- An unmarked version of your revised paper without tracked changes. You should upload this as a separate file labeled 'Manuscript'.

If you would like to make changes to your financial disclosure, please include your updated statement in your cover letter. Guidelines for resubmitting your figure files are available below the reviewer comments at the end of this letter.

If applicable, we recommend that you deposit your laboratory protocols in protocols.io to enhance the reproducibility of your results. Protocols.io assigns your protocol its own identifier (DOI) so that it can be cited independently in the future. For instructions see: https://journals.plos.org/plosone/s/submission-guidelines#loc-laboratory-protocols. Additionally, PLOS ONE offers an option for publishing peer-reviewed Lab Protocol articles, which describe protocols hosted on protocols.io. Read more information on sharing protocols at https://plos.org/protocols?utm_medium=editorial-email&utm_source=authorletters&utm_campaign=protocols.

As the corresponding author, your ORCID iD is verified in the submission system and will appear in the published article. PLOS supports the use of ORCID, and we encourage all coauthors to register for an ORCID iD and use it as well. Please encourage your coauthors to verify their ORCID iD within the submission system before final acceptance, as unverified ORCID iDs will not appear in the published article. *Only*  the individual author can complete the verification step; PLOS staff *cannot*  verify ORCID iDs on behalf of authors.

We look forward to receiving your revised manuscript.

Kind regards,

Rajib Chowdhury, M.Sc.; MPH

Academic Editor

PLOS One

Journal Requirements:

If the reviewer comments include a recommendation to cite specific previously published works, please review and evaluate these publications to determine whether they are relevant and should be cited. There is no requirement to cite these works unless the editor has indicated otherwise.

Please review your reference list to ensure that it is complete and correct. If you have cited papers that have been retracted, please include the rationale for doing so in the manuscript text, or remove these references and replace them with relevant current references. Any changes to the reference list should be mentioned in the rebuttal letter that accompanies your revised manuscript. If you need to cite a retracted article, indicate the article’s retracted status in the References list and also include a citation and full reference for the retraction notice.

[Note: HTML markup is below. Please do not edit.]

Reviewers' comments:

Reviewer's Responses to Questions

**Comments to the Author**

1. If the authors have adequately addressed your comments raised in a previous round of review and you feel that this manuscript is now acceptable for publication, you may indicate that here to bypass the “Comments to the Author” section, enter your conflict of interest statement in the “Confidential to Editor” section, and submit your "Accept" recommendation.

Reviewer #6: All comments have been addressed

Reviewer #7: (No Response)

**********

2. Is the manuscript technically sound, and do the data support the conclusions?

The manuscript must describe a technically sound piece of scientific research with data that supports the conclusions. Experiments must have been conducted rigorously, with appropriate controls, replication, and sample sizes. The conclusions must be drawn appropriately based on the data presented.

Reviewer #6: Yes

Reviewer #7: Yes

**********

3. Has the statistical analysis been performed appropriately and rigorously?

Reviewer #6: N/A

Reviewer #7: Yes

**********

4. Have the authors made all data underlying the findings in their manuscript fully available?

The PLOS Data policy requires authors to make all data underlying the findings described in their manuscript fully available without restriction, with rare exception (please refer to the Data Availability Statement in the manuscript PDF file). The data should be provided as part of the manuscript or its supporting information, or deposited to a public repository. For example, in addition to summary statistics, the data points behind means, medians and variance measures should be available. If there are restrictions on publicly sharing data—e.g. participant privacy or use of data from a third party—those must be specified.

Reviewer #6: Yes

Reviewer #7: Yes

**********

5. Is the manuscript presented in an intelligible fashion and written in standard English?

PLOS ONE does not copyedit accepted manuscripts, so the language in submitted articles must be clear, correct, and unambiguous. Any typographical or grammatical errors should be corrected at revision, so please note any specific errors here.

Reviewer #6: Yes

Reviewer #7: (No Response)

**********

6. Review Comments to the Author

Please use the space provided to explain your answers to the questions above. You may also include additional comments for the author, including concerns about dual publication, research ethics, or publication ethics. (Please upload your review as an attachment if it exceeds 20,000 characters)

Reviewer #6: (No Response)

Reviewer #7: Comments:

Modelling the impact of vector control methods on disease transmission is interesting to follow to understand the possible situation in the real world. Authors have duly responded to the comments and suggestions of reviewers in their revised manuscript.

I suggest some minor points in the revised manuscript.

Abstract section:

1. It is good to mention the region or the country at the end of the first sentence. “………in the malaria endemic regions in ……………….” options (a) across the globe (b)African region (c) a particular country in Africa, e.g. Uganda

2. In fig 4 legend, mention separately three successive LLIN campaigns.

3. Revise the reiteration of descriptions in the Result and Discussion sections.

4. The last sentence of the Conclusion should be removed and replaced with some recommendations to mitigate the selection pressure developed against the mosquito population due to prolonged utilization of pyrethroid-based LLINs in the Malaria control program.

**********

7. PLOS authors have the option to publish the peer review history of their article (what does this mean?). If published, this will include your full peer review and any attached files.

If you choose “no”, your identity will remain anonymous but your review may still be made public.

**Do you want your identity to be public for this peer review?**  For information about this choice, including consent withdrawal, please see our Privacy Policy.

Reviewer #6: No

Reviewer #7: No

**********

[NOTE: If reviewer comments were submitted as an attachment file, they will be attached to this email and accessible via the submission site. Please log into your account, locate the manuscript record, and check for the action link "View Attachments". If this link does not appear, there are no attachment files.]

To ensure your figures meet our technical requirements, please review our figure guidelines: https://journals.plos.org/plosone/s/figures

You may also use PLOS’s free figure tool, NAAS, to help you prepare publication quality figures: https://journals.plos.org/plosone/s/figures#loc-tools-for-figure-preparation.

NAAS will assess whether your figures meet our technical requirements by comparing each figure against our figure specifications.

# Author comment: Author response to Decision Letter 4

**DOI:** 10.1371/journal.pone.0353301.r008

21 May 2026

The responses to all reviewer comments have been provided in the "Responce to reviewer" report included among file attachments.

Attachment Submitted filename: Response_to_Reviewers_may_2026.pdf

# Peer review: Decision Letter 4

**DOI:** 10.1371/journal.pone.0353301.r009

**Authors:** Rajib Chowdhury

22 Jun 2026

Modelling the Impact of Mosquito Bed Net Utilization on Malaria Transmission and Evolution of Pyrethroid Resistance

PONE-D-25-51344R4

Dear Dr. Ivan,

We’re pleased to inform you that your manuscript has been judged scientifically suitable for publication and will be formally accepted for publication once it meets all outstanding technical requirements.

Within one week, you’ll receive an e-mail detailing the required amendments. When these have been addressed, you’ll receive a formal acceptance letter and your manuscript will be scheduled for publication.

An invoice will be generated when your article is formally accepted. Please note, if your institution has a publishing partnership with PLOS and your article meets the relevant criteria, all or part of your publication costs will be covered. Please make sure your user information is up-to-date by logging into Editorial Manager at Editorial Manager® and clicking the ‘Update My Information' link at the top of the page. For questions related to billing, please contact billing support.

If your institution or institutions have a press office, please notify them about your upcoming paper to help maximize its impact. If they’ll be preparing press materials, please inform our press team as soon as possible -- no later than 48 hours after receiving the formal acceptance. Your manuscript will remain under strict press embargo until 2 pm Eastern Time on the date of publication. For more information, please contact onepress@plos.org.

Kind regards,

Rajib Chowdhury, M.Sc.; MPH

Academic Editor

PLOS One

Additional Editor Comments (optional):

Reviewers' comments:

Reviewer's Responses to Questions

**Comments to the Author**

1. If the authors have adequately addressed your comments raised in a previous round of review and you feel that this manuscript is now acceptable for publication, you may indicate that here to bypass the “Comments to the Author” section, enter your conflict of interest statement in the “Confidential to Editor” section, and submit your "Accept" recommendation.

Reviewer #6: All comments have been addressed

Reviewer #7: All comments have been addressed

**********

2. Is the manuscript technically sound, and do the data support the conclusions?

The manuscript must describe a technically sound piece of scientific research with data that supports the conclusions. Experiments must have been conducted rigorously, with appropriate controls, replication, and sample sizes. The conclusions must be drawn appropriately based on the data presented.

Reviewer #6: (No Response)

Reviewer #7: Yes

**********

3. Has the statistical analysis been performed appropriately and rigorously?

Reviewer #6: (No Response)

Reviewer #7: Yes

**********

4. Have the authors made all data underlying the findings in their manuscript fully available?

The PLOS Data policy requires authors to make all data underlying the findings described in their manuscript fully available without restriction, with rare exception (please refer to the Data Availability Statement in the manuscript PDF file). The data should be provided as part of the manuscript or its supporting information, or deposited to a public repository. For example, in addition to summary statistics, the data points behind means, medians and variance measures should be available. If there are restrictions on publicly sharing data—e.g. participant privacy or use of data from a third party—those must be specified.

Reviewer #6: (No Response)

Reviewer #7: Yes

**********

5. Is the manuscript presented in an intelligible fashion and written in standard English?

PLOS ONE does not copyedit accepted manuscripts, so the language in submitted articles must be clear, correct, and unambiguous. Any typographical or grammatical errors should be corrected at revision, so please note any specific errors here.

Reviewer #6: (No Response)

Reviewer #7: Yes

**********

6. Review Comments to the Author

Please use the space provided to explain your answers to the questions above. You may also include additional comments for the author, including concerns about dual publication, research ethics, or publication ethics. (Please upload your review as an attachment if it exceeds 20,000 characters)

Reviewer #6: (No Response)

Reviewer #7: No further technical comments. However, Authors should check the flow of subheadings in Methodology, Results and Discussion sections. Equations should be thoroughly checked.

**********

7. PLOS authors have the option to publish the peer review history of their article (what does this mean?). If published, this will include your full peer review and any attached files.

If you choose “no”, your identity will remain anonymous but your review may still be made public.

**Do you want your identity to be public for this peer review?**  For information about this choice, including consent withdrawal, please see our Privacy Policy.

Reviewer #6: No

Reviewer #7: No

**********

# Editor report: Acceptance letter

**DOI:** 10.1371/journal.pone.0353301.r010

**Authors:** Rajib Chowdhury

PONE-D-25-51344R4

PLOS One

Dear Dr. Sseguya,

I'm pleased to inform you that your manuscript has been deemed suitable for publication in PLOS One. Congratulations! Your manuscript is now being handed over to our production team.

At this stage, our production department will prepare your paper for publication. This includes ensuring the following:

* All references, tables, and figures are properly cited

* All relevant supporting information is included in the manuscript submission,

* There are no issues that prevent the paper from being properly typeset

You will receive further instructions from the production team, including instructions on how to review your proof when it is ready. Please keep in mind that we are working through a large volume of accepted articles, so please give us a few days to review your paper and let you know the next and final steps.

Lastly, if your institution or institutions have a press office, please let them know about your upcoming paper now to help maximize its impact. If they'll be preparing press materials, please inform our press team within the next 48 hours. Your manuscript will remain under strict press embargo until 2 pm Eastern Time on the date of publication. For more information, please contact onepress@plos.org.

You will receive an invoice from PLOS for your publication fee after your manuscript has reached the completed accept phase. If you receive an email requesting payment before acceptance or for any other service, this may be a phishing scheme. Learn how to identify phishing emails and protect your accounts at https://explore.plos.org/phishing.

If we can help with anything else, please email us at customercare@plos.org.

Thank you for submitting your work to PLOS ONE and supporting open access.

Kind regards,

PLOS ONE Editorial Office Staff

on behalf of

Dr. Rajib Chowdhury

Academic Editor

PLOS One
